# Supplementary material for: A modular chemoenzymatic cascade strategy for the structure-customized assembly of ganglioside analogs
Source: Commun Chem. 2024 Jan 18;7:17. doi: 10.1038/s42004-024-01102-9 (PMC10796935; doi:10.1038/s42004-024-01102-9)
Supplement: Supplementary file 2 — Supporting Information [file 42004_2024_1102_MOESM2_ESM.docx]

**Supplementary Information**

**A Modular Chemoenzymatic Cascade Strategy for the Structure-Customized Assembly of Ganglioside Analogs**

**Xuefeng Jin^1,2†^, Hanchao Cheng^3,4†^, Xiaohui Chen^1^, Xuefeng Cao^5^, Cong Xiao^5^, Fengling Ding^5^, Huirong Qu^5^, Peng George Wang^4^, Yan Feng^1^, Guang-Yu Yang^1^***

^1^ State Key Laboratory of Microbial Metabolism, Joint International Research Laboratory of Metabolic & Developmental Sciences, School of Life Sciences and Biotechnology, Shanghai Jiao Tong University, Shanghai 200240, China.

^2^ Department of Clinical Pharmaceutics, The People’s Hospital of Guangxi Zhuang Autonomous Region, Nanning, 530021, China.

^3^ School of Food and Drug, Shenzhen Polytechnic University, Shenzhen 518055, China.

^4^ Department of Pharmacology, Key University Laboratory of Metabolism and Health of Guangdong, School of Medicine, Southern University of Science and Technology, Guangdong 518055, China.

^5^ Glycogene LLC, 10th Floor, Building 3, Wuhan Precision Medicine Industrial Base, Gaokeyuan Road, East Lake New Technology Development Zone, Wuhan 430074, China.

†These authors contributed equally to this work.

*Corresponding author’s email: yanggy@sjtu.edu.cn

**Table of Contents**

Ⅰ. Supplementary methods……………………..…………...……………………………………….3

1. General materials and methods………………………………………………………………..3

2. Construction, expression and purification of various enzymes applied in MOCECA strategy.3

Ⅱ. Experimental procedures…………………….………………………………………...…………4

1. Scalable and cost-effective preparation of D-sphingosines with high purity by chemical synthesis…………………………………………………………………………………………….4
2. Synthesis of fluoro-oligosaccharides by streamlined multi-enzymatic glycosylation system..8
3. Enzymatic transglycosylation synthesis of glycosylsphingosine derivatives……...……….10
4. Enzymatic acylation synthesis of gangliosides………………………...………………….12
5. Industrial and cost-effective preparation of GM1 derivatives with various sphingosines and fatty acids………………………………………………………………………………………….15

Ⅲ. HRMS spectrum of compounds in MOCECA strategy……………………………………….27

1. HRMS spectra of GM3βSph, GM2βSph, GM1βSph, GD3βSph, GD2βSph…………......…27
2. HRMS spectra of ganglioside including GM3, GM2, GM1, GD3, GD2…………………28
3. HRMS spectra of ganglioside GM1 derivatives………………………………………......29

Ⅳ. Component comparison and identification of GM1 commercialized drugs named Sygen™ based on MOCECA synthesized GM1 derivatives……………………………………………......33

1. Hydrolysis and purification procedure for SA_SCD enzymatic deacylation system……….33
2. HRMS and HPLC spectra for two components of purified Sygen™…………………......33
3. HRMS and HPLC spectra for glycosphingosine products of Sygen™ hydrolyzed by SA_SCD enzymatic deacylation system………………………...………………………….…………....…..34
4. HRMS and HPLC spectra for MOCECA synthesized GM1βSph (d18:1) and GM1βSph (d20:1) ………………………………………………………………………………...………......34
5. HRMS and HPLC spectra for glycosylsphingosine products of MOCECA synthesized GM1 derivatives mixed with GM1(d18:1/C18:0) and GM1(d20:1/C18:0) hydrolyzed by SCD enzymatic deacylation system………………………………………………………………………………...36
6. HRMS and HPLC spectra for glycosylsphingosine products of MOCECA synthesized GM1 derivatives mixed with GM1(d18:1/C18:0) and GM1(d18:1/C20:0) hydrolyzed by SCD enzymatic deacylation system………………………………………………………………………………...37

Ⅴ. Supplementary references…………………………………………………………………......37

**Ⅰ. Supplementary methods**

**1. General materials and methods**

^1^H and ^13^C NMR spectra of various purified compounds were recorded by 700 MHz Bruker Avance Neo NMR spectrometer or Agilent 400-MR DD2 NMR spectrometer. The high-resolution electrospray ionization (ESI) mass spectra of each compound were determined by Agilent & 1290 Infinity II / 6545 QTOF LC/MS. WATERS Acquity I-class VION IMS QTOF was used to analyze the information fragment peaks of compounds. The purity and components of the compounds were determined by EasySep-1020 high performance liquid chromatography (Unimicro Technologies). Various gangliosides and relevant derivatives were detected by waters Sunfire C18 color columns. Various glycosylsphingosines and relevant derivatives were detected by Welch ultimate XB-C18 column. Anisaldehyde sugar staining was used on silica gel plates in Thin-layer chromatography (TLC) detection. Adenosine 5'-triphosphate (ATP) was bought from Kaiping Genuine Biochemical Pharmaceutical (Guangzhou, China). Uridine 5'-triphosphate (UTP) and cytidine 5'-triphosphate (CTP) were from Meiya Pharmaceutical (Hangzhou, China). Acetylneuraminic acid (Neu5Ac), D-Galactose (Gal) and D-GalNAc were purchased from Sigma-Aldrich.

**2. Construction, expression and purification of various enzymes applied in MOCECA strategy**

Various recombinant enzymes including *Neisseria meningitidis* CMP-sialic acid synthetase (NmCSS)^1^{Yu, 2004 #1;Yu, 2004 #1}, *Pasteurella multocida* α2-3-sialyltransferase 1(PmST1)^2^, *Pasteurella multocida* inorganic pyrophosphatase (PmPpA)^3^, *Bifidobacteriumlongum* strain ATCC55813 *N*-acetylhexosamine-1-kinase (BLNahK)^4^{Li, 2011 #4}{Li, 2011 #4}, human UDP-GalNAc pyrophosphorylase (AGX1)^5^, *Campylobacter jejuni* 1-4GalNAcT (CjCgtA)^6^, *Escherichia coli* galactokinase (EcGalK)^7^, *Arabidopsisthaliana* UDP-sugar pyrophosphorylase (AtUSP)^8^, *Campylobacter jejuni*1-3-galactosyltransferase (CjCgtB)^9^, *Campylobacter jejuni* α2-3/8 sialyltransferase (CjCstⅡ)^10^, *Rhodococcus* strain M-777 endoglycoceramidase Ⅱ (EGC II) D314Y/E351S^11,12^, *Shewanella alga G8* sphingolipid ceramide N-deacylase (SA_SCD)^13^ {Cheng, 2008 #16}{Cheng, 2008 #18}used in multi enzyme cascade system were expressed and purified using a universal improved procedure base on previous literature reports.

Based on the reported literature and NCBI data retrieval, we identified and synthesized the gene sequences of various enzymes used in MOCECA. Twelve synthetic genes were used to ligate with various pET series vectors. EcGalK, AtUSP, AGX1 were connected with pET15b vector. PmST1 was connected with pET23a (+) vector. SA_SCD was connected with pET23b vector. EGC-II D314Y/E351S was connected with pET28a (+) vector. NmCSS, PmPpA, BLNahK, CjCgtA, CjCgtB, CstII was connected with pET22b (+) vector. The twelve constructed plasmids were transformed into E.coli BL21 (DE3) cells separately.

Transformants were selected for sequencing verification and the right object were inoculated into the medium and cultured overnight at 37 ℃ for 12 h. The E.coli strains were then transferred to Luria-Bertani (LB) medium (10 g/L tryptone, 5 g/L yeast extract, and 10 g/L NaCl) with kanamycin or ampicillin (100 μg/ml) and continued for incubation at 37 ℃. When the OD_600_ nm of the culture reached 0.8-1.0, IPTG with final concentration of 0.1 mM was added at 20 ℃ for 18 h to induce the over-expression. What's special is that SA_SCD was induced at 16 ℃. Bacterial cells were harvested by centrifugation at 7000 rpm for 20 min.

Cells were resuspended with 50mM Tris-HCl (pH 8.0) buffer and crushed by high pressure homogenizer with the pressure of 680~720 bar at 4℃. The supernatant was collected after centrifugation at 7500 rpm at 4℃ for 25 min. The 0.45 um filter membrane was used to remove cell debris. 5 column volume binding buffer including 25 mM Tris-HCl (pH8.0), 10 mM Imidazole and 200 mM NaCl was used for pre equilibration before the supernatant was loaded onto the Ni2+-NTA affinity column. Nonspecific binding proteins were removed through washing with 10 column volumes of binding buffer and 10 column volumes of washing buffer including 25 mM Tris-HCl (pH8.0), 40 mM Imidazole and 200 mM NaCl. The target protein was collected by elution buffer (25 mM Tris-HCl with pH8.0, 200 mM Imidazole, 200 mM NaCl).

The purified enzymes were dialyzed to remove imidazole and 10% glycerol (v/v) was added. They were stored at -80℃ for subsequent catalytic reactions.

**Ⅱ. Experimental procedures**

1. **Scalable and cost-effective preparation of D-sphingosines with high purity by chemical synthesis**

***Methyl N-(tert-butoxycarbonyl)-O-(tert-butyldimethylsilyl)-L-serinate (29)***

L-serine was used as the initial substrate, which is cheap and easily available. Acetyl chloride (385 mL, 5.415 mol, 2.845 equiv) was added dropwise to ice cold methanol (1.45 L) and stirred for 30 minutes at 5-10 ℃. L-serine (200.0 g, 1.903 mol, 1 equiv) was added in batches and stirred for 2 h at 80 ℃. The reaction solution was cooled to room temperature and dried to obtain solid materials. A solution of dichloromethane (DCM, 2 L) and triethylamine (TEA, 593 mL, 4.266 mol) were added and then (Boc)_2_O (468.0 g, 2.144 mol) was added dropwise ＜15 ℃. After stirring at 20-30 ℃ overnight, the solution was washed with saturated NaHCO_3_, brine and dried over Na_2_SO_4_. Compound **28** (409.0 g, 1.865 mol) was obtained through filtration and concentration.

Compound **28** (409.0 g, 1.865 mol), 5 L of DCM and imidazole (214.0 g, 3.143 mol, 1.685 equiv) were combined in a 50 L reaction vessel. TBDMSCl (470.0 g, 3.118 mol, 1.672 equiv) and DCM (4.5 L) were added dropwise at 5-10 ℃. After stirring at room temperature overnight, HCl solution (1.0 M, 10 L) was added to stir for 10 min. Treated with DCM for extraction, the organic layer was washed with saturated NaCl and dried over Na_2_SO_4_.The crude product was purified by a column (silica gel, petroleum ether/ethyl acetate = 40:1) to give compound **29** (622.0 g, 1.865 mol)^14,15^.

***Methyl N-(tert-butoxycarbonyl)-O-(tert-butyldimethylsilyl)-L-serinate (29)***

Compound **29**, white solid after lyophilization, 622.0 g, 98% yield. ^1^H NMR (400 MHz, CDCl_3_) δ 5.34 (d, *J* = 8.0 Hz, 1H), 4.35 (d, *J* = 8.7 Hz, 1H), 4.03 (d, *J* = 9.9 Hz, 1H), 3.81 (d, *J* = 10.9 Hz, 1H), 3.73 (s, 3H), 1.45 (s, 9H), 0.85 (s, 9H), 0.03 (s, 3H), 0.01 (s, 3H). ^13^C NMR (100 MHz, CDCl_3_) δ 171.42, 155.60, 80.02, 63.93, 55.74, 52.38, 28.47, 25.84, 18.32, −5.40, −5.52.

ESI-HRMS (m/z): [M+H]**^+^** calculated for C_15_H_32_NO_5_Si, 334.2044; Found, 334.2033.

***tert-Butyl (S,E)-(1-((tert-butyldimethylsilyl)oxy)-3-oxooctadec-4-en-2-yl)carbamate (30)***

To a solution of dimethyl methyl phosphonate (795.0 g, 6.407 mol, 3.425 equiv) in anhydrous THF (2.8L) was added *n*-BuLi (2.52 L, 2.5 M in hexane, 6.300 mol, 3.378 equiv) slowly over 30 min at −75℃. After stirring for 1 h at the same temperature, compound **29** (622.0 g, 1.865 mol) in anhydrous THF (530 mL) was added slowly at the same temperature and stirred for 1h. After the reaction was completed, the solution was slowly warmed to 0 ℃ and quenched with 6 L aqueous NH_4_Cl at 0 ℃. The solution was extracted with EtOAc. The organic layer was concentrated and used further without purification. The organic layer was washed with saturated NaCl and dried over Na_2_SO_4_, and then was concentrated to give compound **30** (738.0 g, 1.734 mol).

***tert-Butyl(S)-(1-((tert-butyldimethylsilyl)oxy)-4-(dimethoxyphosphoryl)-3-oxobutan-2-yl)carbamate (30)***

Compound **30**, white solid after lyophilization, 738.0 g, 93% yield. ^1^H NMR (400 MHz, CDCl_3_) δ 5.50 (d, *J* = 7.1 Hz, 1H), 4.40 (t, *J* = 3.5 Hz, 1H), 4.03(dd, *J* = 10.3, 3.4 Hz, 1H), 3.80 (dd, *J* = 10.3, 4.0 Hz, 1H), 3.75 (dd, *J* = 11.3, 2.1 Hz, 6H), 3.36 (dd, *J* = 22.3, 11.5 Hz, 1H), 3.10 (dd, *J* = 22.3, 11.5 Hz, 1H), 1.40 (s, 9H), 0.82 (s, 9H), 0.00 (s, 6H). ^13^C NMR (100 MHz, CDCl_3_) δ 199.74 (d, *J* = 6.5 Hz), 155.29, 80.01, 63.03, 61.94, 53.16 (t, *J* = 6.9 Hz), 38.59 (d, *J* = 131.7 Hz), 28.38, 25.80, 18.23, −5.53, −5.55.

ESI-HRMS(m/z): [M+H]**^+^** calculated for C_17_H_37_NO_7_PSi, 426.2071; Found, 426.2055.

***tert-Butyl (S,E)-(1-((tert-butyldimethylsilyl)oxy)-3-oxooctadec-4-en-2-yl)carbamate (31)***

***tert-Butyl (S,E)-(1-((tert-butyldimethylsilyl)oxy)-3-oxoicos-4-en-2-yl)carbamate (32)***

Compound **30** (738.0 g, 1.734 mol), LiCl 121.0 g, 2.854 mol, 1.646 equiv), *n*-tetradecanal (310.0 g, 1.460 mol) or *n*-hexadecaldehyde (351.0 g, 1.460 mol) was suspended in THF (8 L). Then *n*-tetradecanaland *n*-hexadecaldehyde was prepared according to previously reported procedure^16^. Then, TEA (402 mL, 2.892 mol) was added. The reaction was allowed to stir at 15-20 ℃ for 5 hours. The solution was washed by 2 L of citric acid (2 M) and extracted the aqueous layer with EtOAc. The organic layer was combined and washed with saturated NaCl. The concentrated crude product was purified by a short flash column (silica gel, petroleum ether/ethyl acetate = 40:1) to give compound **31** (720.0 g, 1.407 mol) or compound **32** (759.0 g, 1.406 mol) as a white material.

***tert-Butyl(S,E)-(1-((tert-butyldimethylsilyl)oxy)-3-oxooctadec-4-en-2-yl)carbamate (31)***

Compound **31**, white solid after lyophilization, 720.0 g, 81% yield. ^1^H NMR (400 MHz, CDCl_3_) δ 6.96 (dt, *J* = 15.9, 7.0 Hz, 1H), 6.27 (d, *J* = 15.7 Hz, 1H), 5.54 (d, *J* = 7.1 Hz, 1H), 4.56-4.52 (m, 1H), 3.97 (dd, *J* = 10.3, 3.2 Hz, 1H), 3.83 (dd, *J* = 10.3, 4.3 Hz, 1H), 2.25-2.19 (m, 2H), 1.44 (s, 9H), 1.31-1.26 (m, 22H), 0.89-0.84 (m, 12H), 0.01 (d, *J* = 2.2 Hz, 6H).^13^C NMR (100 MHz, CDCl_3_) δ 196.62, 155.49, 149.39, 127.04, 79.74, 63.80, 59.62, 32.78, 32.05, 29.79, 29.65, 29.53, 29.48, 29.34, 28.48, 28.14, 25.88, 25.83, 22.82, 18.33, 14.24, −5.43, −5.46.

ESI-HRMS (m/z): [M+H]**^+^** calculated for C_29_H_58_NO_4_Si, 512.4130; Found, 512.4135.

***tert-Butyl (S,E)-(1-((tert-butyldimethylsilyl)oxy)-3-oxoicos-4-en-2-yl)carbamate (32)***

Compound **32**, white solid after lyophilization, 759.0 g, 81% yield. ^1^H NMR (400 MHz, CDCl_3_) δ 6.96(dt, *J* = 15.8, 6.8 Hz, 1H), 6.27 (d, *J* = 15.8 Hz, 1H), 5.54 (d, *J* = 7.5 Hz, 1H), 4.54 (t, *J* = 3.5 Hz, 1H), 3.97(dd, *J* = 10.3, 2.9 Hz, 1H), 3.83 (dd, *J* = 10.3, 4.6 Hz, 1H),2.25-2.19 (m, 2H), 1.44 (s, 9H), 1.44 (s, 26H), 0.89-0.84 (m, 12H), 0.00 (s, 6H). ^13^C NMR (100 MHz, CDCl_3_) δ 196.50, 155.36, 149.26, 126.91, 79.61, 63.68, 59.50, 32.65, 31.93, 29.70, 29.67, 29.64, 29.53, 29.41, 29.37, 29.22, 28.36, 28.01, 25.75, 22.69, 18.20, 14.12, −5.56, −5.58.

ESI-HRMS (m/z): [M+H]**^+^** calculated for C_31_H_62_NO_4_Si, 540.4443; Found, 540.4446.

***tert-Butyl ((2S,3R,E)-1,3-dihydroxyoctadec-4-en-2-yl)carbamate (35)***

***tert-Butyl ((2S,3R,E)-1,3-dihydroxyicos-4-en-2-yl)carbamate (36)***

We prepared compounds **35** and **36** according to a similar procedure^17^. To a solution of LiAlH(O*^t^*Bu)_3_ (762.0 g, 2.997 mol, 2.130 equiv) in ethanol (7.5 L) at −78 ºC was added dropwise a solution of compound **31**(720.0 g, 1.407 mol) or compound **32** (759.0 g, 1.406 mol) in EtOH (7.5 L) to give compound **33** or compound **34** at −78 ºC for 2 h. After the temperature rised to about 0 ℃,1 M HCl (17 L) was added and the solution was allowed to stir at 25 ℃ for 17 h until the substrate was basically consumed. The reaction mixture was extracted with ethyl acetate (20 L). The organic layer was washed with NaHCO_3_, brine and then dried over Na_2_SO_4_. The concentrated crude product was purified by a column (silica gel, petroleum ether/ethyl acetate = 15:1 and 1:1) to give compound **35** (529.0 g, 1.324 mol) or **36** (565.0 g, 1.321 mol) as a white waxy material.

***tert-Butyl ((2S,3R,E)-1,3-dihydroxyoctadec-4-en-2-yl)carbamate (35)***

Compound **35**, white waxy material, 529.0 g, 94% yield. ^1^H NMR (400 MHz, CDCl_3_) δ 5.78 (dt, *J* = 15.3, 6.6 Hz, 1H), 5.53 (dd, *J* = 15.3, 6.6 Hz, 1H), 5.30 (s, 1H), 4.30 (s, 1H), 3.93 (dd, *J* = 11.4, 3.7 Hz, 1H), 3.71 (dd, *J* = 11.4, 3.4 Hz, 1H), 3.6 (s, 1H), 2.08-2.03 (m, 2H), 1.45 (s, 9H), 1.39-1.26 (m, 22H), 0.88 (t, *J* = 6.6 Hz, 3H). ^13^C NMR (100 MHz, CDCl_3_) δ 156.38, 134.30, 129.05, 79.94, 74.94, 62.78, 55.55, 32.43, 32.06, 29.82, 29.75, 29.62, 29.49, 29.35, 29.25, 28.51, 22.82, 14.25.

ESI-HRMS (m/z): [M+H]**^+^** calculated for C_23_H_46_NO_4_, 400.3421; Found, 400.3450.

***tert-Butyl ((2S,3R,E)-1,3-dihydroxyicos-4-en-2-yl)carbamate (36)***

Compound **36**, white waxy material, 565.0 g, 94% yield. ^1^H NMR (400 MHz, CDCl_3_) δ 5.78 (dt, *J* = 15.5, 7.2 Hz, 1H), 5.53 (dd, *J* = 15.8, 6.7 Hz, 1H), 5.30 (s, 1H), 4.30 (s, 1H), 3.94 (dd, *J* = 11.3, 3.6 Hz, 1H), 3.71 (dd, *J* = 11.4, 3.5 Hz, 1H), 3.60 (s, 1H), 2.08-2.01 (m, 2H), 1.45 (s, 9H), 1.27-1.26 (m, 26H), 0.88 (t, *J* = 6.4 Hz, 3H). ^13^C NMR (100 MHz, CDCl_3_) δ 156.40, 134.29, 129.08, 79.92, 75.00, 62.79, 55.52, 32.43, 32.06, 31.35, 29.83, 29.75, 29.62, 29.50, 29.34, 29.24, 28.51, 27.86, 27.55, 22.83, 14.25.

ESI-HRMS (m/z): [M+H]**^+^** calculated forC_25_H_50_NO_4_, 428.3734; Found, 428.3768.

***(2S,3R,E)-2-Aminooctadec-4-ene-1,3-diol (D-sphingosine (d18:1), (16))***

***(2S,3R,E)-2-Aminoicos-4-ene-1,3-diol (D-sphingosine (d20:1), (17))***

To an ice cooled solution of the corresponding compound **35** (529.0 g, 1.324 mol) or compound **36** (565.0 g, 1.321 mol) in MeOH (6 L) was added dropwise neat acetyl chloride (520 mL, 7.314 mol) at 5-15℃. After stirring at 25 ℃ for 15h, the reaction mixture was concentrated in vacuo. The crude was then dissolved in 2 L methanol and mixed with NaOH solution (1 M, 8 L), then stirring at 10-20℃ for 20 min. This mixture was extracted with DCM (2 L) for three times. The combined organic layers were dried over Na_2_SO_4_ and concentrated to afford the crude product. The crude product was purified by recrystallization with DCM and ethyl acetate to give **D-sphingosine(d18:1)** (238.0 g, 0.795 mol) or **D-sphingosine(d20:1)** (260.0 g, 0.794 mol).

***(2S,3R,E)-2-Aminooctadec-4-ene-1,3-diol (16)***

D-sphingosine (d18:1) **(16)**, white solid after lyophilization, 238.0 g, 60% yield. ^1^H NMR (700 MHz, MeOD) δ 5.73 (m, 1H), 5.49 (m, 1H), 3.97 (t, *J* = 6.7 Hz, 1H), 3.67 (dd, *J* = 10.7, 4.5 Hz, 1H), 3.49 (dd, *J* = 10.8, 6.9 Hz, 1H), 2.76-2.74 (m, 1H), 2.09-2.06 (m, 2H), 1.43-1.39 (m, 2H), 1.33-1.29 (m, 20H), 0.90 (t, *J* = 6.9 Hz, 3H).^13^C NMR (176 MHz, MeOD) δ 133.84, 129.40, 73.65, 62.88, 56.60, 32.05, 31.70, 29.42, 29.39, 29.37, 29.26, 29.10, 29.00, 28.97, 22.36, 13.08.

ESI-HRMS(m/z): [M+H]**^+^** calculated for C_18_H_38_NO_2_, 300.2897; Found, 300.2897.

***(2S,3R,E)-2-Aminoicos-4-ene-1,3-diol (17)***

D-sphingosine (d20:1) **(17)**, white solid after lyophilization, 260.0 g, 60%. ^1^H NMR (700 MHz, MeOD) δ 5.76-5.72 (m, 1H), 5.51-5.48 (m, 1H), 3.99 (t, *J* = 6.6 Hz, 1H), 3.67 (dd, *J* = 10.7, 4.5 Hz, 1H), 3.50 (dd, *J* = 10.8, 6.9 Hz, 1H), 2.78-2.76 (m, 1H), 2.08-2.06 (m, 2H), 1.42-1.39 (m, 2H), 1.33-1.28 (m, 24H), 0.90 (t, *J* = 7.1 Hz, 3H).^13^C NMR (176 MHz, MeOD) δ 133.88, 129.31, 73.55, 62.73, 56.62, 32.05, 31.69, 29.41, 29.38, 29.26, 29.10, 29.00, 28.97, 22.35, 13.09.

ESI-HRMS(m/z): [M+H]**^+^** calculated for C_20_H_42_NO_2_, 328.3210; Found,328.3223.

1. **Synthesis of fluoro-oligosaccharides by streamlined multi-enzymatic glycosylation system**

**Synthesis of GM3-F by α2-3 sialylation system**


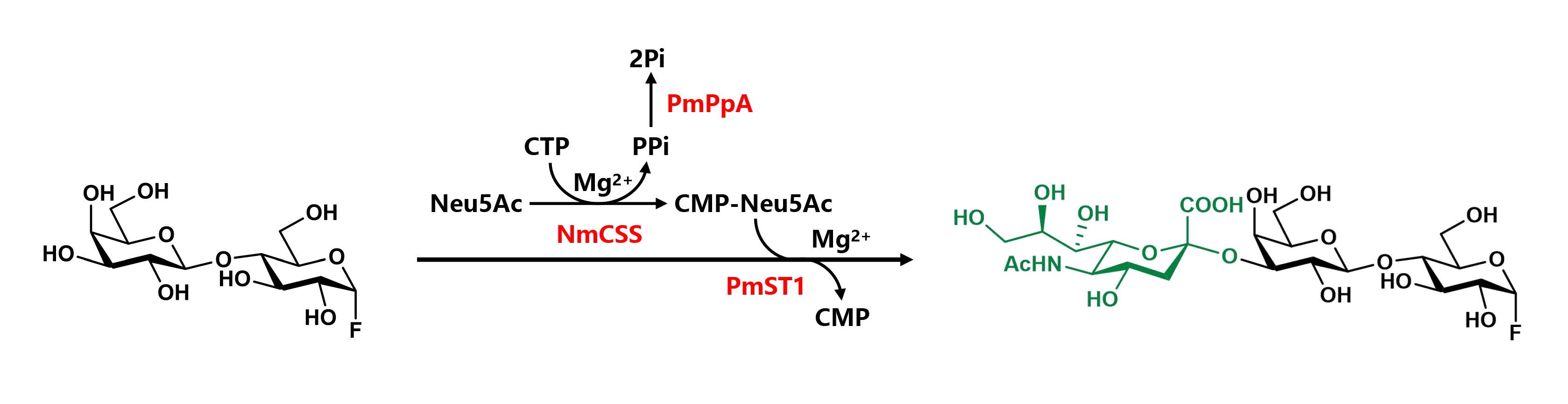


Lac-F (1.38 g, 4.00 mmol), MgCl_2_ (20 mM), Neu5Ac (2.10 g, 6.80 mmol) and CTP (3.38 g, 6.41 mmol) was added in 100 mM Tris-HCl buffer (pH 9.0) with the sialylation system containing NmCSS (52 mg), PmST1 (50 mg) and PmPpA (44 mg) in total volume of 200 mL. The solution was incubated at 150 rpm in an agitator-reactor at 37 ℃ for 4.5 h to form GM3-F. The reaction was detected by TLC (EtOAc/MeOH/H_2_O/HOAc, 4:2:1:0.2, v/v) and stained with *p*-anisaldehyde sugar stain. After removing the precipitates by centrifugation of 10000 rpm for 10 min, the supernatant containing GM3-F was confirmed by HRMS and collected for the next step. ESI-HRMS (m/z): [M+Na]**^+^** calculated for C_23_H_38_FNNaO_18_, 658.1965; found, 658.1967.

**Synthesis of GM2-F by β1-4N-acetylgalactosaminylation system**


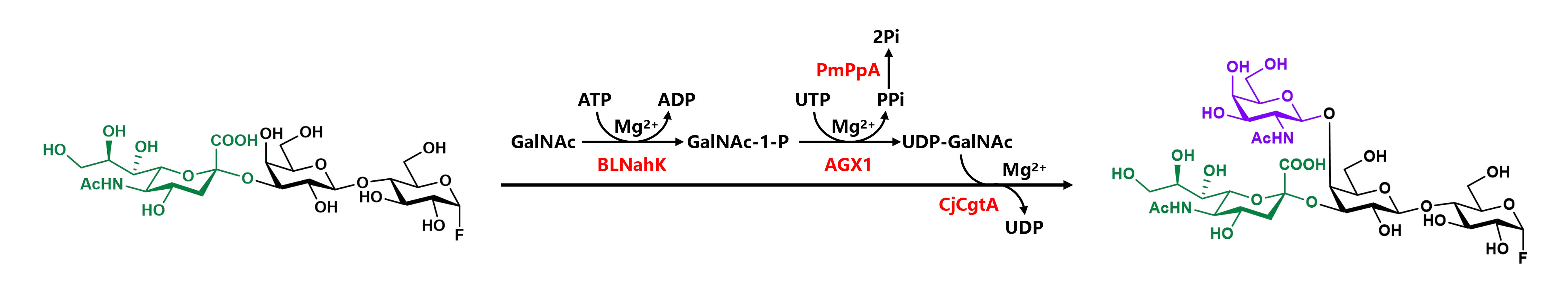


GalNAc (1.06 g, 4.80 mmol), ATP (3.32 g, 6.02 mmol) and UTP (3.08 g, 5.60 mmol) were added to the GM3-F solution, and the parameters were guaranteed to contain 20 mM MgCl_2_ and 100 mM Tris-HCl buffer (pH 9.0) in 400 ml volume. After adding BLNahK (40 mg), AGX1 (30 mg), PmPpA (20 mg) and CjCgtA (100 mg), the solution was incubated at 150 rpm in an agitator-reactor at 37 ℃ for 3 h to form GM2-F. The reaction was detected by TLC (EtOAc/MeOH/H_2_O/HOAc, 4:2:1:0.2, v/v) and stained with *p*-anisaldehyde sugar stain. After removing the precipitates by centrifugation of 10000 rpm for 10 min, the supernatant containing GM2-F was confirmed by HRMS and collected for the next step. ESI-HRMS (m/z): [M+Na]**^+^** calculated for C_31_H_51_FN_2_NaO_23_, 861.2759; found, 861.2757.

**Synthesis of GM1-F by β1-3 galactosylation system**


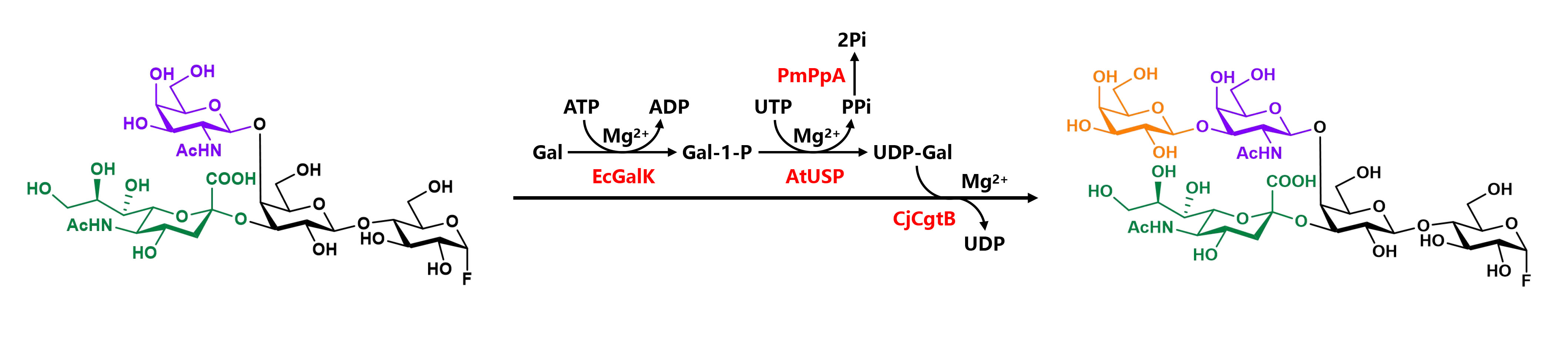


Gal (0.79 g, 4.40 mmol), ATP (2.43 g, 4.40 mmol) and UTP (2.42 g, 4.40 mmol) were directly added to GM2-F solution. The parameters were guaranteed to contain 20 mM MgCl_2_ and 100 mM Tris-HCl buffer (pH 8.0) in 800 mL volume. After adding EcGalK (60 mg), AtUSP (52 mg), PmPpA (20 mg) and CjCgtB (120 mg), the solution was incubated at 150 rpm in an agitator-reactor at 37 ℃ for 6 h to form GM1-F. The reaction was detected by TLC (EtOAc/MeOH/H_2_O/HOAc, 50:33:17:0.2, v/v) and stained with p-anisaldehyde sugar stain. After removing the precipitates by centrifugation of 10000 rpm for 10 min, the preliminary purified GM1-F was confirmed by HRMS and collected for the next step. ESI-HRMS (m/z): [M+Na]**^+^** calculated for C_37_H_61_FN_2_NaO_28_, 1023.3287; found, 1023.3283.

**Synthesis of GD3-F by α2-8 sialylation system**


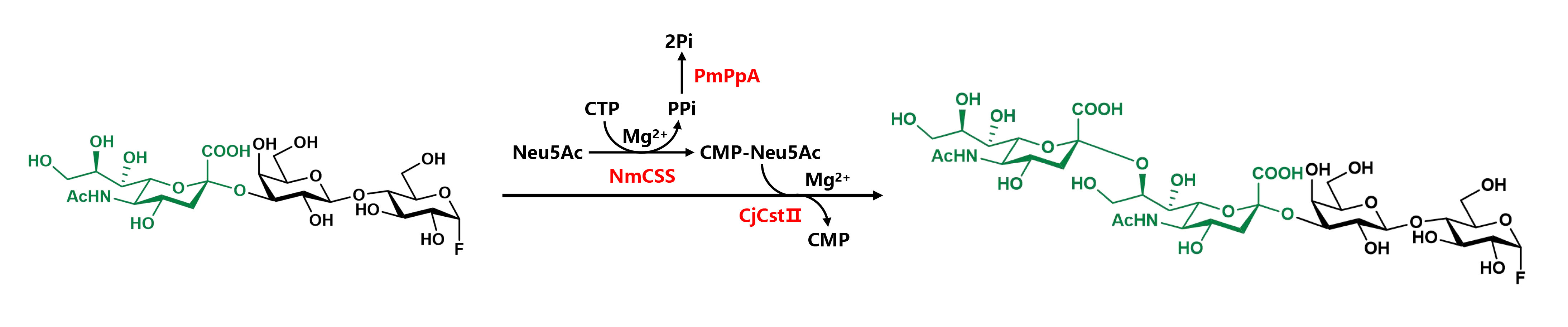


Neu5Ac (2.48 g, 8.00 mmol) and CTP (4.22 g, 8.00 mmol) were added to the GM3-F solution, and the parameters were guaranteed to contain 20 mM MgCl_2_ and 100 mM Tris-HCl buffer (pH 9.0) in 400 mL volume. After adding NmCSS (60 mg), PmPpA (52 mg) and CjCstⅡ (48 mg), the solution was incubated at 150 rpm in an agitator-reactor at 37 ℃ overnight to form GD3-F. The reaction was detected by TLC (EtOAc/MeOH/H_2_O/HOAc, 4:2:1:0.2, v/v) and stained with *p*-anisaldehyde sugar stain. After removing the precipitates by centrifugation of 10000 rpm for 10 min, the supernatant containing GD3-F was confirmed by HRMS and collected for the next step. ESI-HRMS (m/z): [M+Na]**^+^** calculated for C_34_H_55_FN_2_NaO_26_, 949.2919; found, 949.2903.

**Synthesis of GD2-F by β1-4N-acetylgalactosaminylation system**


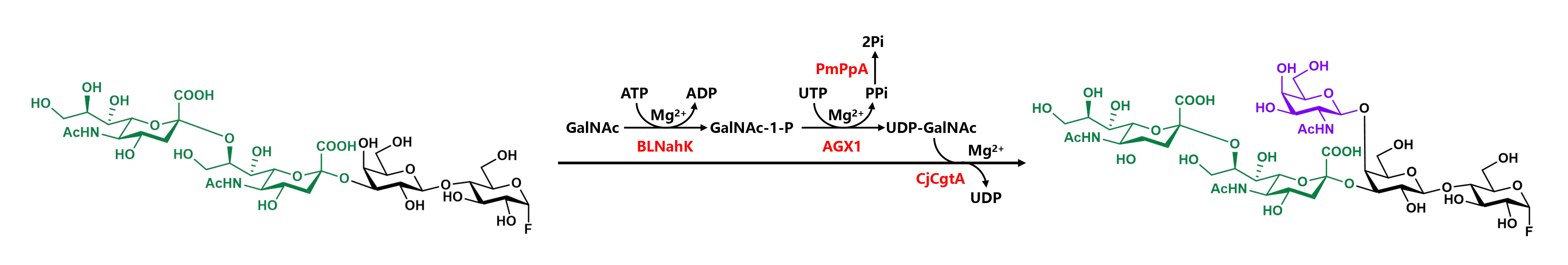


GalNAc (1.33 g, 6.00 mmol), ATP (3.31 g, 6.00 mmol) and UTP (3.30 g, 6.00 mmol) were added to the GD3-F solution, and the parameters were guaranteed to contain 20 mM MgCl_2_ and 100 mM Tris-HCl buffer (pH 9.0) in 800 mL volume. After adding BLNahK (40 mg), AGX1 (42 mg), PmPpA (20 mg) and CjCgtA (100 mg), the solution was incubated at 150 rpm in an agitator-reactor at 37 ℃ for 6 h to form GD2-F. The reaction was detected by TLC (EtOAc/MeOH/H_2_O/HOAc, 4:2:1:0.2, v/v) and stained with *p*-anisaldehyde sugar stain. After removing the precipitates by centrifugation of 10000 rpm for 10 min, the supernatant containing GD2-F was confirmed by HRMS and collected for the next step. ESI-HRMS (m/z): [M+Na]**^+^** calculated for C_42_H_69_FN_3_NaO_31_, 1153.3791; found, 1153.3790.

1. **Enzymatic transglycosylation synthesis of glycosylsphingosine derivatives**
2. **General synthesis procedure for glycosylsphingosines including GM3βSph, GM2βSph, GM1βSph, GD3βSph and GD2βSph**

The solution containing diverse fluoro-oligosaccharides by centrifugation, D-sphingosine (d18:1) (2.40g, 8.00 mmol) was in 100 mM NaAc buffer (pH 5.3) with 10% ethanol (v/v) for solubilization. The reaction was incubated 10 h or overnight after adding the enzyme (800 mg). Various designated glycosylsphingosines was synthesized by EGC-II E351S/D314Y. TLC and UV-HPLC were used to monitor until the reaction was completed. When the best yield was obtained, the supernatant was obtained by centrifugation at 10000 rpm for 10 min and the same volume of prechilled EtOH was added to quench the reaction.

1. **Purification procedure for diverse glycosylsphingosines**

After incubation with the same volume of ice-cold EtOH at 4℃ for 2 h, the mixture was centrifuged at 8000 rpm to remove the precipitates. The supernatant was concentrated to an appropriate volume and then loaded on C18 cartridges for purification. The impurities in GM3βSph, GM2βSph, GM1βSph, and GD2βSph reaction solutions such as salt and sugar nucleotide were removed with ultrapure water and 50% gradient methanol. The purified GM3βSph, GM2βSph, GM1βSph and GD2βSph were eluted with 80% gradient methanol separately. The impurities in GD3βSph reaction solutions including salt and sugar nucleotide including were removed with ultrapure water and 40% gradient methanol. 50% gradient methanol was used to retrieve GT3βSph. Then the purified GD3βSph was eluted with 65% gradient methanol. Various target compounds were concentrated and freeze-dried to obtain the pure diverse glycosylsphingosines. Their structures were identified by ^1^H and ^13^C NMR.

***(5-Acetamido-3,5-dideoxy-D-glycero-α-D-galacto-2-nonulopyranosylonic acid)-(2→3)-β-D-galactopyranosyl-(1→4)-β-D-glucopyranosyl-(1→1)-(2S, 3R, 4E)-2-amino-4-octadecene-1,3-diol (6)***

GM3βSph **(6)**, white solid after lyophilization, 3.20 g, 92% yield.

^1^H NMR (700 MHz, MeOD) δ 5.86 (dt, *J* = 15.6, 7.2 Hz, 1H), 5.48 (dd, *J* = 15.6, 6.8 Hz, 1H), 4.41 (d, *J* = 7.8 Hz, 1H), 4.36 (d, *J* = 7.8 Hz, 1H), 4.30 (d, *J* = 5.6 Hz, 1H), 4.05 (dd, *J* = 9.8, 3.1 Hz, 1H), 3.98-3.36 (m, 19H), 2.86 (dd, *J* = 12.6, 4.0 Hz, 1H), 2.10 (m, 2H), 2.01 (s, 3H, CH_3_), 1.72 (t, *J* = 12.0 Hz, 1H), 1.44-1.26 (m, 22 H, 11×CH_2_), 0.90 (t, J = 7.1 Hz, 6H, 2×CH_3_). ^13^C NMR (176 MHz, MeOD)δ 174.17, 173.48, 135.28, 135.03, 127.21, 126.96, 103.72, 102.40, 99.66, 79.19, 76.33, 75.75, 75.20, 74.84, 73.57, 73.10, 71.59, 69.80, 69.60, 69.41, 68.75, 67.89, 67.61, 65.95, 63.34, 61.36, 60.23, 58.31, 57.09, 55.36, 52.58, 40.74, 31.98, 31.67, 29.39, 29.36, 29.24, 29.07, 29.00, 28.79, 22.33, 21.19, 13.04.

ESI HRMS(m/z): [M+H]**^+^** calculated for C_41_H_75_N_2_O_20_, 915.4908; found, 915.4920.

***(2-Acetamido-2-deoxy-β-D-galactopyranosyl)-(1→4)-(5-acetamido-3,5-dideoxy-D-glycero-α-D-galacto-2-nonulopyranosylonic acid)-(2→3)-β-D-galactopyranosyl-(1→4)-β-D-glucopyranosyl-(1→1)-(2S, 3R, 4E)-2-amino-4-octadecene-1,3-diol (7)***

GM2βSph **(7)**, white solid after lyophilization, 3.83 g, 91% yield.

^1^H NMR (700 MHz, MeOD) δ5.86 (dt, *J* = 16.0, 7.2 Hz, 1H), 5.48 (dd, *J* = 16.0, 7.2 Hz, 1H), 4.82 (d, *J* = 8.2 Hz, 1H), 4.41 (d, *J* = 7.6 Hz, 1H), 4.35 (d, *J* = 7.6 Hz, 1H), 4.32 (t, J = 5.7 Hz, 1H), 4.14 (d, *J* = 3.2 Hz, 1H), 4.02-3.37 (m, 25H), 2.72 (dd, *J* = 12.6, 4.8 Hz, 1H), 2.09 (m, 2H), 2.01 (s, 3H, CH_3_), 1.88 (t, *J* = 11.9 Hz, 1H), 1.43-1.24 (m, 22 H, 11×CH_2_), 0.88 (t, J = 7.0 Hz, 3H, CH_3_). ^13^C NMR (176 MHz, MeOD) δ 174.33, 173.70, 173.36, 135.42, 126.79, 103.36, 102.86, 102.28, 101.96, 79.52, 77.53, 75.12, 74.93, 74.85, 74.70, 74.22, 73.63, 73.02, 72.57, 71.99, 70.07, 69.62, 69.49, 68.95, 68.34, 68.19, 65.71, 63.89, 61.58, 60.33, 60.22, 55.23, 52.77, 52.33, 37.13, 31.94, 31.63, 30.95, 30.85, 29.35, 29.31, 29.30, 29.19, 29.02, 28.97, 28.73, 22.30, 22.25, 21.22, 13.04.

ESI HRMS(m/z): [M+H]**^+^** calculated for C_49_H_88_N_3_O_25_, 1118.5701; found, 1118.5750.

***β-D-Galactopyranosyl-(1→3)-(2-acetamido-2-deoxy-β-D-galactopyranosyl)-(1→4)-(5-acetamido-3,5-dideoxy-D-glycero-α-D-galacto-2-nonulopyranosylonic acid)-(2→3)-β-D-galactopyranosyl-(1→4)-β-D-glucopyranosyl-(1→1)-(2S, 3R, 4E)-2-amino-4-octadecene-1,3-diol (8)***

GM1βSph **(8)**, white solid after lyophilization, 4.35 g, 92% yield.

^1^H NMR (700 MHz, MeOD) δ 5.78 (dt, *J*= 15.2, 6.9 Hz, 1H), 5.40 (dd, *J* = 15.6, 6.9 Hz, 1H), 4.49 (m, 1H), 4.36 (d, *J* = 7.6 Hz, 1H), 4.32 (d, *J* = 7.6 Hz, 1H), 4.27-4.24 (m, 2H), 4.06-4.03 (m, 2H), 3.93-3.21(m, 29H), 2.64 (dd, *J* = 13.0, 4.8 Hz, 1H), 2.01 (m, 2H), 1.93-1.90 (m, 6H, 2xCH_3_), 1.80 (t, *J* = 12.0 Hz, 1H), 1.35-1.20 (m, 22 H, 11xCH_2_), 0.80 (t, *J* = 7.2Hz, 3H, CH_3_). ^13^C NMR (176 MHz, MeOD) δ 174.33, 173.84, 173.49, 135.29, 126.89, 105.23, 103.49, 102.75, 102.34, 102.02, 81.56, 79.61, 77.62, 75.19, 75.11, 74.96, 74.76, 74.51, 74.30, 73.69, 73.20, 73.10, 72.02, 71.13, 69.64, 69.48, 69.05, 68.82, 68.30, 68.24, 65.69, 64.00, 61.59, 61.01, 60.44, 60.32, 55.30, 52.40, 51.35, 37.21, 31.99, 31.67, 29.39, 29.35, 29.24, 29.07, 29.02, 28.79, 22.44, 22.33, 21.27, 13.07.

ESI HRMS(m/z): [M+H]**^+^** calculated for C_55_H_98_N_3_O_30_, 1280.6230; found, 1280.6266.

***(5-Acetamido-3,5-dideoxy-D-glycero-α-D-galacto-2-nonulopyranosylonic acid)-(2→8)-(5-acetamido-3,5-dideoxy-D-glycero-α-D-galacto-2-nonulopyranosylonic acid)-(2→3)-β-D-galactopyranosyl-(1→4)-β-D-glucopyranosyl-(1→1)-(2S, 3R, 4E)-2-amino-4-octadecene-1,3-diol (9)***

GD3βSph **(9)**, white solid after lyophilization, 2.76 g, 86% yield.

^1^H NMR (700 MHz, MeOD) δ 5.85 (dt, *J* = 15.5, 7.0 Hz, 1H), 5.53 (dd, *J* = 15.5, 7.0 Hz, 1H), 4.51 (d, *J* = 7.8 Hz, 1H), 4.36 (d, *J* = 7.8 Hz, 1H), 4.34 (t, *J* = 5.5 Hz, 1H), 4.15-3.40 (m, 34H), 2.92 (d, *J* = 12.0, 1H), 2.68 (s, 1H), 2.11-2.08 (m, 2H), 2.03-2.01 (m, 6H, 2xCH_3_), 1.73-1.68 (m, 2H), 1.44-1.29 (m, 22 H, 11xCH_2_),0.90 (t, *J* = 6.9 Hz, 3H, CH_3_). ^13^C NMR (176 MHz, MeOD) δ 175.54, 175.11, 136.50, 128.75, 104.92, 103.92, 102.64, 101.48, 80.38, 78.40, 77.04, 76.87, 76.59, 76.12, 75.59, 74.65, 74.61, 73.02, 71.44, 70.79, 70.33, 69.67, 67.90, 64.71, 64.46, 63.31, 62.89, 61.86, 61.53, 56.75, 54.26, 54.04, 49.57, 42.63, 33.44, 33.11, 30.86, 30.84, 30.80, 30.69, 30.64, 30.51, 30.45, 30.36, 30.28, 28.14, 26.96, 23.78, 23.06, 22.69, 14.48.

ESI HRMS(m/z): [M+H]**^+^** calculated for C_52_H_92_N_3_O_28_, 1206.5862; found, 1206.5897.

***2-Acetamido-2-deoxy-β-D-galactopyranosyl-(1→4)-(5-Acetamido-3,5-dideoxy-D-glycero-α-D-galacto-2-nonulopyranosylonic acid)-(2→8)-(5-acetamido-3,5-dideoxy-D-glycero-α-D-galacto-2-nonulopyranosylonic acid)-(2→3)-β-D-galactopyranosyl-(1→4)-β-D-glucopyranosyl-(1→1)-(2S, 3R,4E)-2-amino-4-octadecene-1,3-diol (10)***

GD2βSph **(10)**, white solid after lyophilization, 3.03 g, 84% yield.

^1^H NMR (700 MHz, MeOD) δ5.85 (dt, *J* = 15.7, 6.8 Hz, 1H), 5.49 (dd, *J* = 15.7, 6.8 Hz, 1H), 4.46 (d, *J* = 7.7 Hz, 1H), 4.34 (d, *J* = 7.7 Hz, 1H), 4.27 (t, *J* = 5.6 Hz, 1H), 4.20-3.40 (m, 34H), 2.86 (d, *J* = 11.4, 1H), 2.75 (d, *J* = 11.4, 1H), 2.10 (m, 2H), 2.04-2.01 (m, 9H, 3xCH_3_), 1.80-1.73 (m, 2H), 1.44-1.28 (m, 22 H, 11xCH_2_),0.93 (t, *J* = 7.2 Hz, 3H, CH_3_). ^13^C NMR (176 MHz, MeOD) δ 174.10, 173.48, 173.45, 173.41, 173.39, 135.12, 127.21, 103.56, 103.00, 102.41, 100.96, 100.55, 79.12, 77.11, 76.82, 75.17, 74.90, 74.64, 74.37, 74.01, 73.26, 73.17, 72.67, 71.42, 70.05, 69.94, 69.45, 68.90, 68.28, 68.22, 66.29, 63.23, 62.08, 61.23, 60.31, 60.04, 55.33, 53.22, 52.83, 52.68, 48.12, 40.71, 39.83, 31.98, 31.67, 29.38, 29.35, 29.23, 29.06, 28.99, 28.81, 22.33, 22.21, 21.72, 21.20, 13.04.

ESI HRMS(m/z): [M+H]**^+^** calculated for C_60_H_105_N_4_O_33_, 1409.6656; found, 1409.6693.

1. **Enzymatic acylation synthesis of gangliosides**
2. **General synthesis procedure for gangliosides including GM3 (1), GM2 (2), GM1 (3), GD3 (4), GD2 (5)**

Diverse glycosylsphingosines (2.00 mmol) and stearic acid (C18:0, 853 mg, 3.00 mmol) were incubated in 800 ml of Tris-HCl buffer (50 mM, pH 7.5) with 10% DME (v/v) in the presence of 160 mg purified SA_SCD. The whole catalytic system was carried out at 37 ℃ for 6 h. TLC and UV-HPLC were used to monitor until the reaction was completed. When the best yield was obtained, the supernatant containing target compound was collected by centrifugation of 10000 rpm for 30 min at 4 °C

1. **Purification procedure for diverse gangliosides including GM3 (1), GM2 (2), GM1 (3), GD3 (4), GD2 (5)**

Various gangliosides were purified by C18 cartridges and silica gel filtration column. For purification of GM3, 5 column volumes of acetonitrile in water (v/v) with gradients of 20% was used to remove impurities. Acetonitrile with 60% gradient was used to retrieve GM3βSph. GM3 and parts of fatty acids were eluted with 80% acetonitrile in water (v/v). For purification of GM2, 5 column volumes of acetonitrile in water (v/v) with gradients of 20% were used to remove impurities. Acetonitrile with 40% gradient was used to retrieve GM2βSph. GM2 and parts of fatty acids were eluted with 60% acetonitrile in water (v/v). For purification of GM1, 5 column volumes of methanol in water (v/v) with gradients of 30% were used to remove impurities. Methanol with 75% gradient was used to retrieve GM1βSph. GM1 and parts of fatty acids were eluted with 90% methanol in water (v/v). After concentration by rotary evaporation, silica gel filtration column was used to remove fatty acids for further separation. 5 column volumes of petroleum ether, 5 column volumes of petroleum ether/dichloromethane (v/v) and 5 column volumes of dichloromethane were used to remove stearic acid. The pure GM3, GM2 and GM1 were eluted by dichloromethane/methanol/H_2_O (70:28:4, v/v/v). For purification of GD3, 5 column volumes of methanol in water (v/v) with gradients of 30% was used to remove impurities. Methanol with 50% gradient was used to retrieve GD3βSph. GD3 was eluted with 65% methanol in water (v/v). For purification of GD2, 5 column volumes of methanol in water (v/v) with gradients of 30% were used to remove impurities. Methanol with 45% gradient was used to retrieve GD2βSph. GD2 was eluted with 60% methanol in water (v/v). After concentration by rotary evaporation, silica gel filtration column was used to remove fatty acids for further separation. 10 column volumes of petroleum ether and 10 column volumes of ethyl acetate were used to remove stearic acid. The pure GD3 and GD2 were eluted by ethyl acetate/methanol (2:1, v/v). The fractions containing pure products were concentrated and lyophilized to obtain various gangliosides as white powders. Their structures were identified by ^1^H and ^13^C NMR.

***(5-Acetamido-3,5-dideoxy-D-glycero-α-D-galacto-2-nonulopyranosylonic acid)-(2→3)-β-D-galactopyranosyl-(1→4)-β-D-glucopyranosyl-(1→1)-(2S, 3R, 4E)-2-octadecanamido-4-octadecene-1,3-diol(1)***

GM3 **(1)**, white solid after lyophilization, 1.63 g, 69% yield. ^1^H NMR (700 MHz, MeOD) δ 5.68 (dt, *J* = 15.3, 7.3 Hz, 1H), 5.44 (dd, *J* = 15.3, 7.7 Hz, 1H), 4.42 (d, *J* = 7.7 Hz, 1H), 4.30 (d, *J* = 7.7 Hz, 1H), 4.18 (dd, *J* = 10.1, 4.8 Hz, 1H), 4.08-3.40 (m, 19H), 3.28 (t, *J* = 8.4 Hz, 1H), 2.85 (dd, *J* = 12.6, 3.5 Hz, 1H), 2.17 (t, *J* = 7.5 Hz, 2H), 2.05-1.98 (m, 5H), 1.74-1.70 (m, 1H), 1.62-1.54 (m, 2H), 1.40-1.26 (m, 46 H, 23×CH_2_), 0.90 (t, J = 7.1 Hz, 6H, 2×CH_3_). ^13^C NMR (176 MHz, MeOD)δ 174.56, 174.09, 173.57, 168.97, 167.94, 133.63, 132.18, 130.95, 129.99, 129.45, 128.47, 103.69, 103.09, 99.71, 79.43, 76.25, 75.64, 75.06, 74.80, 73.53, 73.43, 71.56, 69.43, 68.69, 68.52, 67.97, 67.71, 67.60, 66.28, 65.25, 63.18, 61.32, 60.39, 53.32, 52.55, 48.13, 40.66, 35.97, 32.07, 31.70, 31.68, 29.48, 29.45, 29.42, 29.41, 29.37, 29.31, 29.24, 29.11, 29.08, 29.06, 29.03, 25.78, 22.35, 21.17, 13.05.

ESI HRMS(m/z): [M+H]**^+^** calculated for C_59_H_109_N_2_O_21_, 1181.7517; found, 1181.7485.

***(2-Acetamido-2-deoxy-β-D-galactopyranosyl)-(1→4)-(5-acetamido-3,5-dideoxy-D-glycero-α-D-galacto-2-nonulopyranosylonic acid)-(2→3)-β-D-galactopyranosyl-(1→4)-β-D-glucopyranosyl-(1→1)-(2S, 3R, 4E)-2-octadecanamido-4-octadecene-1,3-diol (2)***

GM2 **(2)**, white solid after lyophilization, 1.94 g, 70% yield. ^1^H NMR (700 MHz, MeOD) δ 5.68 (dt, *J* = 15.1, 7.5 Hz, 1H), 5.44 (dd, J = 15.1, 7.5 Hz, 1H), 4.42 (d, *J* = 8.0 Hz, 1H), 4.30 (d, J = 8.0 Hz, 1H), 4.18 (dd, *J* = 10.0, 4.1 Hz, 1H), 4.14 (d, *J* = 2.8 Hz, 1H), 4.07 (t, *J* = 8.2 Hz, 1H), 4.02-3.26 (m, 25H), 2.73 (dd, J = 13.0, 5.0 Hz, 1H), 2.17 (t, J = 7.5 Hz, 2H), 2.05-2.01 (m, 5H), 1.90 (t, J = 12.0 Hz, 1H), 1.61-1.55 (m, 2H), 1.40-1.26 (m, 46 H, 23×CH_2_), 0.90 (t, J = 7.2 Hz, 6H, 2×CH_3_). ^13^C NMR (176 MHz, MeOD) δ 174.55, 174.25, 173.75, 173.35, 133.65, 129.99, 103.52, 103.07, 102.87, 102.06, 79.87, 77.62, 75.05, 75.00, 74.89, 74.74, 74.21, 73.70, 73.46, 72.72, 72.00, 71.56, 69.67, 69.02, 68.50, 68.44, 68.28, 66.28, 64.30, 63.97, 61.63, 60.41, 60.31, 53.31, 52.88, 52.37, 38.81, 37.17, 35.97, 32.07, 31.70, 31.68, 29.48, 29.45, 29.42, 29.41, 29.37, 29.31, 29.24, 29.11, 29.08, 29.07, 29.03, 25.78, 22.35, 22.20, 21.17, 13.05.

ESI HRMS(m/z): [M+H]**^+^** calculated for C_67_H_122_N_3_O_26_, 1384.8311; found, 1384.8326.

***β-D-Galactopyranosyl-(1→3)-(2-acetamido-2-deoxy-β-D-galactopyranosyl)-(1→4)-(5-acetamido-3,5-dideoxy-D-glycero-α-D-galacto-2-nonulopyranosylonic acid)-(2→3)-β-D-galactopyranosyl-(1→4)-β-D-glucopyranosyl-(1→1)-(2S, 3R, 4E)-2-octadecanamido-4-octadecene-1,3-diol (3)***

GM1 **(3)**, white solid after lyophilization, 2.20 g, 71% yield. ^1^H NMR (700 MHz, MeOD) δ 5.68 (dt, *J* = 15.2, 7.6 Hz, 1H), 5.44 (dd, *J* = 15.2, 7.6 Hz, 1H), 4.92 (d, J = 8.7 Hz, 1H), 4.44 (d, *J* = 7.8 Hz, 1H), 4.41 (d, *J* = 8.2 Hz, 1H), 4.29 (d, *J* = 8.2 Hz, 1H), 4.19-3.26 (m, 36H), 2.73 (dd, *J* = 12.8, 4.6 Hz, 1H), 2.17 (t, J = 7.5 Hz, 2H), 2.03-1.99 (m, 8H), 1.90 (t, *J* = 12.0 Hz, 1H), 1.59-1.56 (m, 2H), 1.38-1.29 (m, 49 H), 0.90 (t, J = 6.6 Hz, 6H, 2×CH_3_). ^13^C NMR (176 MHz, MeOD) δ 174.10, 173.48, 173.45, 173.41, 173.39, 161.89, 161.69, 161.49, 135.12, 127.21, 103.56, 103.00, 102.41, 100.96, 100.55, 79.12, 77.11, 76.82, 75.17, 74.90, 74.64, 74.37, 74.01, 73.26, 73.17, 72.67, 71.42, 70.05, 69.94, 69.45, 68.90, 68.28, 68.22, 66.29, 63.23, 62.08, 61.23, 60.31, 60.04, 55.33, 53.22, 52.83, 52.68, 48.12, 40.71, 39.83, 31.98, 31.67, 29.38, 29.35, 29.23, 29.06, 28.99, 28.81, 22.33, 22.21, 21.72, 21.20, 13.04.

ESI HRMS(m/z): [M+H]**^+^** calculated for C_73_H_132_N_3_O_31_, 1546.8839; found, 1546.8799.

***(5-Acetamido-3,5-dideoxy-D-glycero-α-D-galacto-2-nonulopyranosylonic acid)-(2→8)-(5-acetamido-3,5-dideoxy-D-glycero-α-D-galacto-2-nonulopyranosylonic acid)-(2→3)-β-D-galactopyranosyl-(1→4)-β-D-glucopyranosyl-(1→1)-(2S, 3R, 4E)-2-octadecanamido-4-octadecene-1,3-diol (4)***

GD3 **(4)**, white solid after lyophilization, 1.95 g, 65% yield. ^1^H NMR (700 MHz, MeOD) δ5.68 (dt, *J* = 15.4, 7.2 Hz, 1H), 5.44 (dd, *J* = 15.4, 7.2 Hz, 1H), 4.48 (s, 1H), 4.30 (d, *J* = 7.9 Hz, 1H), 4.19-3.28 (m, 27H), 2.97 (s, 1H), 2.51 (s, 1H), 2.17 (t, *J* = 7.5 Hz, 2H), 2.04-1.99 (m, 8H), 1.63-1.53 (m, 4H), 1.39-1.28 (m, 46 H, 23×CH_2_), 0.90 (t, *J* = 7.2 Hz, 6H, 2×CH_3_). ^13^C NMR (176 MHz, MeOD) δ174.49, 174.04, 174.03, 133.61, 129.96, 126.80, 113.47, 103.04, 75.03, 74.74, 73.41, 72.42, 71.49, 69.81, 68.49, 62.97, 60.72, 60.07, 53.26, 35.95, 32.06, 31.68, 31.66, 30.85, 29.47, 29.43, 29.43, 29.41, 29.39, 29.36, 29.35, 29.29, 29.23, 29.09, 29.06, 29.01, 25.76, 22.33, 22.32, 21.46, 21.25, 13.05, 13.04.

ESI HRMS(m/z): [M+Na]**^+^** calculated for C_70_H_125_N_3_NaO_29_, 1494.8291; found, 1494.8237.

***2-Acetamido-2-deoxy-β-D-galactopyranosyl-(1→4)-(5-Acetamido-3,5-dideoxy-D-glycero-α-D-galacto-2-nonulopyranosylonic acid)-(2→8)-(5-acetamido-3,5-dideoxy-D-glycero-α-D-galacto-2-nonulopyranosylonic acid)-(2→3)-β-D-galactopyranosyl-(1→4)-β-D-glucopyranosyl-(1→1)-(2S, 3R,4E)-2-octadecanamido-4-octadecene-1,3-diol (5)***

GD2 **(5)**, white solid after lyophilization, 2.01 g, 60% yield. ^1^H NMR (700 MHz, MeOD) δ5.68(dt, J = 15.0, 7.2 Hz, 1H), 5.44 (dd, J = 15.0, 7.2 Hz, 1H), 4.43 (d, *J* = 8.1 Hz, 1H), 4.29 (d, J = 8.1 Hz, 1H), 4.19-3.38 (m, 33H), 3.28 (t, *J* = 8.8 Hz, 1H), 2.86 (d, *J* = 12.0, 1H), 2.58 (d, *J* = 12.0, 1H), 2.17 (t, *J* = 8.0 Hz, 2H), 2.03-2.01 (m, 8H), 1.89 (s, 3H, CH_3_), 1.66-1.55 (m, 4H), 1.40-1.26 (m, 46 H, 23×CH_2_), 0.90 (t, *J* = 7.1 Hz, 6H, 2×CH_3_). ^13^C NMR (176 MHz, MeOD) δ 180.56, 175.99, 175.51, 174.94, 170.41, 135.09, 131.44, 105.09, 104.52, 81.00, 76.49, 76.29, 76.17, 76.11, 75.61, 74.90, 72.99, 70.82, 69.96, 69.73, 69.37, 64.46, 64.07, 64.00, 63.98, 62.81, 61.70, 61.67, 61.60, 60.69, 54.74, 37.42, 33.52, 33.14, 33.13, 30.93, 30.90, 30.87, 30.85, 30.82, 30.75, 30.71, 30.68, 30.64, 30.56, 30.53, 30.48, 30.38, 30.37, 30.36, 30.27, 28.17, 28.15, 27.22, 26.95, 24.18, 23.80, 23.78, 23.77, 23.03, 22.69, 14.50.

ESI HRMS(m/z): [M+H]**^+^** calculated for C_78_H_140_N_4_O_34_, 1676.9343; found, 1676.9353

**5. Industrial and cost-effective preparation of GM1 derivatives with various sphingosines and fatty acids**

**1) Preparation of GM1-F fluoro-oligosaccharides by through streamlined multi-enzymatic glycosylation in hectogram scale**

**Industrial synthesis of GM3-F by α2-3 sialylation system**


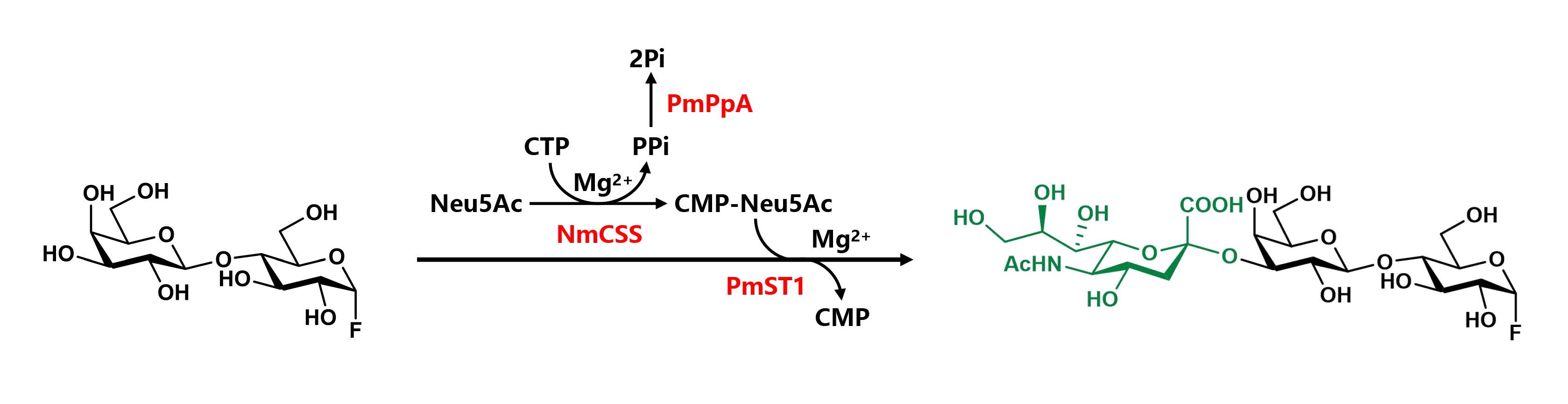


Lac-F (41.28 g, 120 mmol), MgCl_2_ (20 mM), Neu5Ac (63.04 g, 204 mmol) and CTP (101.19 g, 192 mmol) was added in 10 mM Tris-HCl buffer (pH 9.0) with the sialylation system containing NmCSS (1.56g), PmST1 (1.50 g) and PmPpA (1.32 g) in total volume of 6 L. The whole catalytic system was carried out in a 10 L bioreactor. 1M NaOH and 1M HCl loaded separately into two reservoirs in bioreactor were used for automatic and real-time control of pH value at 9.0. The solution was incubated at 150 rpm in bioreactor at 37 ℃ for 8 h to form GM3-F. The reaction was detected by TLC and stained with *p*-anisaldehyde sugar stain. After removing the precipitates by centrifugation of 8000 rpm for 10 min, the supernatant containing GM3-F was confirmed by HRMS and collected for the next step.

**Industrial synthesis of GM2-F by β1-4N-acetylgalactosaminylation system**


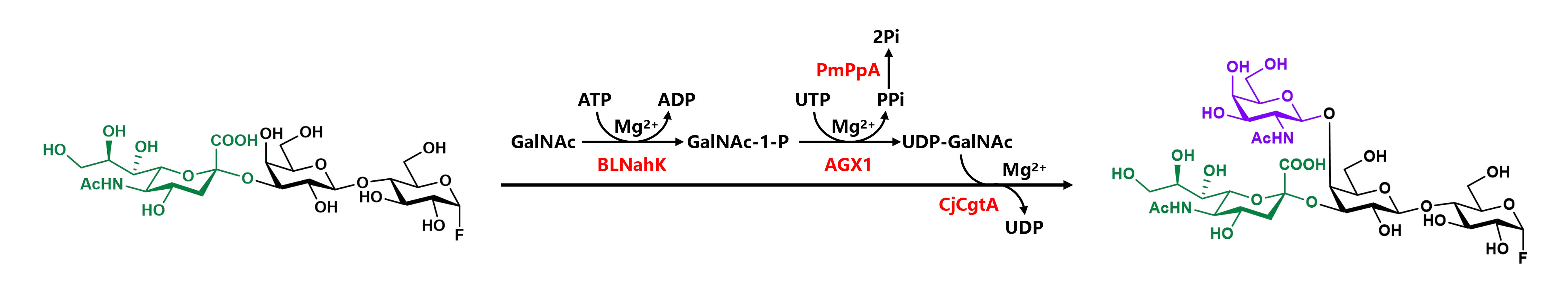


GalNAc (31.83 g, 144 mmol), ATP (99.21 g, 180 mmol) and UTP (92.4 g, 168 mmol) were added to the GM3-F solution, and the parameters were guaranteed to contain 20 mM MgCl_2_ and 10 mM Tris-HCl buffer (pH 9.0) in 12 L volume. The whole catalytic system was carried out in a 20 L bioreactor. 1M NaOH and 1M HCl loaded separately into two reservoirs in bioreactor were used for automatic and real-time control of pH value at 9.0. After adding BLNahK (1.20 g), AGX1 (0.90 g), PmPpA (0.60 g) and CjCgtA (3.00 g), the solution was incubated at 150 rpm in bioreactor at 37 ℃ for 6 h to form GM2-F. The reaction was detected by TLC and stained with *p*-anisaldehyde sugar stain. After removing the precipitates by centrifugation of 8000 rpm for 10 min, the supernatant containing GM2-F was confirmed by HRMS and collected for the next step.

**Industrial synthesis of GM1-F by β1-3 galactosylation system**


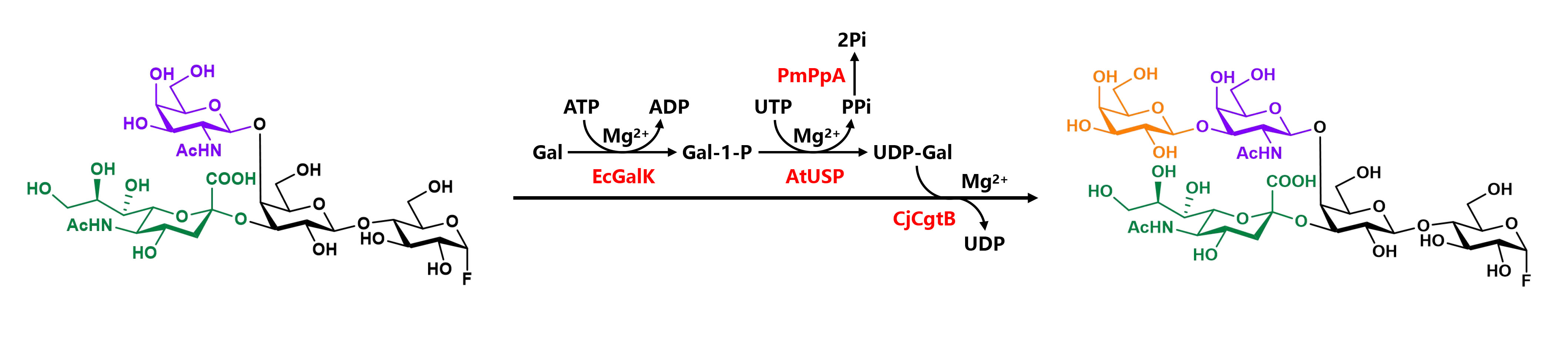


Gal (23.78 g, 132 mmol), ATP (72.75 g, 132 mmol) and UTP (72.61 g, 132 mmol) were directly added to GM2-F solution. The parameters were guaranteed to contain 20 mM MgCl_2_ and 10 mM Tris-HCl buffer (pH 8.0) in 24 L volume. The whole catalytic system was carried out in a 50 L bioreactor. 1M NaOH and 1M HCl loaded separately into two reservoirs in bioreactor were used for automatic and real-time control of pH value at 8.0. After adding EcGalK (1.80 g), AtUSP (1.56 g), PmPpA (0.60 g) and CjCgtB (3.6 g), the solution was incubated at 150 rpm in bioreactor at 37 ℃ for 8 h to form GM1-F. The reaction was detected by TLC and stained with p-anisaldehyde sugar stain. After removing the precipitates by centrifugation of 8000 rpm for 10 min, the preliminary purified GM1-F was confirmed by HRMS and collected for the next step.

**2) Industrial enzymatic transglycosylation synthesis of glycosylsphingosine derivatives GM1βSph (d18:1)**

24 L solution containing GM1-F by centrifugation, D-sphingosine (d18:1) (71.88 g, 240.00 mmol) was in 100 mM NaAc buffer (pH 5.3) with 10% ethanol(v/v) for solubilization. The reaction was incubated in a 50 L bioreactor with 100 rpm at 37 °C overnight after adding the enzyme (24.00 g). GM1βSph (d18:1) was synthesized by EGC-II E351S/D314Y. TLC and UV-HPLC were used to monitor until the reaction was completed. When the best yield was obtained, the supernatant containing target compound was collected by centrifugation of 10000 rpm for 30 min at 4 °C.

**3) Enzymatic transglycosylation synthesis of glycosylsphingosine derivatives GM1βSph (d20:1) derivatives at 10-gram scale**

The solution containing 2.0 L GM1-F by centrifugation, D-sphingosine (d20:1) (6.55 g, 20.00 mmol) was in 100 mM NaAc buffer (pH 5.3) with 10% ethanol(v/v) for solubilization. The reaction was incubated in a 5 L bioreactor with 100 rpm at 37 °C overnight after adding the enzyme (2.0 g). GM1βSph (d20:1) were synthesized by EGC-II E351S/D314Y. TLC and UV-HPLC were used to monitor until the reaction was completed. When the best yield was obtained, the supernatant containing target compound was collected by centrifugation of 10000 rpm for 30 min at 4℃.

**4)** **Purification procedure for glycosylsphingosine derivatives GM1βSph (d18:1) and GM1βSph (d20:1)**

GM1βSph (d18:1) and GM1βSph (d20:1) were firstly filtered by a series of microporous membranes to remove various impurities. 0.45 um microporous membrane was used to filter the supernatant. Then remaining macromolecular proteins were further removed by ultrafiltration of 5 KDa filtration membrane. After filtering again with 0.45um microporous membrane, nanofiltration with filtration membrane of 200 Da pore diameter is used to remove salt ions and other small molecules. The final filtrate was concentrated to an appropriate volume and then loaded on C18 cartridges for further purification. The impurities in reaction solutions of GM1βSph (d18:1) or GM1βSph (d20:1) derivatives such as remaining salt and sugar nucleotide were removed with ultrapure water and 50% gradient methanol. The purified GM1βSph (d18:1) or GM1βSph (d20:1) was eluted with 80% gradient methanol separately. Targeted compounds were concentrated and freeze-dried to obtain the pure diverse glycosylsphingosines. Their structures were identified by ^1^H and ^13^C NMR.

***β-D-Galactopyranosyl-(1→3)-(2-acetamido-2-deoxy-β-D-galactopyranosyl)-(1→4)-(5-acetamido-3,5-dideoxy-D-glycero-α-D-galacto-2-nonulopyranosylonic acid)-(2→3)-β-D-galactopyranosyl-(1→4)-β-D-glucopyranosyl-(1→1)-(2S, 3R, 4E)-2-amino-4-octadecene-1,3-diol (8)***

GM1βSph (d18:1) **(8)**, white solid after lyophilization, 130.31 g, 92% yield. ^1^H NMR (700 MHz, MeOD) δ 5.78 (dt, *J* = 15.2, 7.2 Hz, 1H), 5.40 (dd, *J* = 15.4, 6.7 Hz, 1H), 4.36 (d, *J* = 7.7 Hz, 1H), 4.32 (d, *J* = 8.0 Hz, 1H), 4.27 (d, *J* = 7.9 Hz, 1H), 4.24 (t, *J* = 5.5 Hz, 1H), 4.04 (t, *J* = 9.8 Hz, 2H), 3.93-3.22 (m, 33H), 2.63 (dd, *J* = 12.8, 4.8 Hz, 1H), 2.01 (dd, *J* = 16.7, 6.9 Hz, 2H), 1.92 (s, 3H), 1.90 (s, 3H), 1.80 (t, *J* = 12.2 Hz, 1H), 1.35-1.31 (m, 2H), 1.23-1.19 (m, 20H), 0.80 (t, *J* = 6.9 Hz, 3H).^13^C NMR (176 MHz, MeOD) δ 174.32, 173.85, 173.49, 135.27, 126.90, 105.24, 103.50, 102.75, 102.34, 102.02, 81.57, 79.62, 77.62, 75.19, 75.10, 74.96, 74.75, 74.51, 74.30, 73.69, 73.20, 73.09, 72.01, 71.12, 69.63, 69.48, 69.05, 68.81, 68.30, 68.24, 65.69, 64.00, 61.60, 61.00, 60.44, 60.31, 55.29, 52.39, 51.35, 37.20, 31.99, 31.67, 29.40, 29.36, 29.25, 29.07, 29.03, 28.80, 22.43, 22.34, 21.27, 13.07.

ESI HRMS(m/z): [M+H]**^+^** calculated for C_55_H_98_N_3_O_30_, 1280.6230; found, 1280.6264.

***β-D-Galactopyranosyl-(1→3)-(2-acetamido-2-deoxy-β-D-galactopyranosyl)-(1→4)-***

***(5-acetamido-3,5-dideoxy-D-glycero-α-D-galacto-2-nonulopyranosylonic acid)-(2→3)-β-D-galactopyranosyl-(1→4)-β-D-glucopyranosyl-(1→1)-(2S, 3R, 4E)-2-amino-4-eicosene-1,3-diol (18)***

GM1βSph (d20:1) **(18)**, white solid after lyophilization, 10.98 g, 91% yield. ^1^H NMR (700 MHz, MeOD) δ 5.74 (dt, *J* = 15.2, 7.2 Hz, 1H), 5.41 (dd, *J* = 15.5, 7.1 Hz, 1H), 4.38 (d, *J* = 7.6 Hz, 1H), 4.34 (d, *J* = 8.0 Hz, 1H), 4.26 (d, *J* = 7.9 Hz, 1H), 4.24 (t, *J* = 5.5 Hz, 1H), 4.09-4.07 (t, *J* = 9.8 Hz, 2H), 3.95-3.08 (m, 33H), 2.66 (dd, *J* = 12.4, 4.7 Hz, 1H), 2.02 (dd, *J* = 15.7, 6.9 Hz, 2H), 1.94 (s, 3H), 1.92 (s, 3H), 1.83 (t, *J* = 12.2 Hz, 1H), 1.36-1.33 (m, 2H), 1.26-1.21 (m, 24H), 0.83 (t, *J* = 6.9 Hz, 3H).^13^C NMR (176 MHz, MeOD) δ 174.28, 173.84, 173.46, 134.82, 128.10, 105.24, 103.51, 102.73, 102.56, 102.06, 81.59, 79.75, 77.61, 75.13, 75.09, 74.95, 74.74, 74.50, 74.25, 73.69, 73.19, 71.99, 71.31, 71.11, 69.64, 69.04, 68.83, 68.33, 68.28, 67.91, 63.98, 61.59, 61.49, 61.00, 60.38, 55.06, 52.38, 51.33, 37.67, 37.16, 32.01, 31.67, 29.38, 29.35, 29.24, 29.07, 28.99, 28.87, 26.33, 22.39, 22.33, 22.23, 21.20, 13.05.

ESI HRMS(m/z): [M+H]**^+^** calculated for C_57_H_102_N_3_O_30_, 1308.6543; found, 1308.6576.

**5) Industrial synthesis procedure for GM1 (d18:1/C18:0) (3) in hectogram scale**

GM1βSph (d18:1) (130.31 g, 101.76 mmol), stearic acid (C18:0) (43.42 g, 152.62 mmol) were incubated in 50 L of Tris-HCl buffer (10 mM, pH 7.5) with 10% DME, which containing 10 g purified SA_SCD. The whole catalytic system was carried out in a 100 L bioreactor with 120 rpm at 37 ℃ overnight. 1M NaOH and 1M HCl loaded separately into two reservoirs in bioreactor were used for automatic and real-time control of pH value at 7.5. TLC and UV-HPLC were used to monitor until the reaction was completed. When the best yield was obtained, the supernatant containing target compound was collected by centrifugation of 10000 rpm for 30 min at 4 ℃.

**6) General synthesis procedure for GM1 derivatives including GM1 (d18:1/C16:0) (19), GM1 (d18:1/C20:0) (20), GM1 (d18:1/C16:1) (21), GM1 (d18:1/C18:1) (22), GM1 (d20:1/C16:0) (23), GM1 (d20:1/C18:0) (24), GM1 (d20:1/C20:0) (25), GM1 (d20:1/C16:1) (26), GM1 (d20:1/C18:1) (27)**

GM1βSph (d18:1) (1.56 g, 1.22 mmol), different kinds of saturated and unsaturated fatty acids including palmitic acid (C16:0) (467 mg, 1.82 mmol), arachidic acid (C20:0) (569 mg, 1.82 mmol), palmitoleic acid (C16:1) (463 mg, 1.82 mmol), or oleic acid (C18:1) (514 mg, 1.82 mmol) were incubated in 800 ml of Tris-HCl buffer (20 mM, pH 7.5) with 10% DME, which containing 100 mg purified SA_SCD. GM1βSph (d20:1) (1.57 g, 1.20 mmol) with palmitic acid (C16:0) (462 mg, 1.80 mmol), stearic acid (C18:0) (512 mg, 1.80 mmol), arachidic acid (C20:0) (563 mg, 1.80 mmol), palmitoleic acid (C16:1) (458 mg, 1.80 mmol) or oleic acid (C18:1) (508.5 mg, 1.80 mmol) were matched separately for catalytic synthesis of GM1 (d20:1) derivatives. The whole catalytic system was carried out at 37 ℃ for 6 h. TLC and UV-HPLC were used to monitor until the reaction was completed. When the best yield was obtained, the supernatant containing target compound was collected by centrifugation of 10000 rpm for 30 min at 4℃.

**7) Purification procedure for GM1 derivatives**

The supernatant was concentrated to a suitable volume and purified by C18 cartridges and silica gel filtration column. 5 column volumes of methanol/H_2_O (v/v) with gradients of 30% and 50% were used to remove impurities. Methanol with 75% and 80% gradient was used to retrieve GM1βSph (d18:1) and GM1βSph (d20:1) separately, which can be recycled for repeated reaction. Various GM1 derivatives and parts of fatty acids were eluted with 90% gradient methanol. After concentration by rotary evaporation, silica gel filtration column was used for further separation and purification.

5 column volumes of petroleum ether, 5 column volumes of petroleum ether/dichloromethane (v/v) and 5 column volumes of dichloromethane were used to remove stearic acid. Various pure GM1 derivatives were eluted by dichloromethane/methanol/H_2_O (70:28:4, v/v/v). The fractions containing pure product were collected and lyophilized to obtain various GM1 derivatives as a white powder. Their structures were identified by ^1^H and ^13^C NMR.

***β-D-Galactopyranosyl-(1→3)-(2-acetamido-2-deoxy-β-D-galactopyranosyl)-(1→4)-(5-acetamido-3,5-dideoxy-D-glycero-α-D-galacto-2-nonulopyranosylonic acid)-(2→3)-β-D-galactopyranosyl-(1→4)-β-D-glucopyranosyl-(1→1)-(2S, 3R, 4E)-2-octadecanamido-4-octadecene-1,3-diol (3)***

GM1 (d18:1/C18:0) **(3)**, white solid after lyophilization, 113.35 g, 71% yield. ^1^H NMR (700 MHz, MeOD) δ 5.68 (dt, *J* = 15.3, 7.0 Hz, 1H), 5.44 (dd, *J* = 15.5, 7.9 Hz, 1H), 4.92 (d, *J* = 8.7 Hz, 1H), 4.44 (d, *J* = 7.6 Hz, 1H), 4.41 (d, *J* = 7.9 Hz, 1H), 4.29 (d, *J* = 7.8 Hz, 1H), 4.20-3.26 (m, 33H), 2.73 (dd, *J* = 12.9, 4.8 Hz, 1H), 2.19 (t, *J* = 7.3 Hz, 2H), 2.01 (s, 3H), 1.99 (s, 3H), 1.91 (t, *J* = 12.1 Hz, 1H), 1.59-1.57 (m, 2H), 1.38-1.37 (m, 2H), 1.34-1.29 (m, 52H), 0.90 (t, *J* = 7.0 Hz, 6H).^13^C NMR (176 MHz, MeOD) δ 174.61, 174.37, 173.94, 173.53, 133.91, 129.78, 124.87, 105.06, 103.51, 103.00, 102.64, 102.02, 81.39, 79.86, 77.93, 77.75, 77.56, 75.07, 74.98, 74.93, 74.63, 74.50, 74.10, 73.71, 73.40, 73.16, 71.99, 71.61, 71.11, 69.64, 69.14, 68.90, 68.57, 68.31, 68.16, 64.14, 61.66, 61.15, 60.37, 60.24, 53.24, 52.39, 51.34, 37.10, 36.12, 32.16, 31.74, 29.49, 29.45, 29.42, 29.37, 29.31, 29.15, 29.09, 26.10, 25.85, 22.50, 22.42, 21.50, 13.32.

ESI HRMS(m/z): [M+Na]**^+^** calculated for C_73_H_131_N_3_NaO_31_, 1568.8659; found, 1568.8656.

***β-D-Galactopyranosyl-(1→3)-(2-acetamido-2-deoxy-β-D-galactopyranosyl)-(1→4)-(5-acetamido-3,5-dideoxy-D-glycero-α-D-galacto-2-nonulopyranosylonic acid)-(2→3)-β-D-galactopyranosyl-(1→4)-β-D-glucopyranosyl-(1→1)-(2S, 3R, 4E)-2-hexadecanamino-4-octadecene-1,3-diol(19)***

GM1 (d18:1/C16:0) **(19)**, white solid after lyophilization, 1.38 g, 73% yield. ^1^H NMR (700 MHz, MeOD) δ 5.72 (dt, *J* = 15.2, 7.0 Hz, 1H), 5.48 (dd, *J* = 15.2, 7.8 Hz, 1H), 4.94 (d, *J* = 8.6 Hz, 1H), 4.48 (d, *J* = 7.6 Hz, 1H), 4.46 (d, *J* = 8.0 Hz, 1H), 4.33 (d, *J* = 8.0 Hz, 1H), 4.24-3.33 (m, 33H), 2.76 (dd, *J* = 12.8, 4.8 Hz, 1H), 2.21 (t, *J* = 7.6 Hz, 2H), 2.06 (s, 3H), 2.03 (s, 3H), 1.94 (t, *J* = 12.3 Hz, 1H), 1.62-1.61 (m, 2H), 1.41-1.39 (m, 2H), 1.36-1.31 (m, 48H), 0.93 (t, *J* = 7.1 Hz, 6H).^13^C NMR (176 MHz, MeOD) δ 174.61, 174.37, 173.94, 173.53, 133.91, 129.78, 124.87, 105.06, 103.51, 103.00, 102.64, 102.02, 81.39, 79.86, 77.93, 77.75, 77.56, 75.07, 74.98, 74.93, 74.63, 74.50, 74.10, 73.71, 73.40, 73.16, 71.99, 71.61, 71.11, 69.64, 69.14, 68.90, 68.57, 68.31, 68.16, 64.14, 61.66, 61.15, 60.37, 60.24, 53.24, 52.39, 51.34, 37.10, 36.12, 32.16, 31.74, 29.49, 29.45, 29.42, 29.37, 29.31, 29.15, 29.09, 26.10, 25.85, 22.50, 22.42, 21.50, 13.32.

ESI HRMS(m/z): [M+Na]**^+^** calculated for C_71_H_127_N_3_NaO_31_, 1540.8346; found, 1540.8366.

***β-D-Galactopyranosyl-(1→3)-(2-acetamido-2-deoxy-β-D-galactopyranosyl)-(1→4)-(5-acetamido-3,5-dideoxy-D-glycero-α-D-galacto-2-nonulopyranosylonic acid)-(2→3)-β-D-galactopyranosyl-(1→4)-β-D-glucopyranosyl-(1→1)-(2S, 3R, 4E)-2-eicosamido-4-octadecene-1,3-diol (20)***

GM1 (d18:1/C20:0) **(20)**, white solid after lyophilization, 1.29 g, 66% yield. ^1^H NMR (700 MHz, MeOD) δ 5.64 (dt, *J* = 15.3, 7.0 Hz, 1H), 5.40 (dd, *J* = 15.5, 7.9 Hz, 1H), 4.87 (d, *J* = 8.7 Hz, 1H), 4.40 (d, *J* = 7.6 Hz, 1H), 4.37 (d, *J* = 7.8 Hz, 1H), 4.25 (d, *J* = 7.8 Hz, 1H), 4.15-3.22 (m, 33H), 2.69 (dd, *J* = 13.1, 4.8 Hz, 1H), 2.13 (t, *J* = 7.5 Hz, 2H), 1.97 (s, 3H), 1.95 (s, 3H), 1.91 (t, *J* = 12.0 Hz, 1H), 1.55-1.53 (m, 2H), 1.34-1.33 (m, 2H), 1.34-1.29 (m, 52H), 0.86 (t, *J* = 6.9 Hz, 6H).^13^C NMR (176 MHz, MeOD) δ 181.24, 174.56, 174.27, 173.85, 173.46, 133.68, 129.99, 105.25, 103.54, 103.05, 102.73, 102.10, 81.61, 79.87, 77.63, 75.08, 75.03, 74.94, 74.71, 74.51, 74.21, 73.70, 73.44, 73.17, 71.99, 71.55, 71.10, 69.66, 69.05, 68.83, 68.50, 68.34, 68.30, 63.99, 63.00, 61.59, 60.99, 60.38, 60.31, 56.07, 53.29, 52.37, 51.33, 48.13, 37.52, 37.13, 35.97, 32.08, 31.70, 31.68, 29.49, 29.46, 29.42, 29.39, 29.31, 29.25, 29.12, 29.08, 29.03, 26.27, 25.78, 22.36, 22.34, 21.18, 13.07.

ESI HRMS(m/z): [M+Na]**^+^** calculated for C_75_H_135_N_3_NaO_31_, 1596.8972; found, 1596.8953.

***β-D-Galactopyranosyl-(1→3)-(2-acetamido-2-deoxy-β-D-galactopyranosyl)-(1→4)-(5-acetamido-3,5-dideoxy-D-glycero-α-D-galacto-2-nonulopyranosylonic acid)-(2→3)-β-D-galactopyranosyl-(1→4)-β-D-glucopyranosyl-(1→1)-(2S, 3R, 4E)-2-hexadec-9-enamide-4-octadecene-1,3-diol (21)***

GM1 (d18:1/C16:1) **(21)**, white solid after lyophilization, 1.54 g, 82% yield. ^1^H NMR (700 MHz, MeOD) δ 5.59 (dt, *J* = 15.2, 7.1 Hz, 1H), 5.36 (dd, *J* = 15.5, 7.8 Hz, 1H), 5.28-5.23(m, 2H), 4.83 (d, *J* = 8.6 Hz, 1H), 4.36 (d, *J* = 7.6 Hz, 1H), 4.33 (d, *J* = 7.9 Hz, 1H), 4.21 (d, *J* = 7.6 Hz, 1H), 4.10-3.18 (m, 33H), 2.64 (dd, *J* = 12.7, 4.8 Hz, 1H), 2.08 (t, *J* = 7.6 Hz, 2H), 1.95-1.90 (m, 12H), 1.82 (t, *J* = 12.3 Hz, 1H), 1.50-1.49 (m, 2H), 1.29-1.20 (m, 40H), 0.82-0.80 (m, 6H, 2×CH_3_).^13^C NMR (176 MHz, MeOD) δ 174.54, 174.28, 173.92, 173.45, 133.67, 129.98, 129.51, 129.39, 105.24, 103.54, 103.03, 102.67, 102.07, 81.57, 79.87, 77.60, 75.07, 75.02, 74.93, 74.70, 74.50, 74.21, 73.70, 73.44, 73.18, 71.99, 71.57, 71.10, 69.65, 69.03, 68.83, 68.51, 68.33, 68.28, 63.96, 61.59, 60.99, 60.37, 60.34, 53.28, 52.37, 51.38, 48.14, 37.13, 35.96, 32.07, 31.70, 31.56, 29.52, 29.48, 29.45, 29.41 29.37, 29.13, 29.11, 29.06, 29.05, 28.91, 28.68, 26.78, 25.78, 22.38, 22.36, 22.35, 21.20, 13.09, 13.08.

ESI HRMS(m/z): [M+Na]**^+^** calculated for C_71_H_125_N_3_NaO_31_, 1538.8189; found, 1538.8557.

***β-D-Galactopyranosyl-(1→3)-(2-acetamido-2-deoxy-β-D-galactopyranosyl)-(1→4)-(5-acetamido-3,5-dideoxy-D-glycero-α-D-galacto-2-nonulopyranosylonic acid)-(2→3)-β-D-galactopyranosyl-(1→4)-β-D-glucopyranosyl-(1→1)-(2S, 3R, 4E)-2-octadec-9-enamide-4-octadecene-1,3-diol (22)***

GM1 (d18:1/C18:1) **(22)**, white solid after lyophilization, 1.51 g, 79% yield. ^1^H NMR (700 MHz, MeOD) δ 5.60 (dt, *J* = 15.3, 7.2 Hz, 1H), 5.37 (dd, *J* = 15.3, 8.0 Hz, 1H), 5.28-5.23 (m, 2H), 4.83 (1H), 4.36 (d, *J* = 7.7 Hz, 1H), 4.33 (d, *J* = 7.8 Hz, 1H), 4.22 (d, *J* = 7.8 Hz, 1H), 4.10-3.18 (m, 33H), 2.64 (dd, *J* = 12.7, 4.8 Hz, 1H), 2.08 (t, *J* = 7.6 Hz, 2H), 1.94-1.90 (m, 12H), 1.82 (t, *J* = 12.1 Hz, 1H), 1.50-1.49 (m, 2H), 1.29-1.20(m, 44H), 0.82-0.80 (m, 6H, 2×CH_3_).^13^C NMR (176 MHz, MeOD) δ 174.53, 174.28, 173.93, 173.45, 133.67, 129.99, 129.51, 129.39, 105.23, 103.54, 103.03, 102.65, 102.07, 81.56, 79.86, 77.59, 75.07, 75.02, 74.93, 74.70, 74.50, 74.21, 73.70, 73.43, 73.18, 71.99, 71.57, 71.10, 69.65, 69.02, 68.83, 68.52, 68.33, 68.28, 63.95, 61.58, 60.99, 60.37, 60.34, 53.28, 52.37, 51.39, 48.14, 48.02, 47.89, 37.13, 35.96, 32.07, 31.70, 31.57, 29.53, 29.48, 29.46, 29.43, 29.42, 29.38, 29.14, 29.11, 29.07, 29.06, 29.03, 28.92, 28.68, 26.80, 25.78, 22.38, 22.36, 21.21, 13.11, 13.09.

ESI HRMS(m/z): [M+Na]**^+^** calculated for C_73_H_129_N_3_NaO_31_, 1566.8502; found, 1566.8736.

***β-D-Galactopyranosyl-(1→3)-(2-acetamido-2-deoxy-β-D-galactopyranosyl)-(1→4)-(5-acetamido-3,5-dideoxy-D-glycero-α-D-galacto-2-nonulopyranosylonic acid)-(2→3)-β-D-galactopyranosyl-(1→4)-β-D-glucopyranosyl-(1→1)-(2S, 3R, 4E)-2-hexadecanamino-4-eicosene-1,3-diol (23)***

GM1 (d20:1/C16:0) **(23)**, white solid after lyophilization, 1.32 g, 70% yield. ^1^H NMR (700 MHz, MeOD) δ 5.70 (dt, *J* = 15.3, 7.0 Hz, 1H), 5.46 (dd, *J* = 15.3, 7.8 Hz, 1H), 4.93 (d, *J* = 8.7 Hz, 1H), 4.46 (d, *J* = 7.8 Hz, 1H), 4.43 (d, *J* = 7.8 Hz, 1H), 4.31 (d, *J* = 7.8 Hz, 1H), 4.20-3.28 (m, 33H), 2.75 (dd, *J* = 12.8, 4.8 Hz, 1H), 2.19 (t, *J* = 7.3 Hz, 2H), 2.03 (s, 3H), 2.01 (s, 3H), 1.92 (t, *J* = 12.3 Hz, 1H), 1.62-1.58 (m, 2H), 1.42-1.38 (m, 2H), 1.36-1.30 (m, 52H), 0.92 (t, *J* = 7.1 Hz, 6H).^13^C NMR (176 MHz, MeOD) δ 178.96, 174.57, 174.27, 173.84, 173.45, 133.67, 129.98, 129.45, 105.22, 104.84, 103.54, 103.05, 102.72, 102.08, 81.60, 79.82, 77.64, 75.34, 75.09, 75.05, 74.94, 74.70, 74.51, 74.20, 73.70, 73.44, 73.19, 72.45, 71.99, 71.58, 71.10, 69.65, 69.05, 68.85, 68.55, 68.34, 68.29, 63.97, 63.42, 63.01, 61.58, 61.00, 60.38, 60.35, 53.31, 52.39, 51.32, 37.16, 35.98, 35.14, 32.08, 31.70, 31.68, 29.49, 29.45, 29.42, 29.41, 29.37, 29.34, 29.33, 29.31, 29.24, 29.20, 29.11, 29.07, 29.03, 28.92, 26.71, 25.78, 25.52, 22.83, 22.37, 22.36, 22.35, 21.19, 13.07.

ESI HRMS(m/z): [M+Na]**^+^** calculated for C_73_H_131_N_3_NaO_31_, 1568.8659; found, 1568.8664.

***β-D-Galactopyranosyl-(1→3)-(2-acetamido-2-deoxy-β-D-galactopyranosyl)-(1→4)-(5-acetamido-3,5-dideoxy-D-glycero-α-D-galacto-2-nonulopyranosylonic acid)-(2→3)-β-D-galactopyranosyl-(1→4)-β-D-glucopyranosyl-(1→1)-(2S, 3R, 4E)-2-octadecanamido-4-eicosene-1,3-diol (24)***

GM1 (d20:1/C18:0) **(24)**, white solid after lyophilization, 1.33 g, 69% yield. ^1^H NMR (700 MHz, MeOD) δ 5.68 (dt, *J* = 15.3, 7.0 Hz, 1H), 5.44 (dd, *J* = 15.5, 7.9 Hz, 1H), 4.92 (d, *J* = 8.7 Hz, 1H), 4.44 (d, *J* = 7.6 Hz, 1H), 4.41 (d, *J* = 7.9 Hz, 1H), 4.29 (d, *J* = 7.8 Hz, 1H), 4.20-3.26 (m, 33H), 2.73 (dd, *J* = 12.9, 4.8 Hz, 1H), 2.19 (t, *J* = 7.3 Hz, 2H), 2.01 (s, 3H), 1.99 (s, 3H), 1.91 (t, *J* = 12.1 Hz, 1H), 1.59-1.57 (m, 2H), 1.38-1.37 (m, 2H), 1.34-1.29 (m, 52H), 0.90 (t, *J* = 7.0 Hz, 6H).^13^C NMR (176 MHz, MeOD) δ 174.61, 174.37, 173.94, 173.53, 133.91, 129.78, 124.87, 105.06, 103.51, 103.00, 102.64, 102.02, 81.39, 79.86, 77.93, 77.75, 77.56, 75.07, 74.98, 74.93, 74.63, 74.50, 74.10, 73.71, 73.40, 73.16, 71.99, 71.61, 71.11, 69.64, 69.14, 68.90, 68.57, 68.31, 68.16, 64.14, 61.66, 61.15, 60.37, 60.24, 53.24, 52.39, 51.34, 37.10, 36.12, 32.16, 31.74, 29.49, 29.45, 29.42, 29.37, 29.31, 29.15, 29.09, 26.10, 25.85, 22.50, 22.42, 21.50, 13.32.

ESI HRMS(m/z): [M+Na]**^+^** calculated for C_75_H_135_N_3_NaO_31_, 1596.8972; found, 1596.9004.

***β-D-Galactopyranosyl-(1→3)-(2-acetamido-2-deoxy-β-D-galactopyranosyl)-(1→4)-(5-acetamido-3,5-dideoxy-D-glycero-α-D-galacto-2-nonulopyranosylonic acid)-(2→3)-β-D-galactopyranosyl-(1→4)-β-D-glucopyranosyl-(1→1)-(2S, 3R, 4E)-2-eicosamido-4-eicosene-1,3-diol (25)***

GM1 (d20:1/C20:0) **(25)**, white solid after lyophilization, 1.25 g, 64% yield. ^1^H NMR (700 MHz, MeOD) δ 5.66 (dt, *J* = 15.3, 7.0 Hz, 1H), 5.42 (dd, *J* = 15.6, 7.9 Hz, 1H), 4.90 (d, *J* = 9.0 Hz, 1H), 4.38 (d, *J* = 7.8 Hz, 1H), 4.26 (d, *J* = 7.8 Hz, 1H), 4.13-3.24 (m, 33H), 2.71 (dd, *J* = 12.6, 4.8 Hz, 1H), 2.14 (t, *J* = 7.5 Hz, 2H), 1.97 (s, 3H), 1.96 (s, 3H), 1.84 (t, *J* = 12.2 Hz, 1H), 1.58-1.54 (m, 2H), 1.37-1.33 (m, 2H), 1.33-1.25 (m, 56H), 0.85 (t, *J* = 7.0 Hz, 6H).^13^C NMR (176 MHz, MeOD) δ 176.56, 176.30, 175.88, 175.48, 135.81, 131.87, 131.49, 107.14, 105.55, 105.01, 104.67, 104.05, 83.52, 81.94, 79.97, 79.78, 79.59, 77.07, 76.99, 76.94, 76.66, 76.51, 76.14, 75.70, 75.41, 75.16, 73.97, 73.58, 73.11, 71.63, 71.14, 70.87, 70.53, 70.33, 70.21, 66.12, 63.64, 63.09, 62.40, 62.23, 55.25, 54.38, 53.33, 50.31, 39.12, 38.07, 37.23, 35.08, 34.13, 33.72, 31.51, 31.46, 31.43, 31.35, 31.29, 31.13, 31.07, 30.97, 28.78, 28.19, 27.82, 27.55, 25.05, 24.44, 24.38, 23.37, 20.01, 19.89, 15.22.

ESI HRMS(m/z): [M+Na]**^+^** calculated for C_77_H_139_N_3_NaO_31_, 1624.9285; found, 1624.9291.

***β-D-Galactopyranosyl-(1→3)-(2-acetamido-2-deoxy-β-D-galactopyranosyl)-(1→4)-(5-acetamido-3,5-dideoxy-D-glycero-α-D-galacto-2-nonulopyranosylonic acid)-(2→3)-β-D-galactopyranosyl-(1→4)-β-D-glucopyranosyl-(1→1)-(2S, 3R, 4E)-2-hexadec-9-enamide-4-eicosene-1,3-diol (26)***

GM1 (d20:1/C16:1) **(26)**, white solid after lyophilization, 1.49 g, 79% yield. ^1^H NMR (700 MHz, MeOD) δ 5.68 (dt, *J* = 15.5, 7.0 Hz, 1H), 5.44 (dd, *J* = 15.6, 7.8 Hz, 1H), 5.36-5.32 (m, 2H), 4.91 (d, *J* = 8.6 Hz, 1H), 4.44 (d, *J* = 8.0 Hz, 1H), 4.42 (d, *J* = 8.0 Hz, 1H), 4.30 (d, *J* = 8.0 Hz, 1H), 4.19-3.26 (m, 33H), 2.73 (dd, *J* = 12.8, 4.8 Hz, 1H), 2.17 (t, *J* = 7.4 Hz, 2H), 2.03-1.99 (m, 12H), 1.91 (t, *J* = 12.0 Hz, 1H), 1.58-1.57 (m, 2H), 1.35-1.28 (m, 44H), 0.91-0.89 (m, 6H, 2×CH_3_).^13^C NMR (176 MHz, MeOD) δ 174.54, 174.28, 173.92, 173.45, 133.67, 129.98, 129.51, 129.39, 105.24, 103.54, 103.03, 102.67, 102.07, 81.57, 79.87, 77.60, 75.07, 75.02, 74.93, 74.70, 74.50, 74.21, 73.70, 73.44, 73.18, 71.99, 71.57, 71.10, 69.65, 69.03, 68.83, 68.51, 68.33, 68.28, 63.96, 61.59, 60.99, 60.37, 60.34, 53.28, 52.37, 51.38, 48.14, 48.02, 47.90, 37.13, 35.96, 32.07, 31.70, 31.56, 29.52, 29.48, 29.45, 29.41, 29.37, 29.13, 29.11, 29.06, 29.05, 29.02, 28.91, 28.68, 26.79, 25.78, 22.38, 22.36, 22.35, 21.20, 13.09, 13.08.

ESI HRMS(m/z): [M+Na]**^+^** calculated for C_73_H_129_N_3_NaO_31_, 1566.8502; found, 1566.8504.

***β-D-Galactopyranosyl-(1→3)-(2-acetamido-2-deoxy-β-D-galactopyranosyl)-(1→4)-(5-acetamido-3,5-dideoxy-D-glycero-α-D-galacto-2-nonulopyranosylonic acid)-(2→3)-β-Dgalactopyranosyl-(1→4)-β-D-glucopyranosyl-(1→1)-(2S, 3R, 4E)-2-octadec-9-enamide-4-eicosene-1,3-diol (27)***

GM1 (d20:1/C18:1) **(27)**, white solid after lyophilization, 1.46 g, 76% yield. ^1^H NMR (700 MHz, MeOD) δ 5.68 (dt, *J* = 15.2, 7.2 Hz, 1H), 5.44 (dd, *J* = 15.2, 7.8 Hz, 1H), 5.36-5.32 (m, 2H), 4.92 (1H), 4.45 (d, *J* = 7.8 Hz, 1H), 4.42 (d, *J* = 7.8 Hz, 1H), 4.30 (d, *J* = 7.8 Hz, 1H), 4.19-3.26 (m, 33H), 2.73 (dd, *J* = 12.5, 4.9 Hz, 1H), 2.17 (t, *J* = 7.5 Hz, 2H), 2.03-1.99 (m, 12H), 1.91 (t, *J* = 12.1 Hz, 1H), 1.58-1.57 (m, 2H), 1.32-1.29(m, 48H), 0.91-0.89 (m, 6H, 2×CH_3_).^13^C NMR (176 MHz, MeOD) δ 174.52, 174.28, 173.89, 173.46, 133.68, 130.00, 129.50, 129.46, 129.40, 105.23, 103.53, 103.03, 102.69, 102.08, 81.58, 79.82, 77.62, 75.08, 75.02, 74.93, 74.69, 74.50, 74.20, 73.69, 73.43, 73.17, 71.99, 71.56, 71.09, 69.65, 69.02, 68.83, 68.53, 68.32, 68.28, 63.94, 61.58, 61.00, 60.34, 53.27, 52.37, 51.35, 48.15, 48.02, 47.90, 37.13, 35.97, 32.08, 31.70, 29.54, 29.49, 29.47, 29.44, 29.39, 29.24, 29.15, 29.11, 29.09, 29.08, 29.03, 28.98, 28.93, 26.81, 26.78, 25.79, 22.37, 21.22, 13.11, 13.09.

ESI HRMS(m/z): [M+Na]**^+^** calculated for C_75_H_133_N_3_NaO_31_, 1594.8815; found, 1594.8838.

**8) The cost accounting for GM1 synthesis at hectogram scale**

The cost estimation for the preparation of 100 gram of GM1 through MOCECA strategy was conducted. The costs of various projects required in GM1 preparation were listed. (1) Total cost of main substrates and reagents was 2013.70 USD (Average cost was 20.14 USD/g), detailed expenses were listed in Table 1. (2) Total cost of various enzymes was 37241.93 USD (Average cost was 372.42 USD/g), including NmCSS (1.38g, 484.40 USD), PmST1(1.32g, 91.30 USD), PmPpA (1.34g, 12.17 USD), BLNahK (1.06g, 38.92 USD), AGX1 (0.79g, 71.66 USD), EcGalK (1.59g, 69.27 USD), AtUSP (1.38g, 50.15 USD), CjCgtA (2.65g, 547.83 USD), CjCgtB (3.18g, 365.22 USD), EGC-II E351S/D314Y (21.17g, 2641.26 USD), SA_SCD (8.82g, 32869.75 USD). (3) Labor cost was 5519.68 USD (Average cost was 55.20 USD/g). Purification cost was 2434.79 USD (24.35 USD/g). The cost to synthesize 100 grams of GM1 in total was 47210.10 USD (Average cost was 472.10 USD/g). Further engineering studies based on this strategy, such as immobilization of the enzymes and optimization of the purification steps, are feasible for greatly reducing the cost of the entire process. It enabled MOCECA believed to provide a cost-effective means for the large scale preparation of ganglioside in the future.

Supplementary Table 1. The cost accounting for preparation of 100 grams of GM1

| Projects required in GM1 preparation | Cost per 100 gram | Average cost |
| --- | --- | --- |
| **(1) Main substrates and reagents** |  |  |
| Lac-F (36.42 g) | 389.56 USD | 3.90 USD/g |
| Neu5Ac (55.62 g) | 77.92 USD | 0.78 USD/g |
| CTP (89.27 g) | 77.94 USD | 0.78 USD/g |
| GalNAc (28.08 g) | 150.90 USD | 1.51 USD/g |
| ATP (151.58 g) | 38.96 USD | 0.39 USD/g |
| UTP (145.31 g) | 160.70 USD | 1.61 USD/g |
| Gal (20.98 g) | 2.8 USD | 0.03 USD/g |
| L-serine (60.42 g) | 3.34 USD | 0.03 USD/g |
| Acetyl chloride (302.11 mL) | 3.13 USD | 0.03 USD/g |
| (Boc)_2_O (141.39 g) | 2.63 USD | 0.03 USD/g |
| Triethylamine (181.27 mL) | 1.67 USD | 0.02 USD/g |
| TBDMSCl (141.99 g) | 3.63 USD | 0.04 USD/g |
| Imidazole (64.65 g) | 1.67 USD | 0.02 USD/g |
| Dimethyl methyl phosphonate (240.18 g) | 21.68 USD | 0.22 USD/g |
| *n*-BuLi (679.76 mL, 2.5 M in hexane) | 37.52 USD | 0.38 USD/g |
| LiCl (36.56 g) | 3.34 USD | 0.03 USD/g |
| *n*-tetradecanal (93.66 g) | 29.18 USD | 0.29 USD/g |
| LiAlH(O*^t^*Bu)_3_ (230.21 g) | 91.72 USD | 0.92 USD/g |
| Dichloromethane (6.04 L) | 60.70 USD | 0.61 USD/g |
| Petroleum Ether (7.55 L) | 19.60 USD | 0.20 USD/g |
| Ethyl Acetate (3.02 L) | 10.84 USD | 0.11 USD/g |
| Methanol (6.04 L) | 22.51 USD | 0.23 USD/g |
| Stearic acid (C18:0) (38.31 g) | 570.94 USD | 5.71 USD/g |
| DME (4.41 L) | 230.82 USD | 2.31 USD/g |
| Total cost of main substrates and reagents | 2013.70 USD | 20.14 USD/g |
| **(2) Various enzymes** |  |  |
| NmCSS (1.38 g) | 484.40 USD | 4.84 USD/g |
| PmST1(1.32 g) | 91.30 USD | 0.91 USD/g |
| PmPpA (1.34 g) | 12.17 USD | 0.12 USD/g |
| BLNahK (1.06 g) | 38.92 USD | 0.39 USD/g |
| AGX1 (0.79 g) | 71.66 USD | 0.72 USD/g |
| EcGalK (1.59 g) | 69.27 USD | 0.69 USD/g |
| AtUSP (1.38 g) | 50.15 USD | 0.50 USD/g |
| CjCgtA (2.65 g) | 547.83 USD | 5.48 USD/g |
| CjCgtB (3.18 g) | 365.22 USD | 3.65 USD/g |
| EGC-II E351S/D314Y (21.17 g) | 2641.26 USD | 26.41 USD/g |
| SA_SCD (8.82 g) | 32869.75 USD | 328.70 USD/g |
| Total cost of various enzymes | 37241.93 USD | 372.42 USD/g |
| **(3) Labor cost** | 5519.68 USD | 55.20 USD/g |
| **(4) Purification cost** | 2434.79 USD | 24.35 USD/g |
| **In total** | 47210.10 USD | 472.10 USD/g |

**Ⅲ. HRMS spectrum of compounds in modular chemoenzymatic cascade strategy**

1. HRMS spectrum of GM3βSph, GM2βSph, GM1βSph, GD3βSph, GD2βSph

1. HRMS spectrum of GM3βSph

ESI HRMS(m/z): [M+H]**^+^** calculated for C_41_H_75_N_2_O_20_, 915.4908; found, 915.4920.

ESI HRMS(m/z): [M+Na]**^+^** calculated for C_41_H_74_N_2_NaO_20_, 937.4727; found, 937.4721.


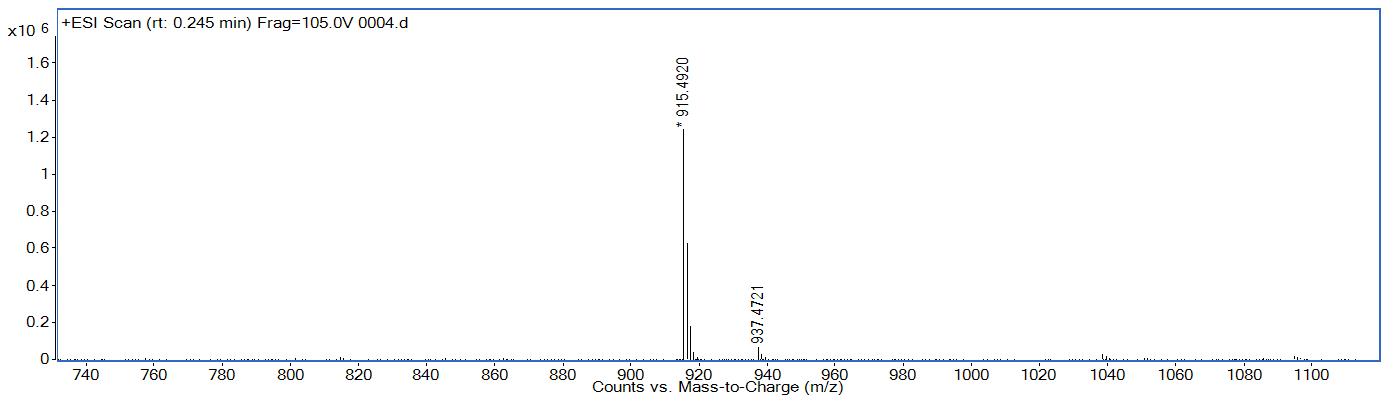


1. HRMS spectrum of GM2βSph

ESI HRMS(m/z): [M+H]**^+^** calculated for C_49_H_88_N_3_O_25_, 1118.5701; found, 1118.5750.


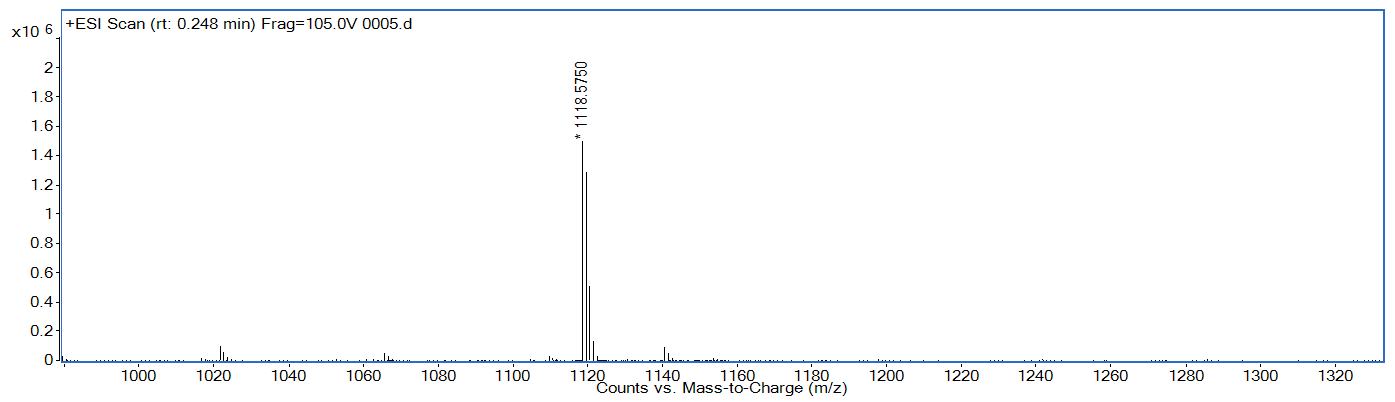


1. HRMS spectrum of GM1βSph

ESI HRMS(m/z): [M+H]**^+^** calculated for C_55_H_98_N_3_O_30_, 1280.6230; found, 1280.6266.

ESI HRMS(m/z): [M+Na]**^+^** calculated for C_55_H_97_N_3_NaO_30_, 1302.6049; found, 1302.6025.


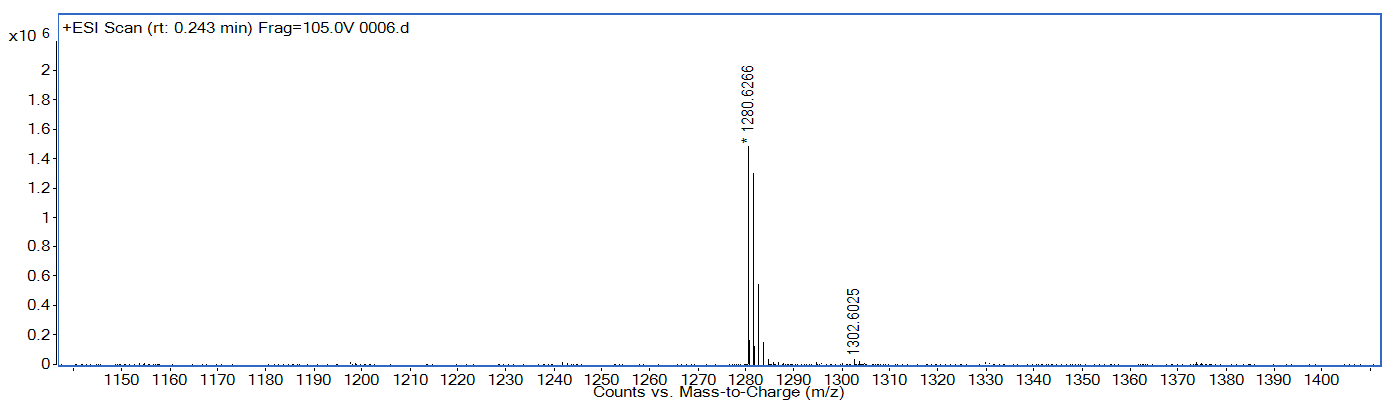


1. HRMS spectrum of GD3βSph

ESI HRMS(m/z): [M+H]**^+^** calculated for C_52_H_92_N_3_O_28_, 1206.5862; found, 1206.5897.


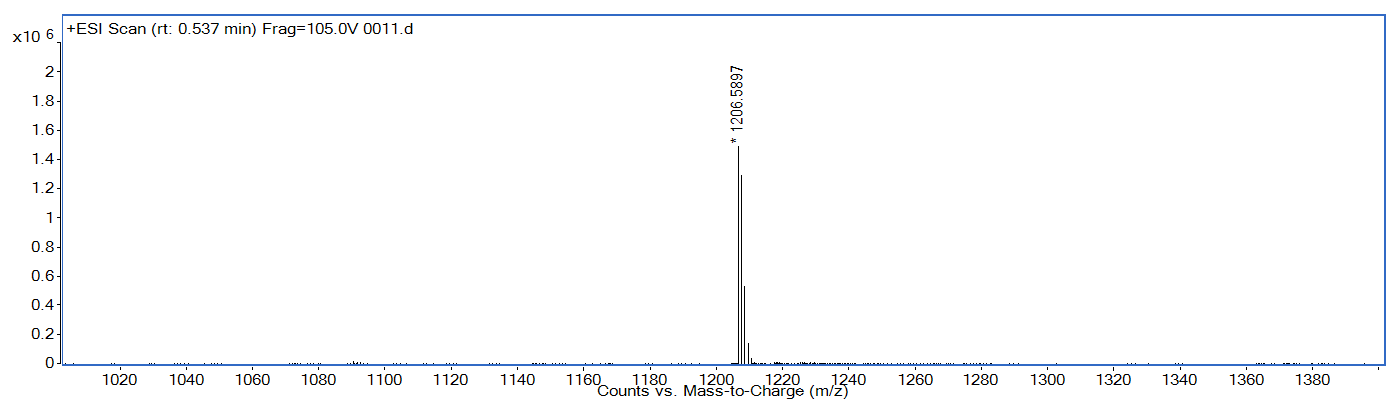


1. HRMS spectrum of GD2βSph

ESI HRMS(m/z): [M+H]**^+^** calculated for C_60_H_105_N_4_O_33_, 1409.6656; found, 1409.6693.


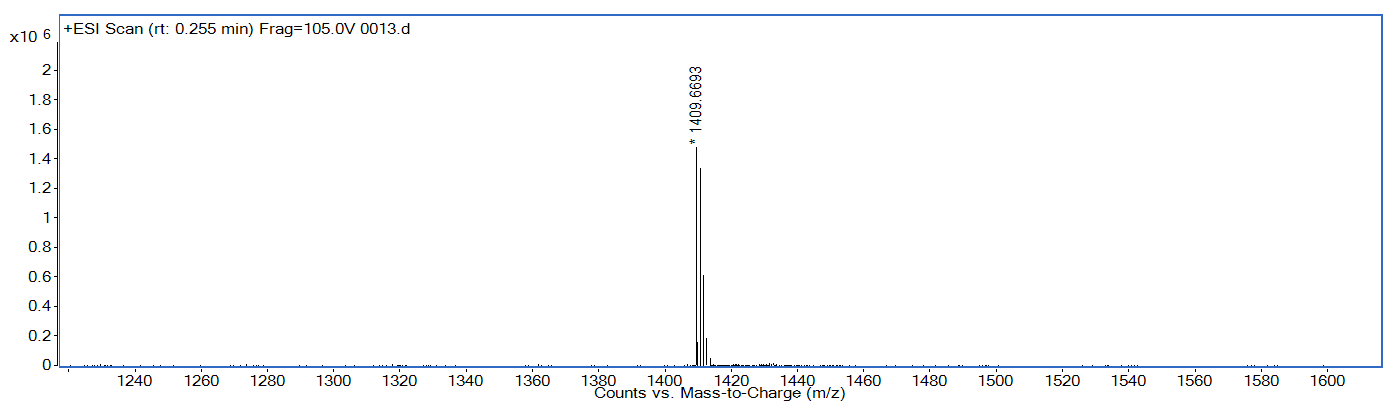


2. HRMS spectrum of ganglioside including GM3, GM2, GM1, GD3, GD2

1. HRMS spectrum of GM3

ESI HRMS(m/z): [M+H]**^+^** calculated for C_59_H_109_N_2_O_21_, 1181.7517; found, 1181.7485.

ESI HRMS(m/z): [M+Na]**^+^** calculated for C_59_H_108_N_2_NaO_21_, 1203.7337; found, 1203.7294.


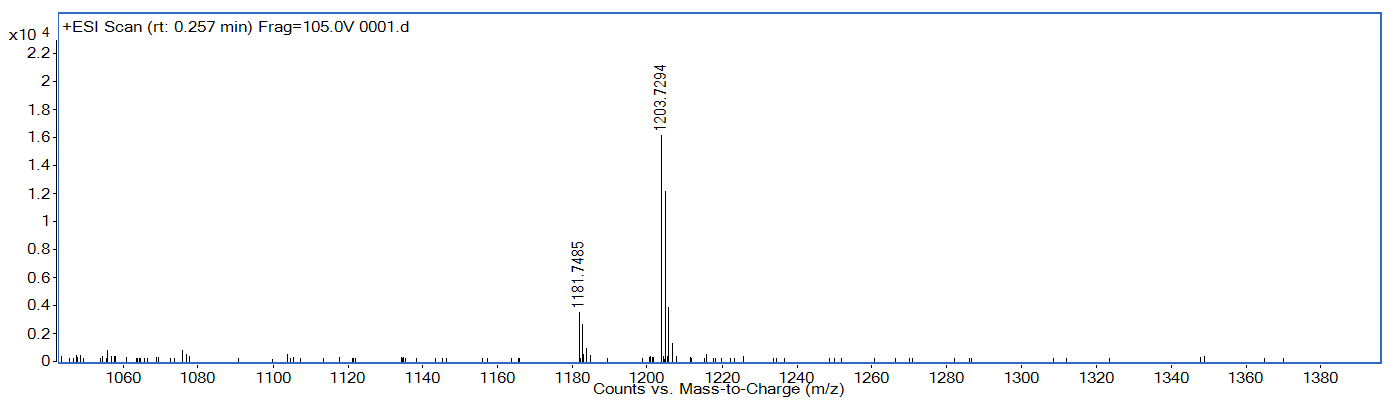


1. HRMS spectrum of GM2

ESI HRMS(m/z): [M+H]**^+^** calculated for C_67_H_122_N_3_O_26_, 1384.8311; found, 1384.8326.

ESI HRMS(m/z): [M+Na]**^+^** calculated for C_67_H_121_N_3_NaO_26_, 1406.8131; found, 1406.8158.


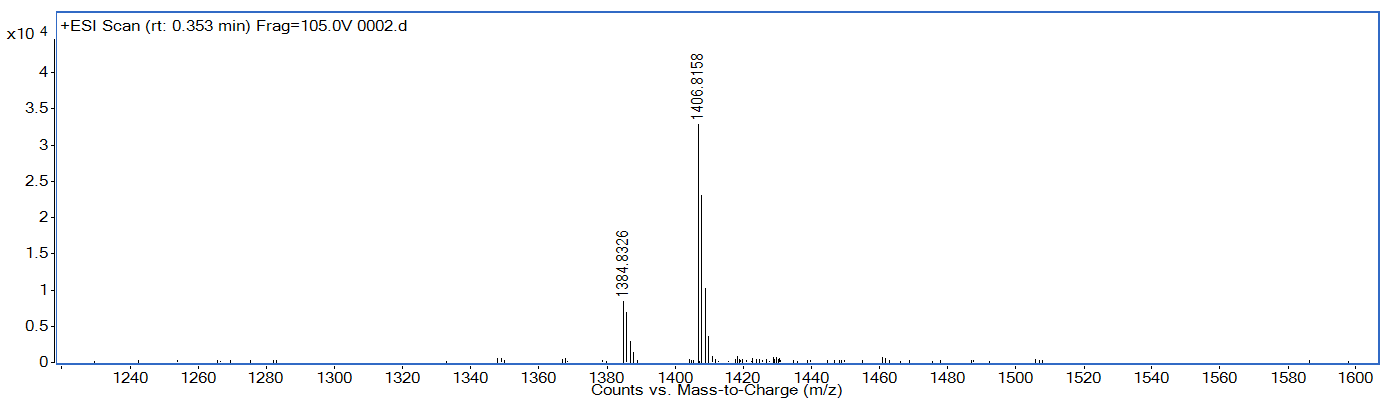


1. HRMS spectrum of GM1

ESI HRMS(m/z): [M+H]**^+^** calculated for C_73_H_132_N_3_O_31_, 1546.8839; found, 1546.8754.

ESI HRMS(m/z): [M+Na]**^+^** calculated for C_73_H_131_N_3_NaO_31_, 1568.8659; found, 1568.8582.


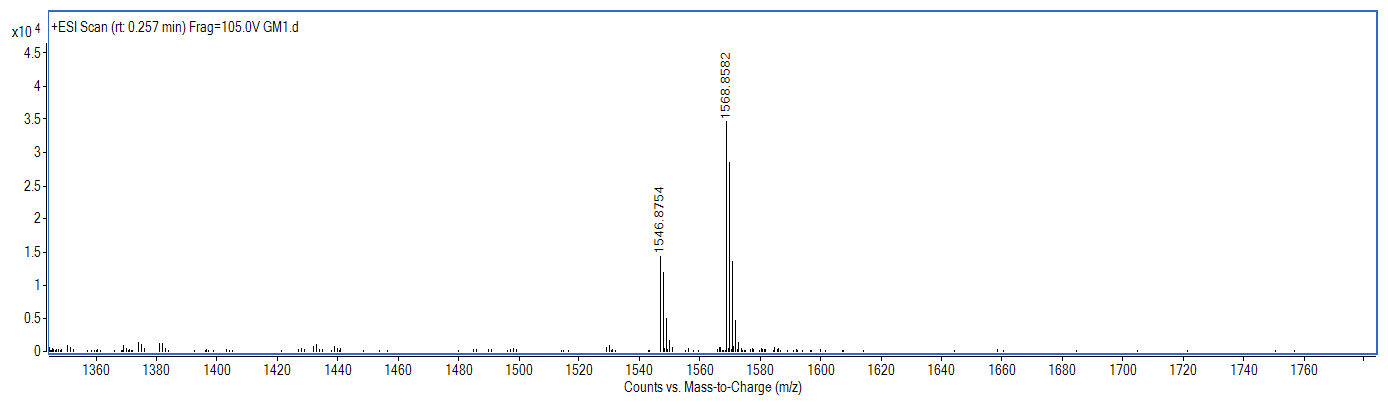


1. HRMS spectrum of GD3

ESI HRMS(m/z): [M+Na]**^+^** calculated for C_70_H_125_N_3_NaO_29_, 1494.8291; found, 1494.8237.


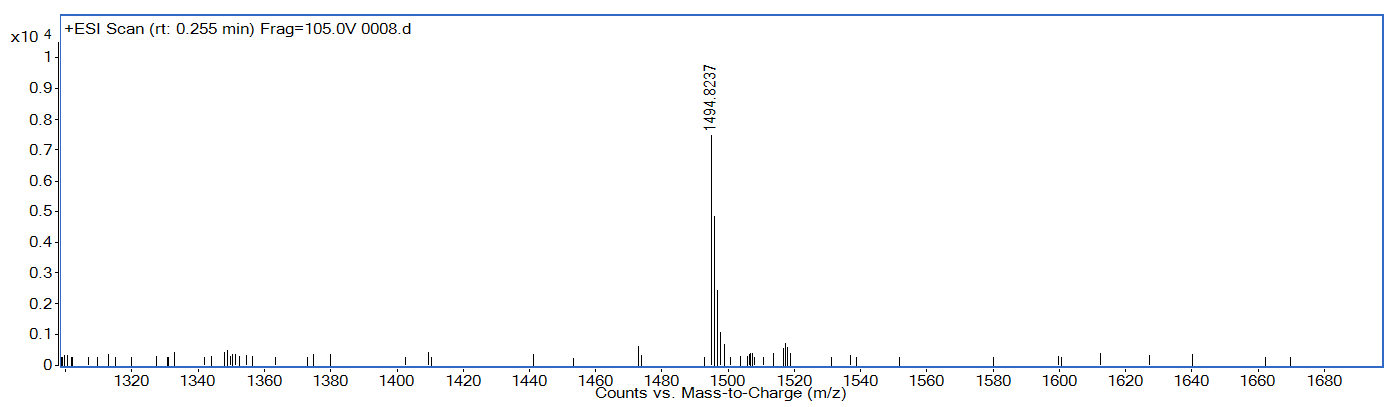


1. HRMS spectrum of GD2

ESI HRMS(m/z): [M+H]**^+^** calculated for C_78_H_140_N_4_O_34_, 1676.9343; found, 1676.9353.

ESI HRMS(m/z): [M+Na]**^+^** calculated for C_78_H_139_N_4_NaO_34_, 1698.9163; found, 1698.8984.


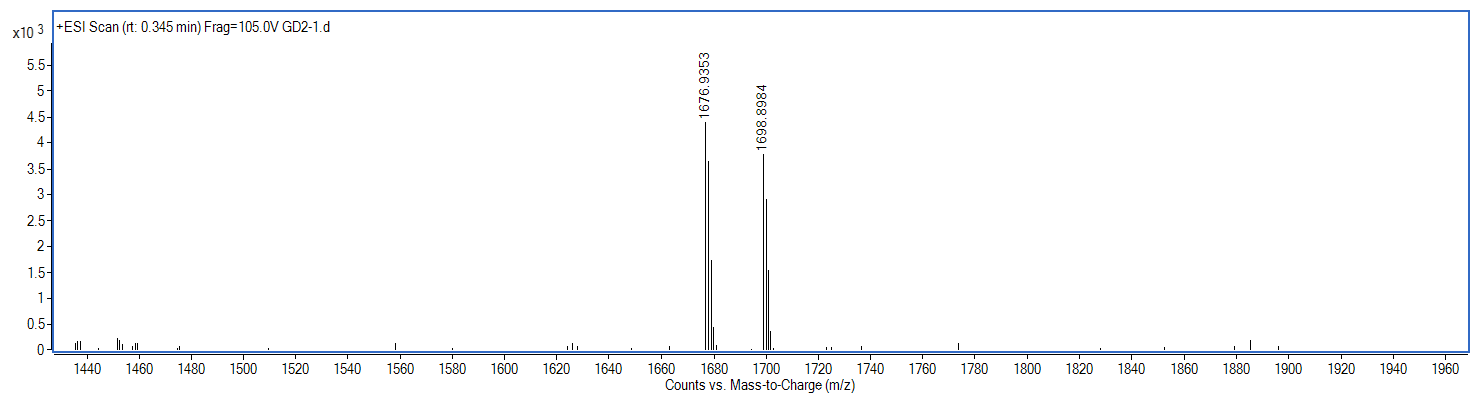


**3. HRMS spectrum of ganglioside GM1 derivatives**

1. HRMS spectrum of GM1 (d18:1/C16:0)

ESI HRMS(m/z): [M+H]**^+^** calculated for C_71_H_128_N_3_O_31_, 1518.8526; found, 1518.8531.

ESI HRMS(m/z): [M+Na]**^+^** calculated for C_71_H_127_N_3_NaO_31_, 1540.8346; found, 1540.8366.

1. HRMS spectrum of GM1 (d18:1/C18:0)

ESI HRMS(m/z): [M+H]**^+^** calculated for C_73_H_132_N_3_O_31_, 1546.8839; found, 1546.8841.

ESI HRMS(m/z): [M+Na]**^+^** calculated for C_73_H_131_N_3_NaO_31_, 1568.8659; found, 1568.8656.

1. HRMS spectrum of GM1 (d18:1/C20:0)

ESI HRMS(m/z): [M+H]**^+^** calculated for C_75_H_136_N_3_O_31_, 1574.9152; found, 1574.9142.

ESI HRMS(m/z): [M+Na]**^+^** calculated for C_75_H_135_N_3_NaO_31_, 1596.8972; found, 1596.8953.

1. HRMS spectrum of GM1 (d18:1/C16:1)

ESI HRMS(m/z): [M+H]**^+^** calculated for C_71_H_126_N_3_O_31_, 1516.8370; found, 1516.8752.

ESI HRMS(m/z): [M+Na]**^+^** calculated for C_71_H_125_N_3_NaO_31_, 1538.8189; found, 1538.8557.

1. HRMS spectrum of GM1 (d18:1/C18:1)

ESI HRMS(m/z): [M+H]**^+^** calculated for C_73_H_130_N_3_O_31_, 1544.8683; found, 1544.8873.

ESI HRMS(m/z): [M+Na]**^+^** calculated for C_73_H_129_N_3_NaO_31_, 1566.8502; found, 1566.8736.

1. HRMS spectrum of GM1 (d20:1/C16:0)

ESI HRMS(m/z): [M+H]**^+^** calculated for C_73_H_132_N_3_O_31_, 1546.8839; found, 1546.8831.

ESI HRMS(m/z): [M+Na]**^+^** calculated for C_73_H_131_N_3_NaO_31_, 1568.8659; found, 1568.8664.

1. HRMS spectrum of GM1 (d20:1/C18:0)

ESI HRMS(m/z): [M+H]**^+^** calculated for C_75_H_136_N_3_O_31_, 1574.9152; found,1574.9144.

ESI HRMS(m/z): [M+Na]**^+^** calculated for C_75_H_135_N_3_NaO_31_, 1596.8972; found, 1596.9004.

1. HRMS spectrum of GM1 (d20:1/C20:0)

ESI HRMS(m/z): [M+H]**^+^** calculated for C_77_H_140_N_3_O_31_, 1602.9465; found, 1602.9458.

ESI HRMS(m/z): [M+Na]**^+^** calculated for C_77_H_139_N_3_NaO_31_, 1624.9285; found, 1624.9291.

1. HRMS spectrum of GM1 (d20:1/C16:1)

ESI HRMS(m/z): [M+H]**^+^** calculated for C_73_H_130_N_3_O_31_, 1544.8683; found,1544.8698.

ESI HRMS(m/z): [M+Na]**^+^** calculated for C_73_H_129_N_3_NaO_31_, 1566.8502; found,1566.8504.

1. HRMS spectrum of GM1 (d20:1/C18:1)

ESI HRMS(m/z): [M+H]**^+^** calculated for C_75_H_134_N_3_O_31_, 1572.8996; found, 1572.8997.

ESI HRMS(m/z): [M+Na]**^+^** calculated for C_75_H_133_N_3_NaO_31_, 1594.8815; found, 1594.8838.

**Ⅳ. Component comparison and identification of GM1 commercialized drugs named Sygen™ based on MOCECA synthesized GM1 derivatives**

1. Hydrolysis and purification procedure for GM1 enzymatic deacylation system

SA_SCD hydrolysis catalytic system was used to remove the fatty acids existed in GM1 for producing GM1βSph efficiently. The overall reaction system was GM1 (100mg), 0.4% taurodeoxycholate hydrate (TDC) (w/v), and 100 mM CaCl_2_ in 20 ml of 35 mM sodium acetate buffer (pH 5.8) containing 3mg SA_SCD. The hydrolysis reaction was carried out at 37℃ for 12 h, and the progress was monitored by TLC. After the reaction is terminated, the solution mixture was then loaded on the Sep-Pak Vac C18 cartridges (10g) for separation. GM1βSph was eluted and purified separately by 75% gradient methanol and determined by Evaporative Light-scattering Detector (ELSD)-HPLC.

1. HRMS and HPLC spectrum for two components of purified Sygen™
2. HRMS spectrum of purified Sygen™

HRMS spectrum of GM1 (d18:1/C18:0)

ESI HRMS (m/z): [M+H]**^+^** calculated for C_73_H_132_N_3_O_31_, 1546.8839; found, 1546.8822.

ESI HRMS (m/z): [M+Na]**^+^** calculated for C_73_H_131_N_3_NaO_31_, 1568.8659; found, 1568.8655.

HRMS spectrum of GM1 (d20:1/C18:0)

ESI HRMS (m/z): [M+H]**^+^** calculated for C_75_H_136_N_3_O_31_, 1574.9152; found, 1574.9136.

ESI HRMS (m/z): [M+Na]**^+^** calculated for C_75_H_135_N_3_NaO_31_, 1596.8972; found, 1596.8972.


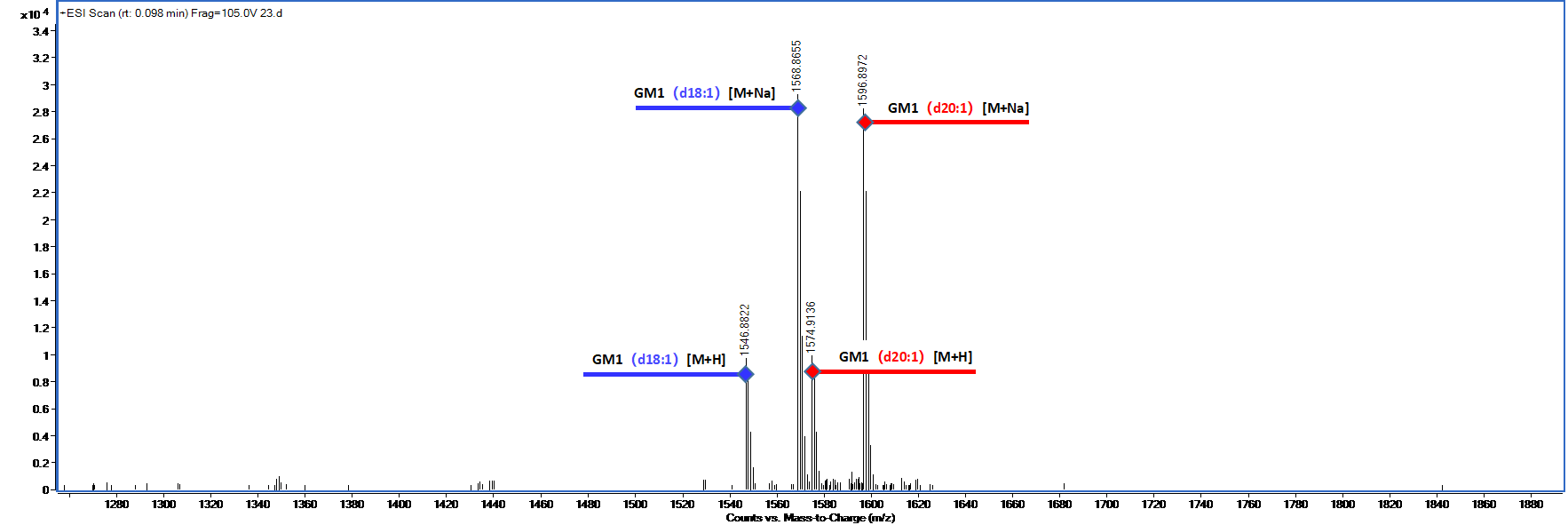


1. HPLC spectrum of purified Sygen™

GM1 (d18:1/C18:0) was characterized by UV-HPLC, T_R_ for GM1 (d18:1/C18:0) = 20.475 min.

GM1 (d20:1/C18:0) was characterized by UV-HPLC, T_R_ for GM1 (d20:1/C18:0) = 32.032 min.


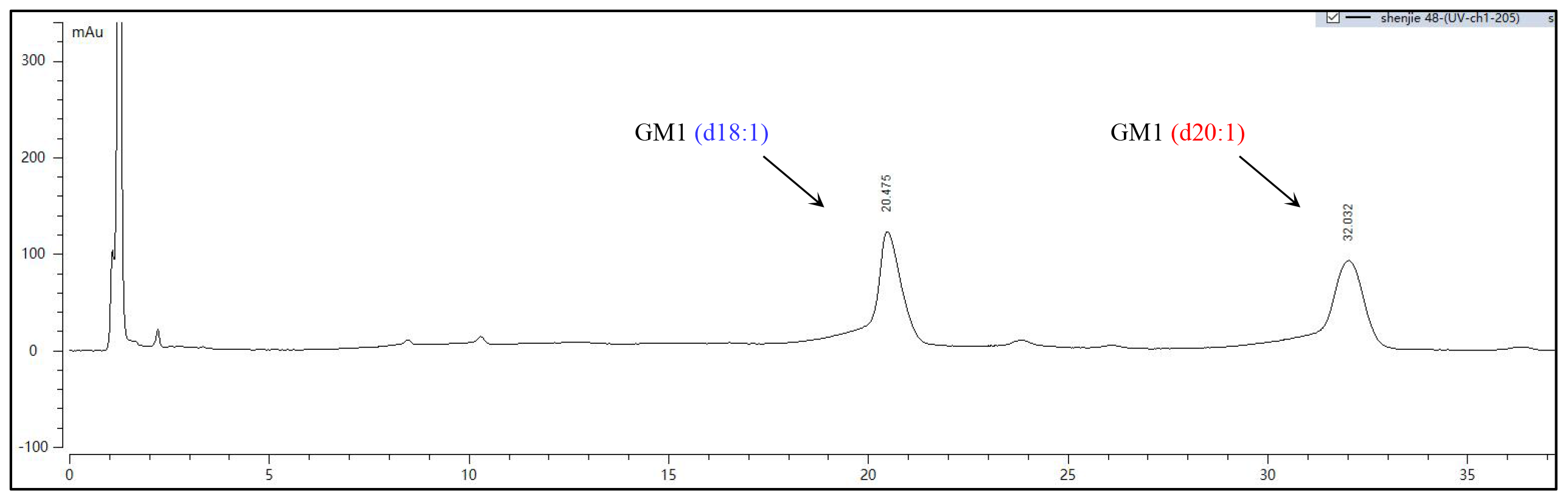


1. HRMS and HPLC spectrum for glycosphingosine products of Sygen™ hydrolyzed by SA_SCD enzymatic deacylation system
2. HRMS spectrum for two components of GM1βSph after SCD hydrolysis and purification

HRMS spectrum of GM1βSph (d18:1)

ESI HRMS (m/z): [M+H]**^+^** calculated for C_55_H_98_N_3_O_30_, 1280.6230; found, 1280.6264.

HRMS spectrum of GM1βSph (d20:1)

ESI HRMS (m/z): [M+H]**^+^** calculated for C_57_H_102_N_3_O_30_, 1308.6543; found, 1308.6576.


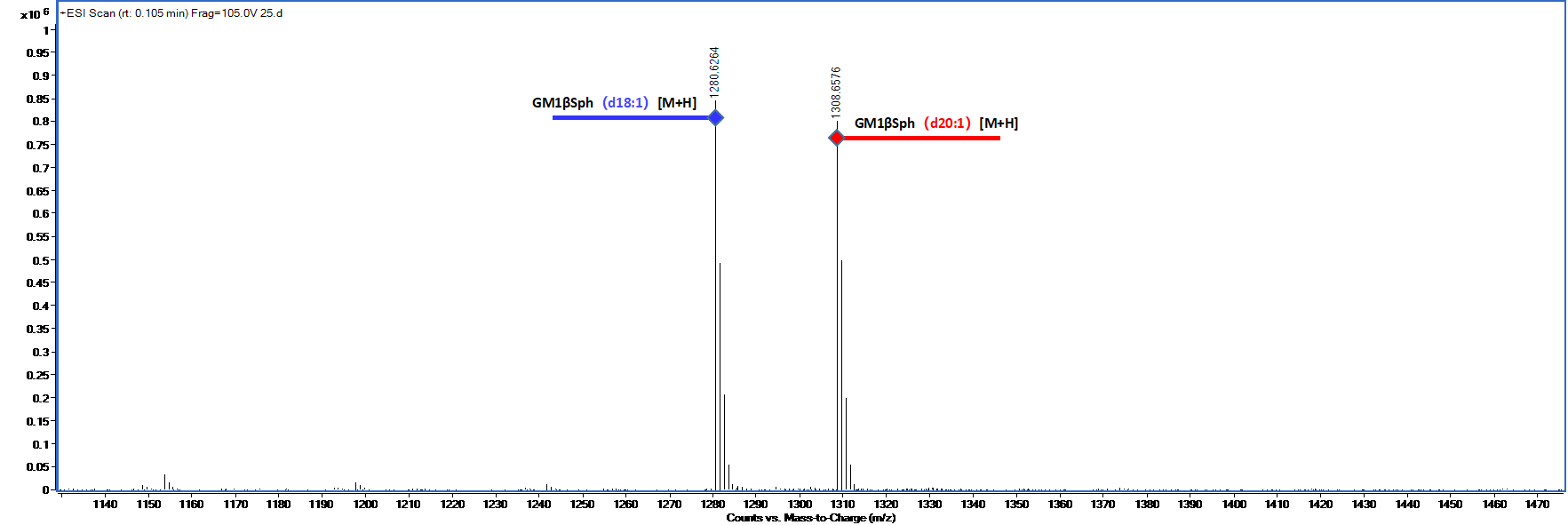


1. HPLC spectrum for two components of GM1βSph after SCD hydrolysis and purification

GM1βSph (d18:1) was characterized by (ELSD)-HPLC, T_R_ for GM1βSph (d18:1) = 7.019 min.

GM1βSph (d20:1) was characterized by (ELSD)-HPLC, T_R_ for GM1βSph (d20:1) = 15.518 min.


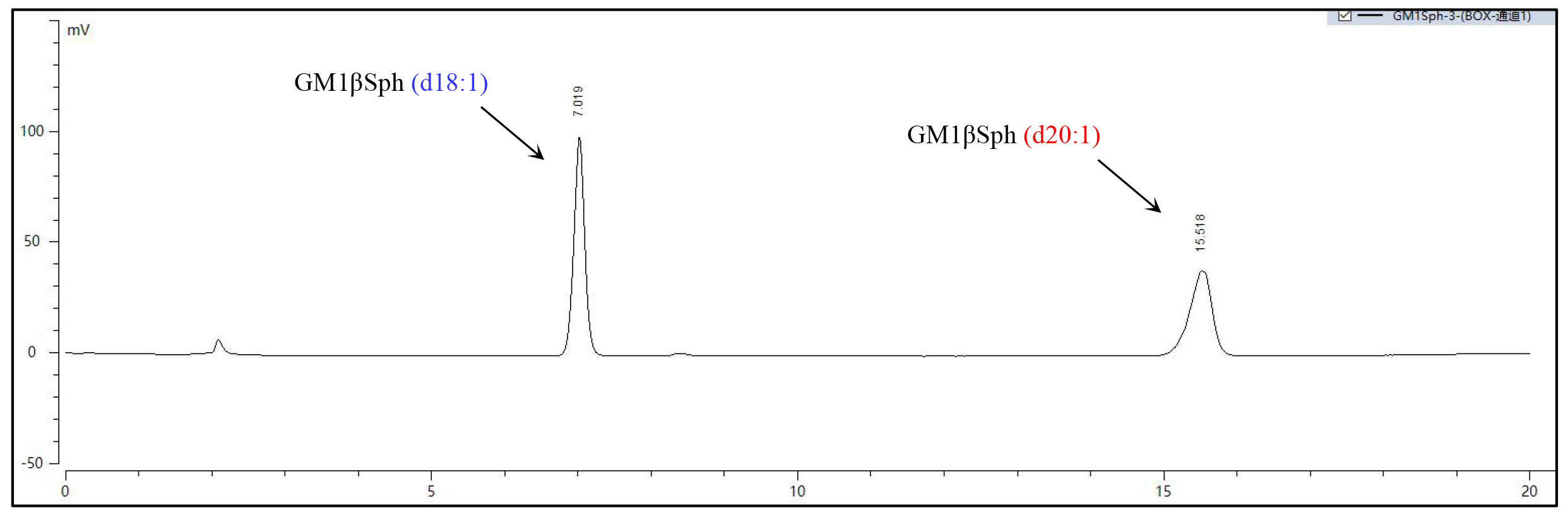


1. HRMS and HPLC spectrum for MOCECA synthesized GM1βSph (d18:1) and GM1βSph (d20:1)

HRMS and HPLC spectrum of GM1βSph derivatives

1. HRMS spectrum of GM1βSph (d18:1)

ESI HRMS (m/z): [M+H]**^+^** calculated for C_55_H_98_N_3_O_30_, 1280.6230; found, 1280.6264.

GM1βSph (d18:1) was characterized by ELSD-HPLC, T_R_ = 6.773 min.


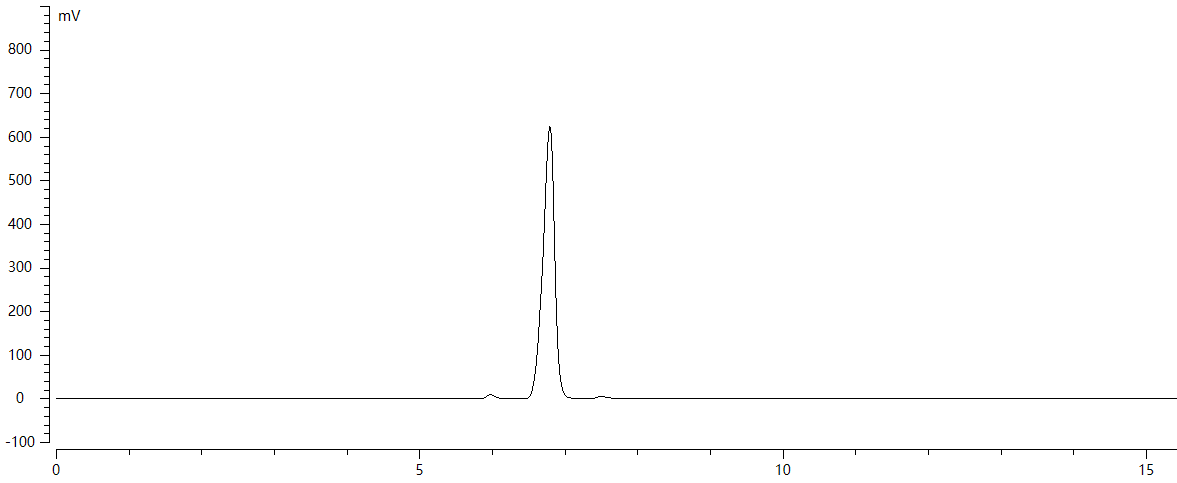


1. HRMS spectrum of GM1βSph (d20:1)

ESI HRMS(m/z): [M+H]**^+^** calculated for C_57_H_102_N_3_O_30_, 1308.6543; found,1308.6576.

GM1βSph (d20:1) was characterized by ELSD-HPLC, T_R_ = 15.148 min.


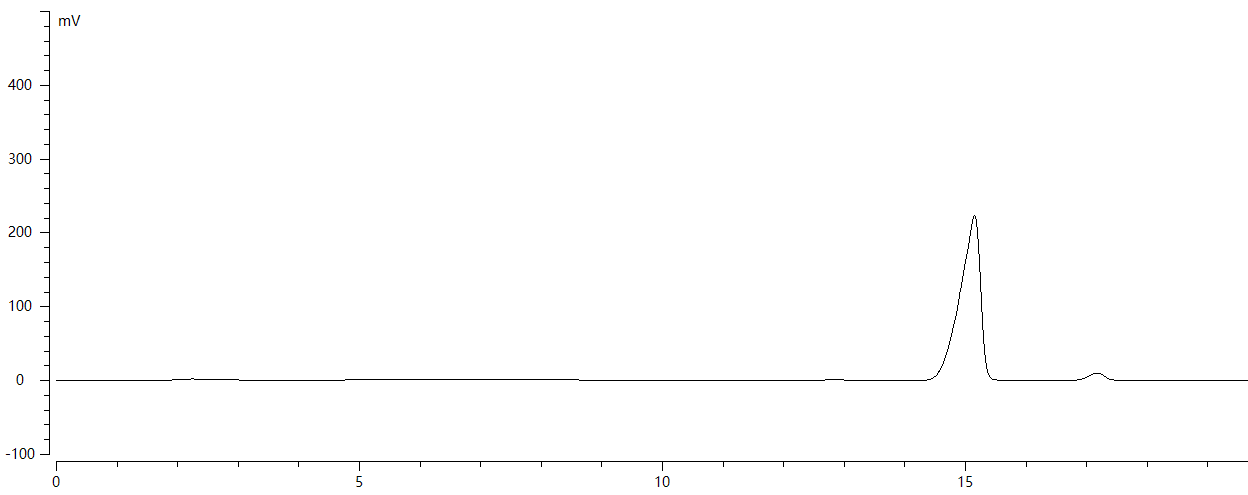


1. HRMS and HPLC spectrum for glycosylsphingosine products of MOCECA synthesized GM1 derivatives mixed with GM1(d18:1/C18:0) and GM1(d20:1/C18:0) hydrolyzed by SCD enzymatic deacylation system

HRMS spectrum for two components of GM1βSph after SCD hydrolysis and purification

HRMS spectrum of GM1βSph (d18:1)

ESI HRMS (m/z): [M+H]**^+^** calculated for C_55_H_98_N_3_O_30_, 1280.6230; found, 1280.6264.

HRMS spectrum of GM1βSph (d20:1)

ESI HRMS (m/z): [M+H]**^+^** calculated for C_57_H_102_N_3_O_30_, 1308.6543; found, 1308.6579.

HPLC spectrum of GM1βSph derivatives

GM1βSph (d18:1) was characterized by (ELSD)-HPLC, T_R_ for GM1βSph (d18:1) = 6.852 min.

GM1βSph (d20:1) was characterized by (ELSD)-HPLC, T_R_ for GM1βSph (d20:1) = 14.599 min.


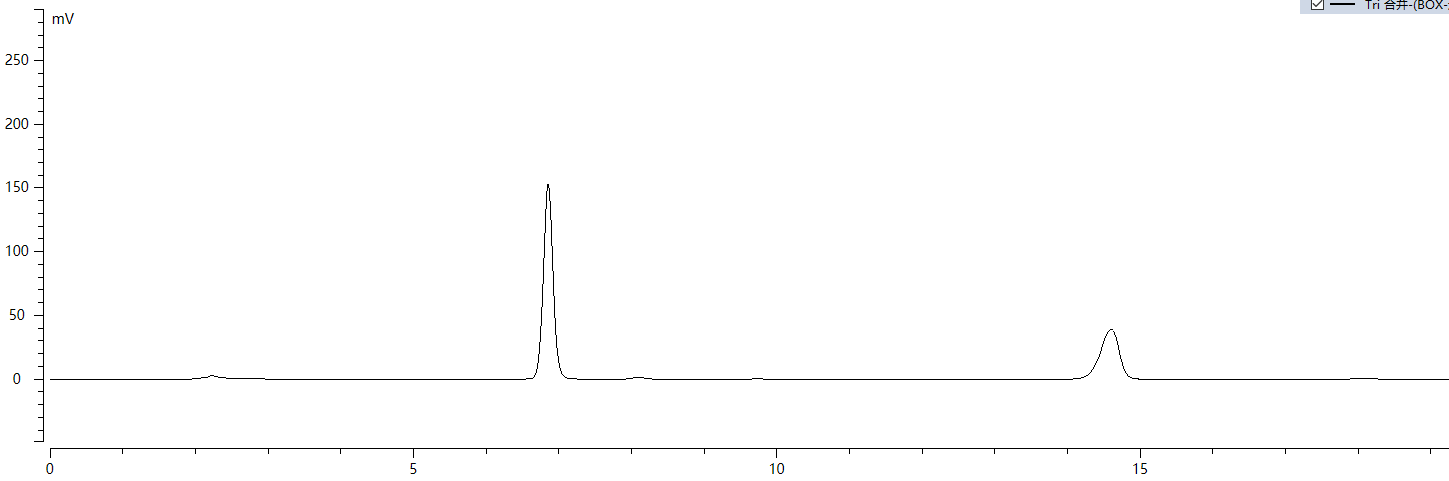


1. HRMS and HPLC spectrum for glycosylsphingosine products of MOCECA synthesized GM1 derivatives mixed with GM1(d18:1/C18:0) and GM1(d18:1/C20:0) hydrolyzed by SCD enzymatic deacylation system

HRMS spectrum for glycosylsphingosine products of MOCECA synthesized GM1 derivatives mixed with GM1(d18:1/C18:0) and GM1(d18:1/C20:0) after SCD hydrolysis and purification

ESI HRMS(m/z) of GM1βSph (d18:1)

ESI HRMS(m/z): [M+H]**^+^** calculated for C_55_H_98_N_3_O_30_, 1280.6230; found, 1280.6267.

ESI HRMS(m/z): [M+Na]**^+^** calculated for C_55_H_97_N_3_NaO_30_, 1302.6049; found, 1302.6064.

HPLC spectrum for glycosylsphingosine products of MOCECA synthesized GM1 derivatives mixed with GM1(d18:1/C18:0) and GM1(d18:1/C20:0) after SCD hydrolysis and purification

GM1βSph (d18:1) was characterized by (ELSD)-HPLC, T_R_ for GM1βSph (d18:1) = 6.792 min.

**
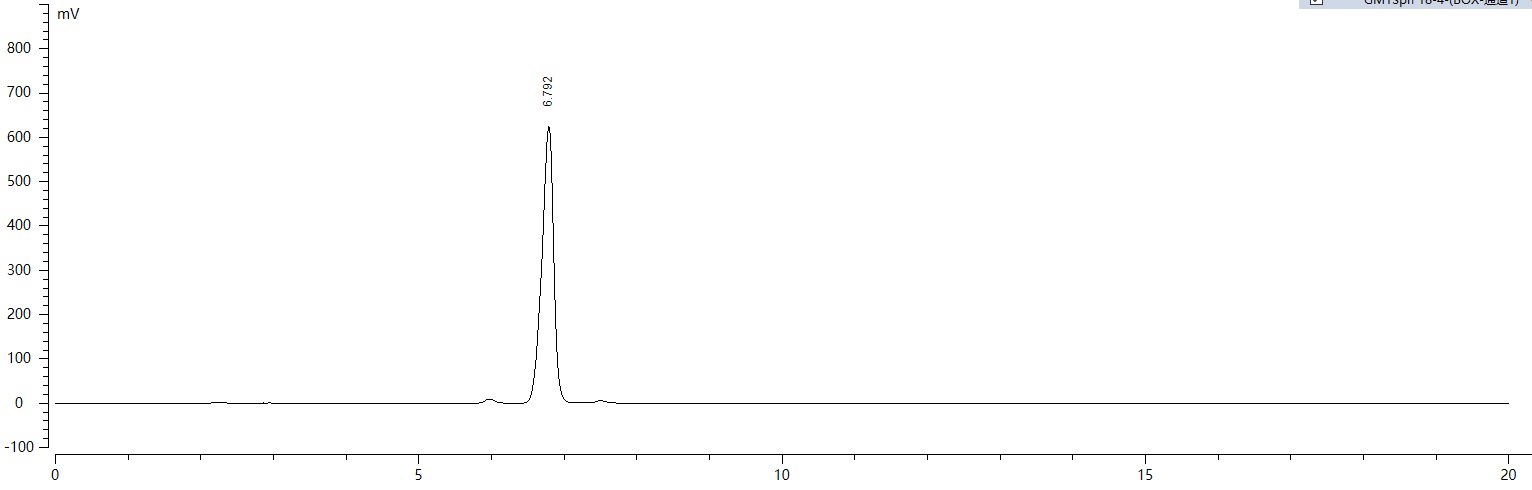
**

**Ⅴ. Supplementary References**

1. Yu, H., Yu, H., Karpel, R. & Chen, X. Chemoenzymatic synthesis of CMP-sialic acid derivatives by a one-pot two-enzyme system: comparison of substrate flexibility of three microbial CMP-sialic acid synthetases. *Bioorg Med Chem* **12**, 6427-35 (2004).

2. Yu, H. et al. A multifunctional Pasteurella multocida sialyltransferase: a powerful tool for the synthesis of sialoside libraries. *J Am Chem Soc* **127**, 17618-9 (2005).

3. Lau, K. et al. Highly efficient chemoenzymatic synthesis of beta1-4-linked galactosides with promiscuous bacterial beta1-4-galactosyltransferases. *Chem Commun (Camb)* **46**, 6066-8 (2010).

4. Li, Y. et al. Substrate promiscuity of N-acetylhexosamine 1-kinases. *Molecules* **16**, 6396-407 (2011).

5. Guan, W., Cai, L. & Wang, P.G. Highly efficient synthesis of UDP-GalNAc/GlcNAc analogues with promiscuous recombinant human UDP-GalNAc pyrophosphorylase AGX1. *Chemistry* **16**, 13343-5 (2010).

6. Yu, H. et al. Sequential One-Pot Multienzyme Chemoenzymatic Synthesis of Glycosphingolipid Glycans. *J Org Chem* **81**, 10809-10824 (2016).

7. Chen, X. et al. Reassembled biosynthetic pathway for large-scale carbohydrate synthesis: alpha-Gal epitope producing "superbug". *Chembiochem* **3**, 47-53 (2002).

8. Muthana, M.M. et al. Improved one-pot multienzyme (OPME) systems for synthesizing UDP-uronic acids and glucuronides. *Chem Commun (Camb)* **51**, 4595-8 (2015).

9. Malekan, H. et al. One-pot multi-enzyme (OPME) chemoenzymatic synthesis of sialyl-Tn-MUC1 and sialyl-T-MUC1 glycopeptides containing natural or non-natural sialic acid. *Bioorg Med Chem* **21**, 4778-85 (2013).

10. Cheng, J. et al. Multifunctionality of Campylobacter jejuni sialyltransferase CstII: characterization of GD3/GT3 oligosaccharide synthase, GD3 oligosaccharide sialidase, and trans-sialidase activities. *Glycobiology* **18**, 686-97 (2008).

11. Hancock, S.M., Rich, J.R., Caines, M.E., Strynadka, N.C. & Withers, S.G. Designer enzymes for glycosphingolipid synthesis by directed evolution. *Nat Chem Biol* **5**, 508-14 (2009).

12. Vaughan, M.D. et al. Glycosynthase-mediated synthesis of glycosphingolipids. *J Am Chem Soc* **128**, 6300-1 (2006).

13. Huang, F.T., Han, Y.B., Feng, Y. & Yang, G.Y. A facile method for controlling the reaction equilibrium of sphingolipid ceramide N-deacylase for lyso-glycosphingolipid production. *J Lipid Res* **56**, 1836-42 (2015).

14. Ostendorf, M., Dijkink, J., Rutjes, F. & Hiemstra, H. (S)-pyroglutamic acid, (S)-malic acid, and (S)-serine as useful starting materials in the synthesis of enantiopure hydroxyamidines. *Eur J Org Chem* **2000**, 115-124 (2000).

15. Kundu, I., Maitra, R., Jana, M. & Chattopadhyay, S.K. A Stereodivergent Route to Four Stereoisomeric 3 '-Acetoxycyclopentenylglycine Derivatives. *Synthesis-Stuttgart* **44**, 304-310 (2012).

16. Clyne, D.S. & Weiler, L. The synthesis of 14-membered macrocyclic ethers. *Tetrahedron* **55**, 13659-13682 (1999).

17. Yang, H. & Liebeskind, L.S. A concise and scalable synthesis of high enantiopurity (-)-D-erythro-sphingosine using peptidyl thiol ester-boronic acid cross-coupling. *Org Lett* **9**, 2993-5 (2007).
