# Supplementary material for: A modular chemoenzymatic cascade strategy for the structure-customized assembly of ganglioside analogs
Source: Commun Chem. 2024 Jan 18;7:17. doi: 10.1038/s42004-024-01102-9 (PMC10796935; doi:10.1038/s42004-024-01102-9)
Supplement: Supplementary file 3 — Description of Additional Supplementary Files [file 42004_2024_1102_MOESM3_ESM.pdf]

Description of Additional Supplementary Files

File name: Supplementary Data 1

Description: NMR Spectra for compounds in MOCECA strategy
