# Supplementary material for: A modular chemoenzymatic cascade strategy for the structure-customized assembly of ganglioside analogs
Source: Commun Chem. 2024 Jan 18;7:17. doi: 10.1038/s42004-024-01102-9 (PMC10796935; doi:10.1038/s42004-024-01102-9)

**Supplementary Data of NMR Spectra for compounds in MOCECA strategy**

**A Modular Chemoenzymatic Cascade Strategy for the Structure-Customized Assembly of Ganglioside Analogs**

**Xuefeng Jin^1,2†^, Hanchao Cheng^3,4†^, Xiaohui Chen^1^, Xuefeng Cao^5^, Cong Xiao^5^, Fengling Ding^5^, Huirong Qu^5^, Peng George Wang^4^, Yan Feng^1^, Guang-Yu Yang^1^***

^1^ State Key Laboratory of Microbial Metabolism, Joint International Research Laboratory of Metabolic & Developmental Sciences, School of Life Sciences and Biotechnology, Shanghai Jiao Tong University, Shanghai 200240, China.

^2^ Department of Clinical Pharmaceutics, The People’s Hospital of Guangxi Zhuang Autonomous Region, Nanning, 530021, China.

^3^ School of Food and Drug, Shenzhen Polytechnic University, Shenzhen 518055, China.

^4^ Department of Pharmacology, Key University Laboratory of Metabolism and Health of Guangdong, School of Medicine, Southern University of Science and Technology, Guangdong 518055, China.

^5^ Glycogene LLC, 10th Floor, Building 3, Wuhan Precision Medicine Industrial Base, Gaokeyuan Road, East Lake New Technology Development Zone, Wuhan 430074, China.

†These authors contributed equally to this work.

*Corresponding author’s email: yanggy@sjtu.edu.cn

**NMR Spectra for compounds by MOCECA strategy**

^1^H and ^13^C NMR spectra of compound **29**


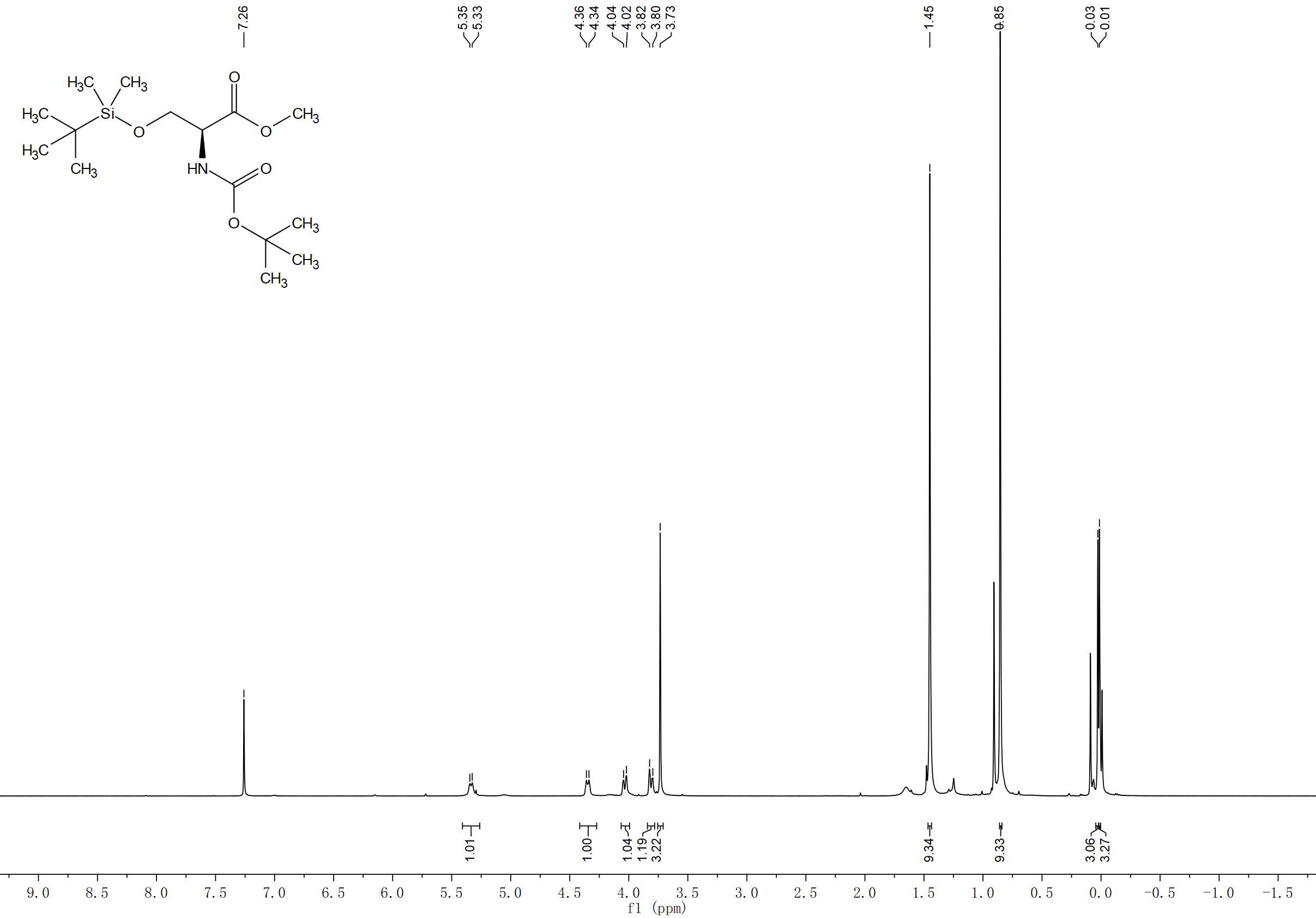


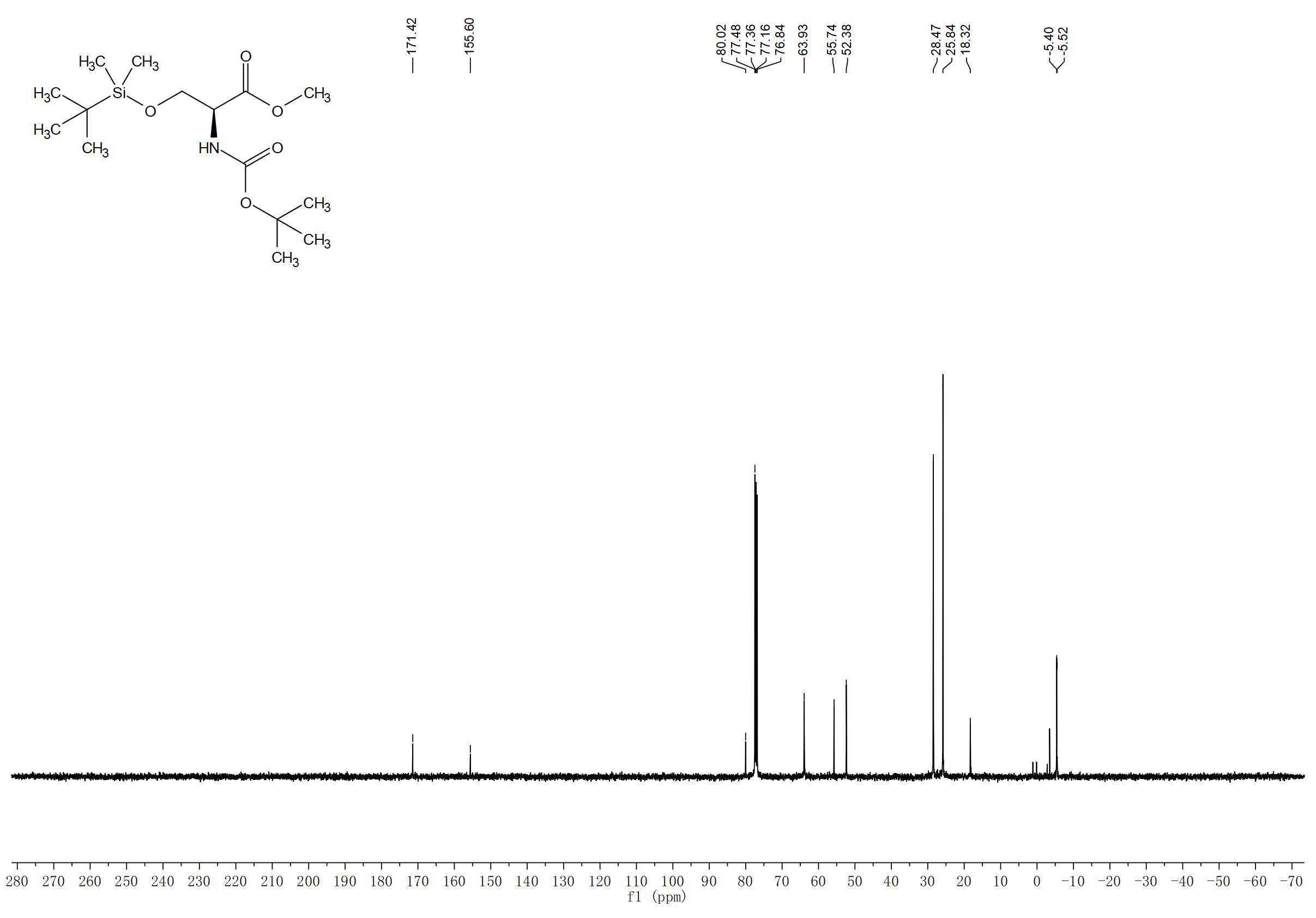


^1^H and ^13^C NMR spectra of compound **30**


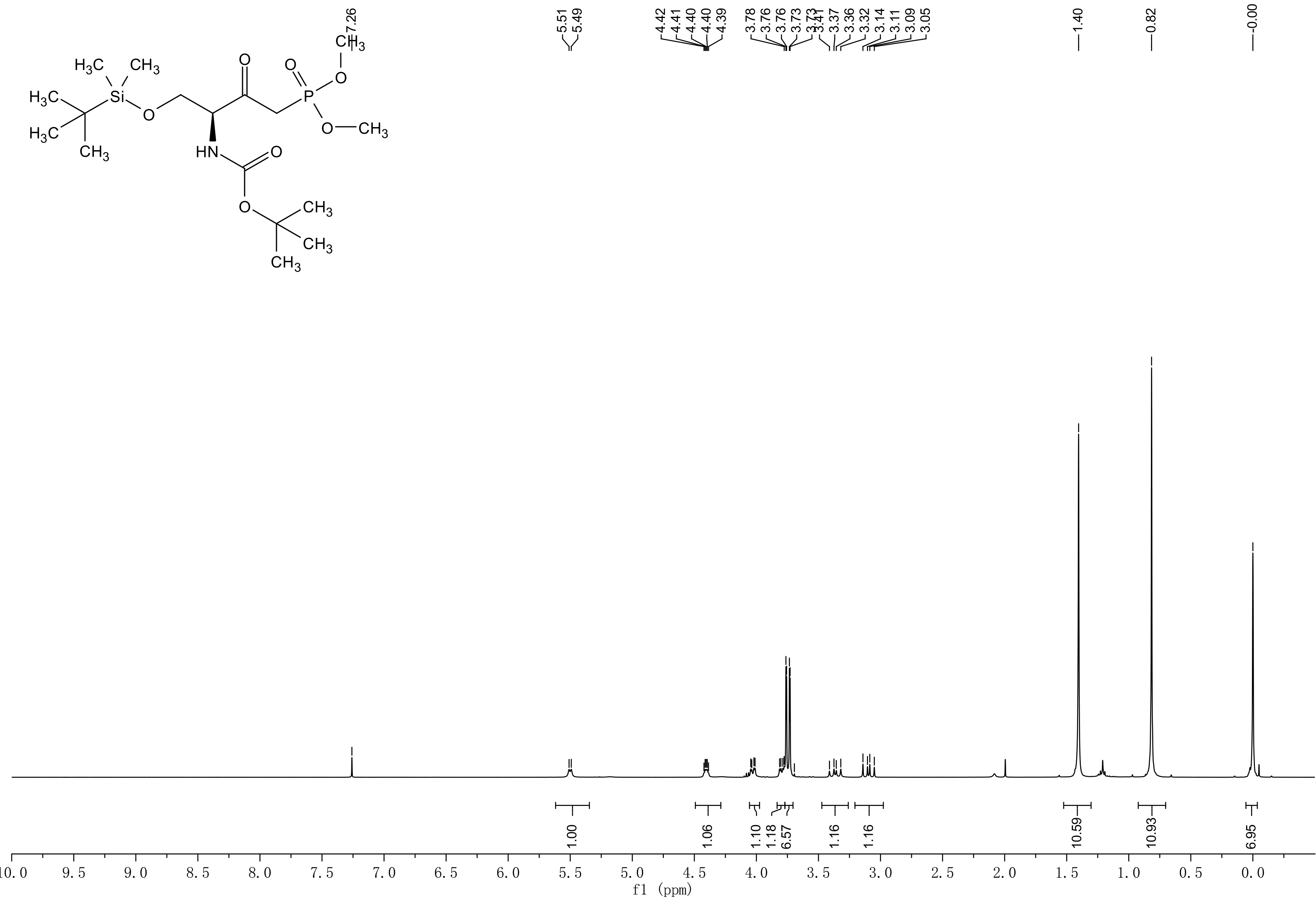


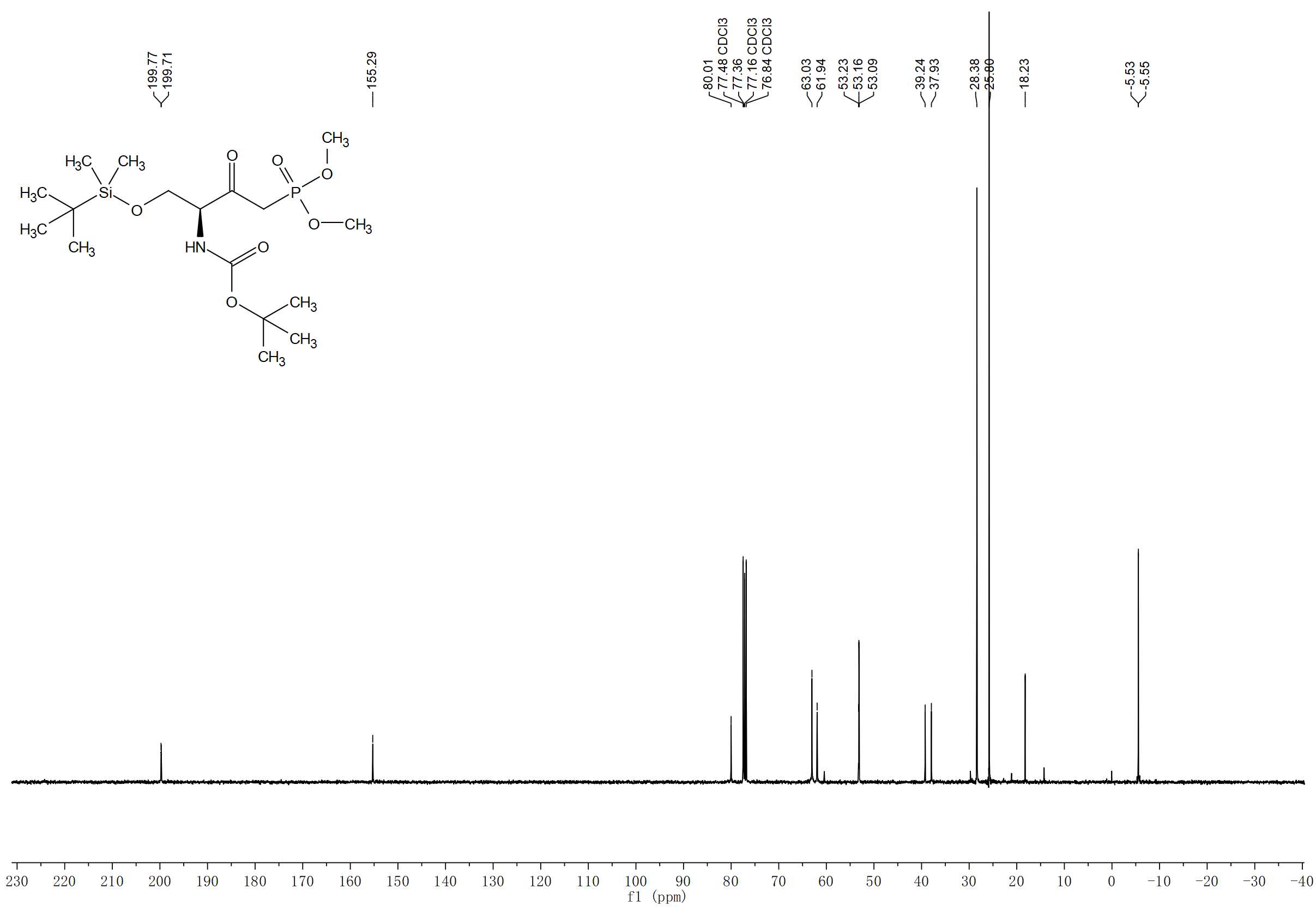


^1^H and ^13^C NMR spectra of compound **31**


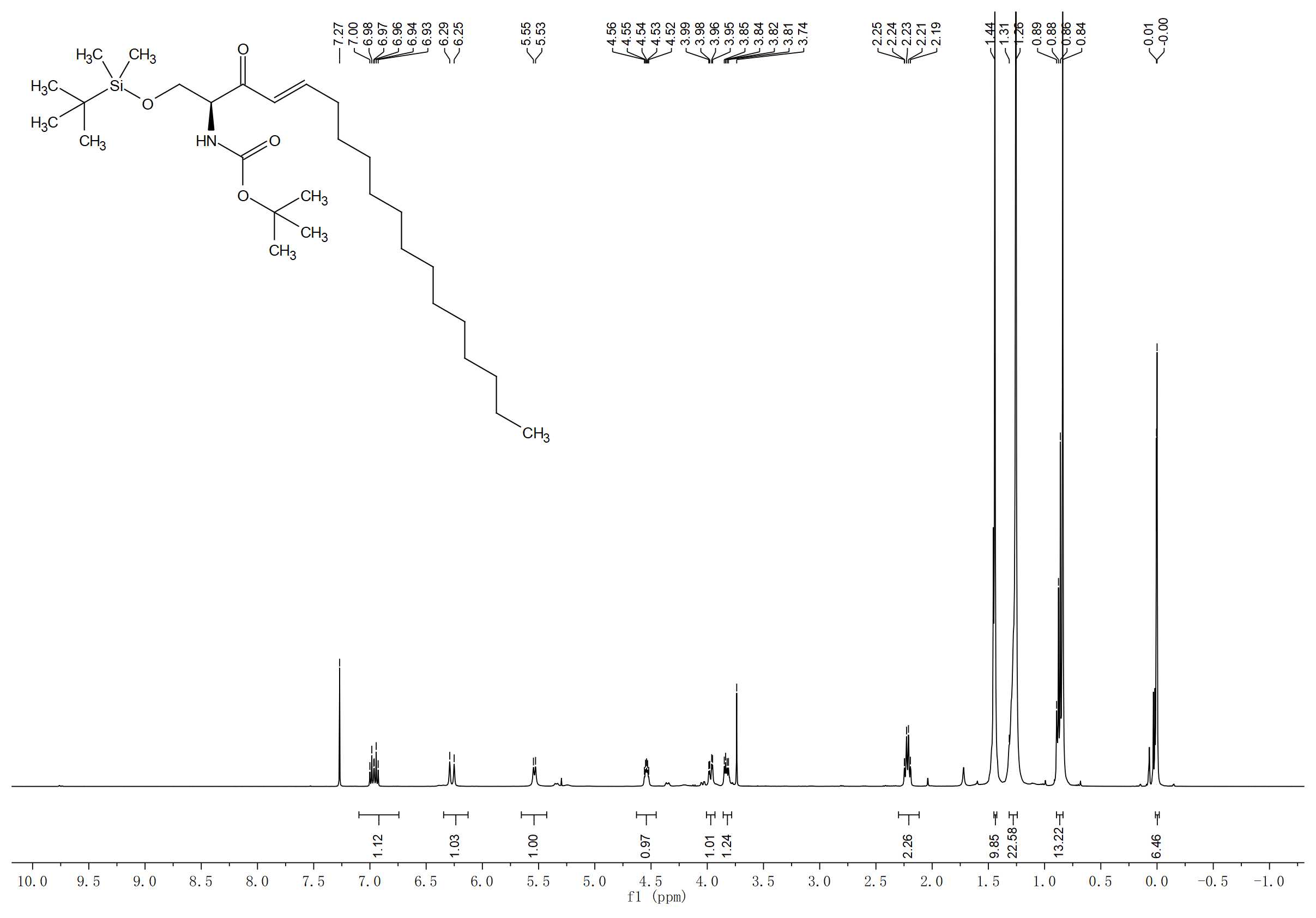


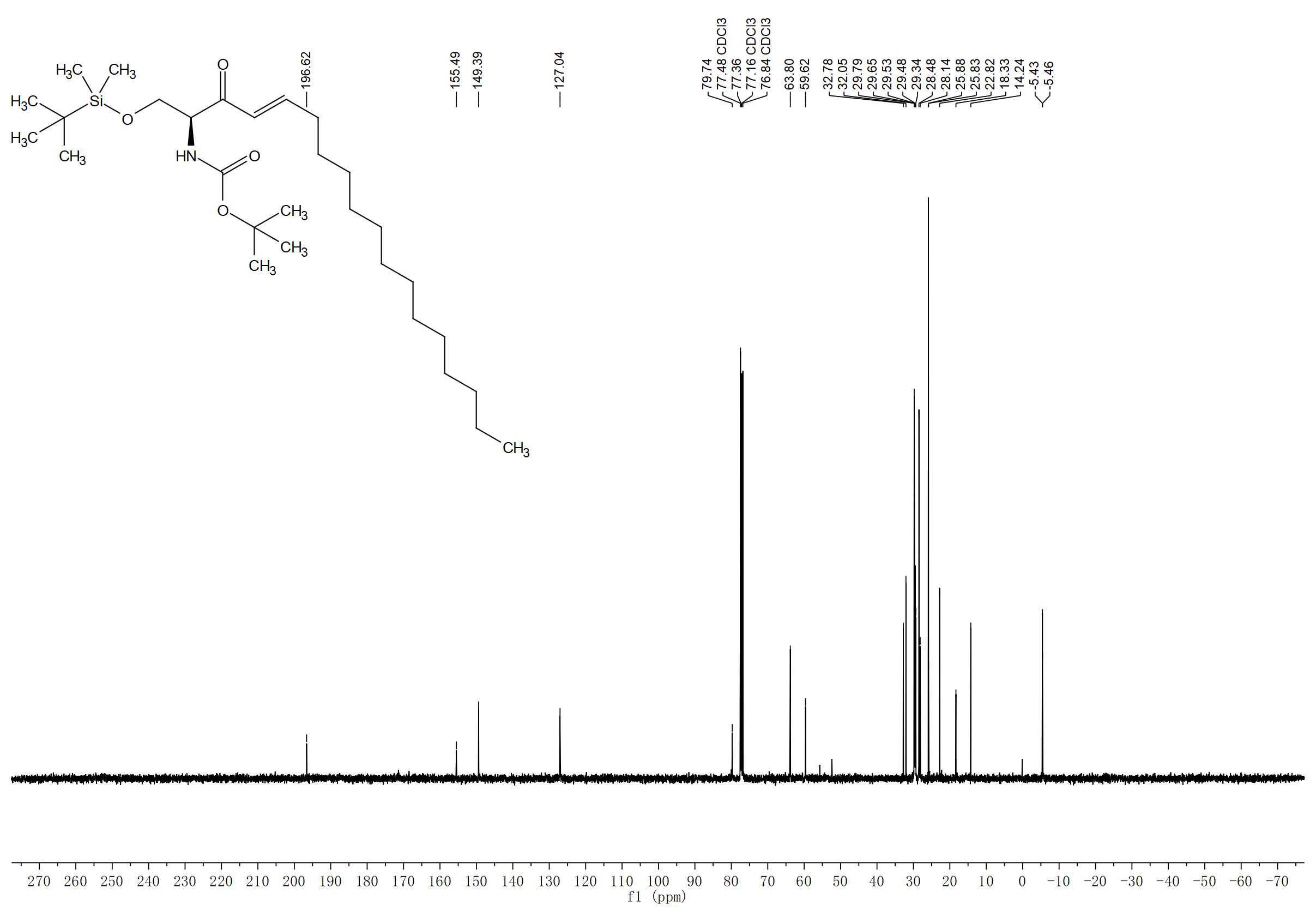


^1^H and ^13^C NMR spectra of compound **32**


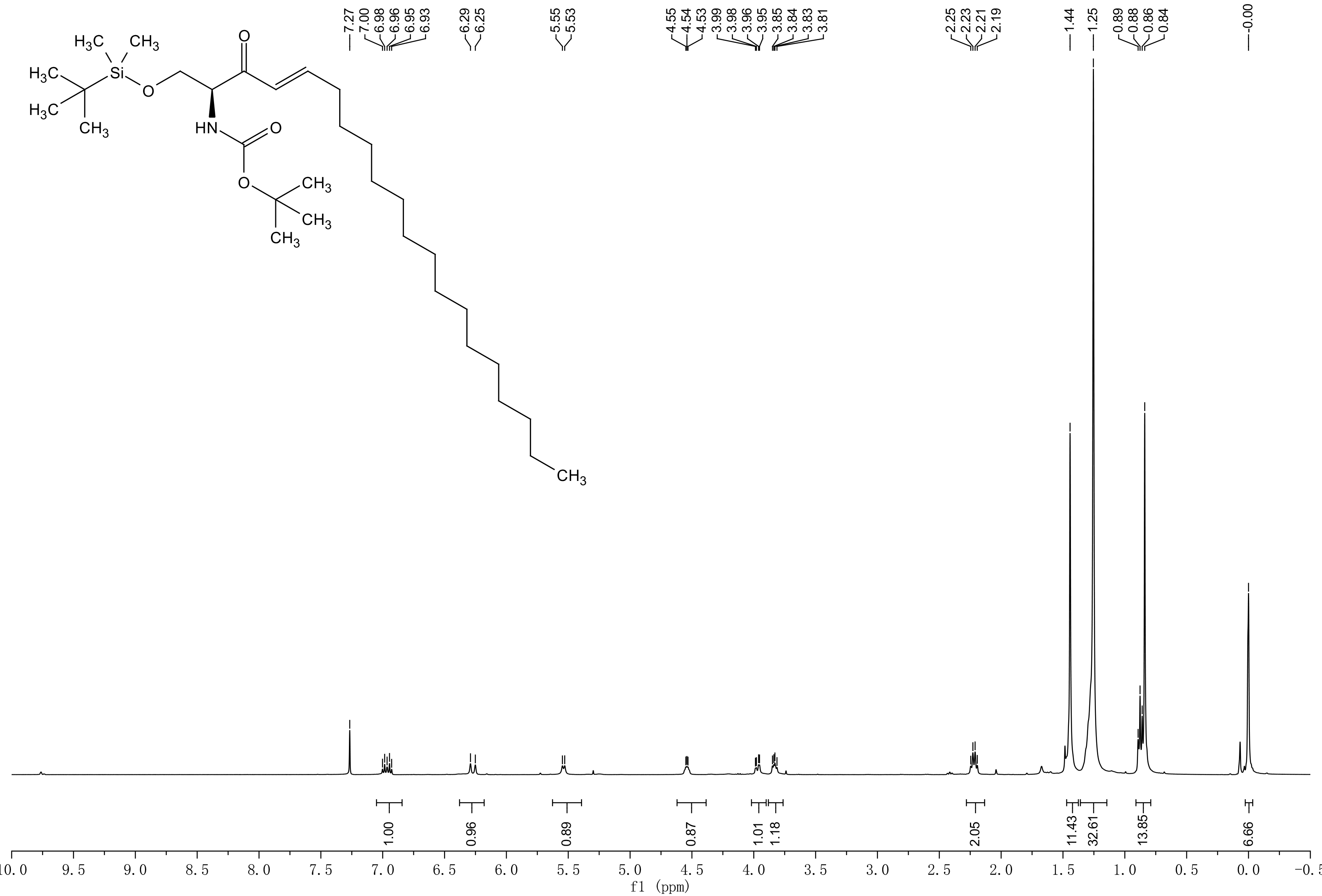


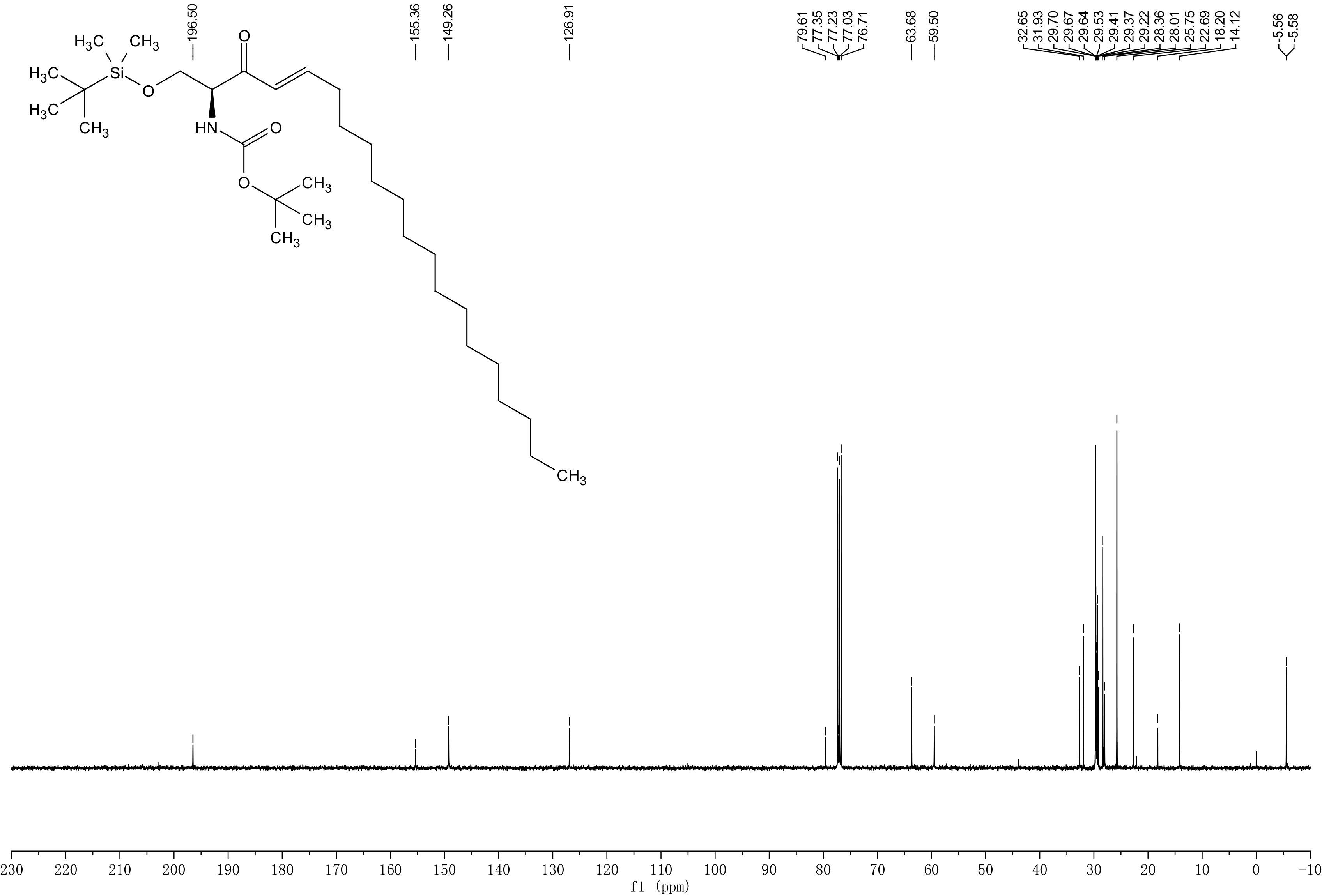


^1^H and ^13^C NMR spectra of compound **35**


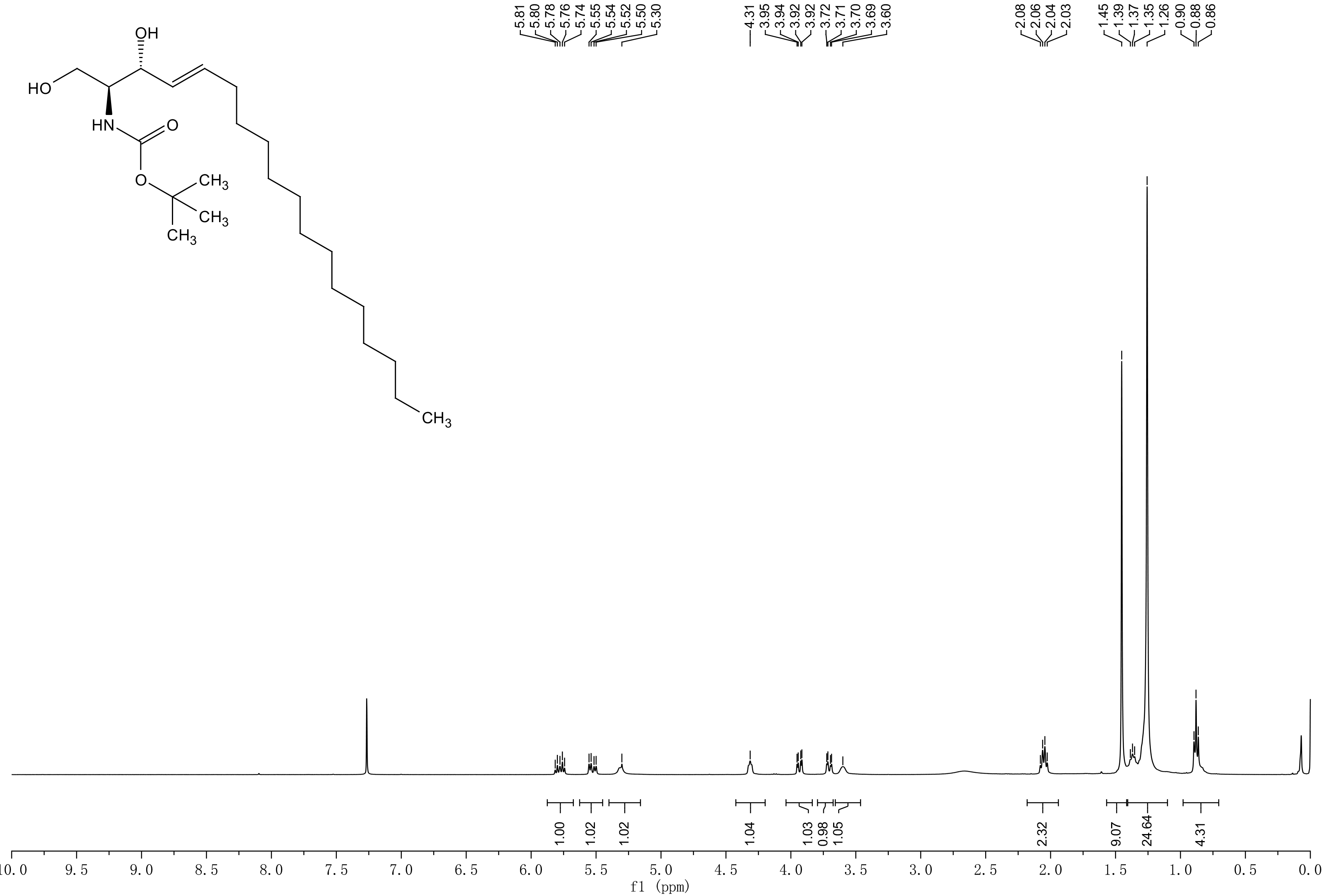


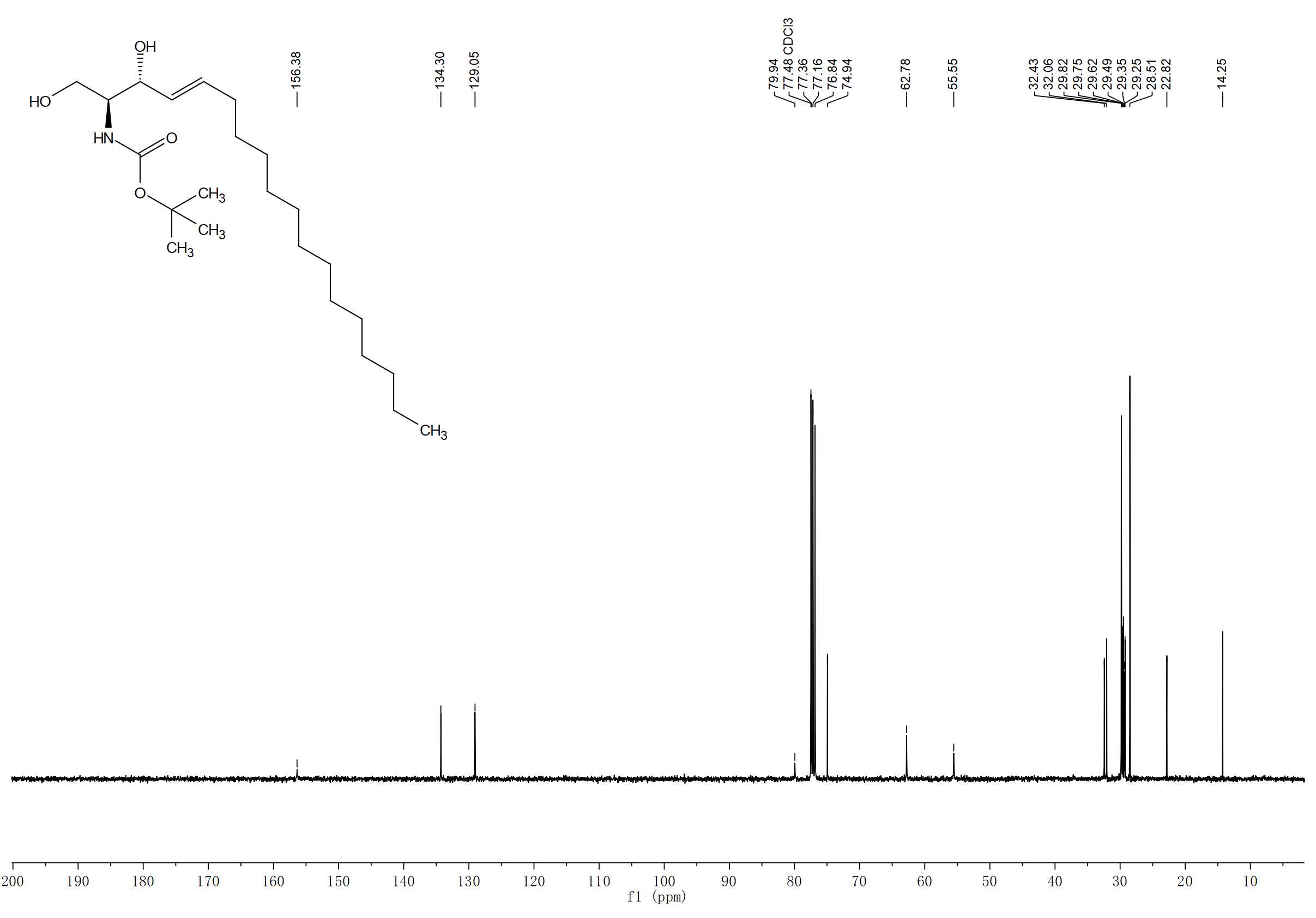


^1^H and ^13^C NMR spectra of compound **36**


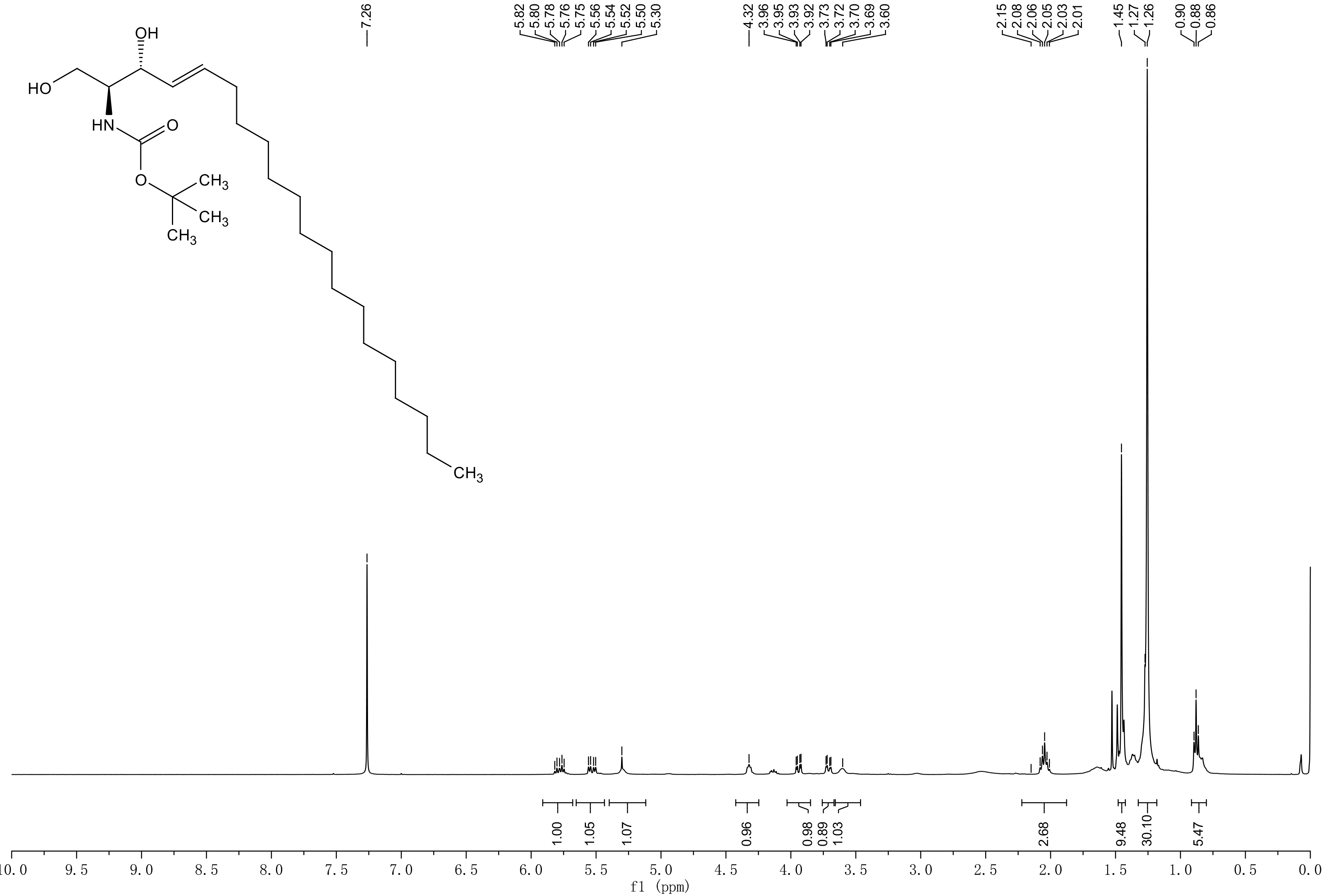


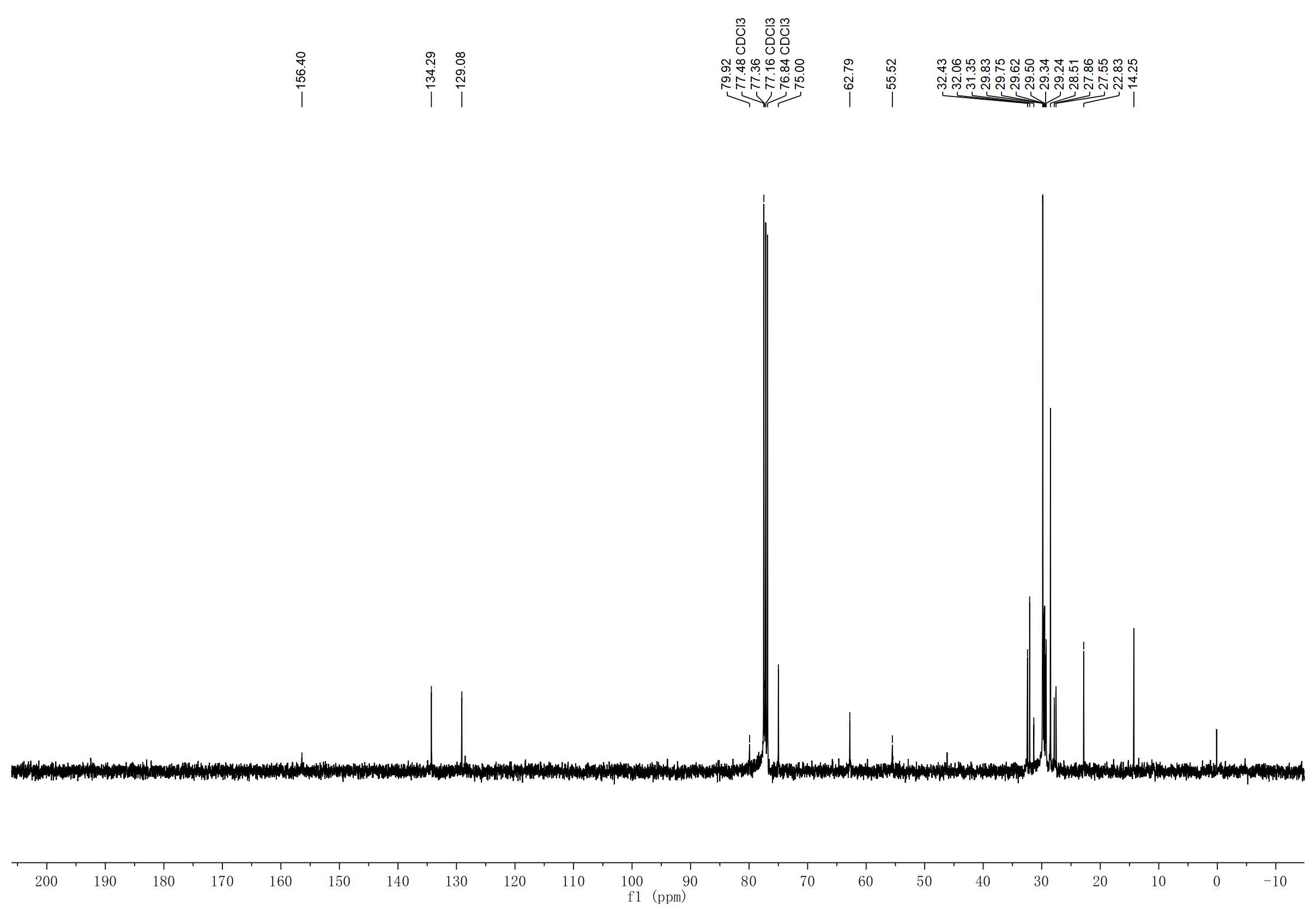


^1^H and ^13^C NMR spectra of D-sphingosine (d18:1) **(16)**

^
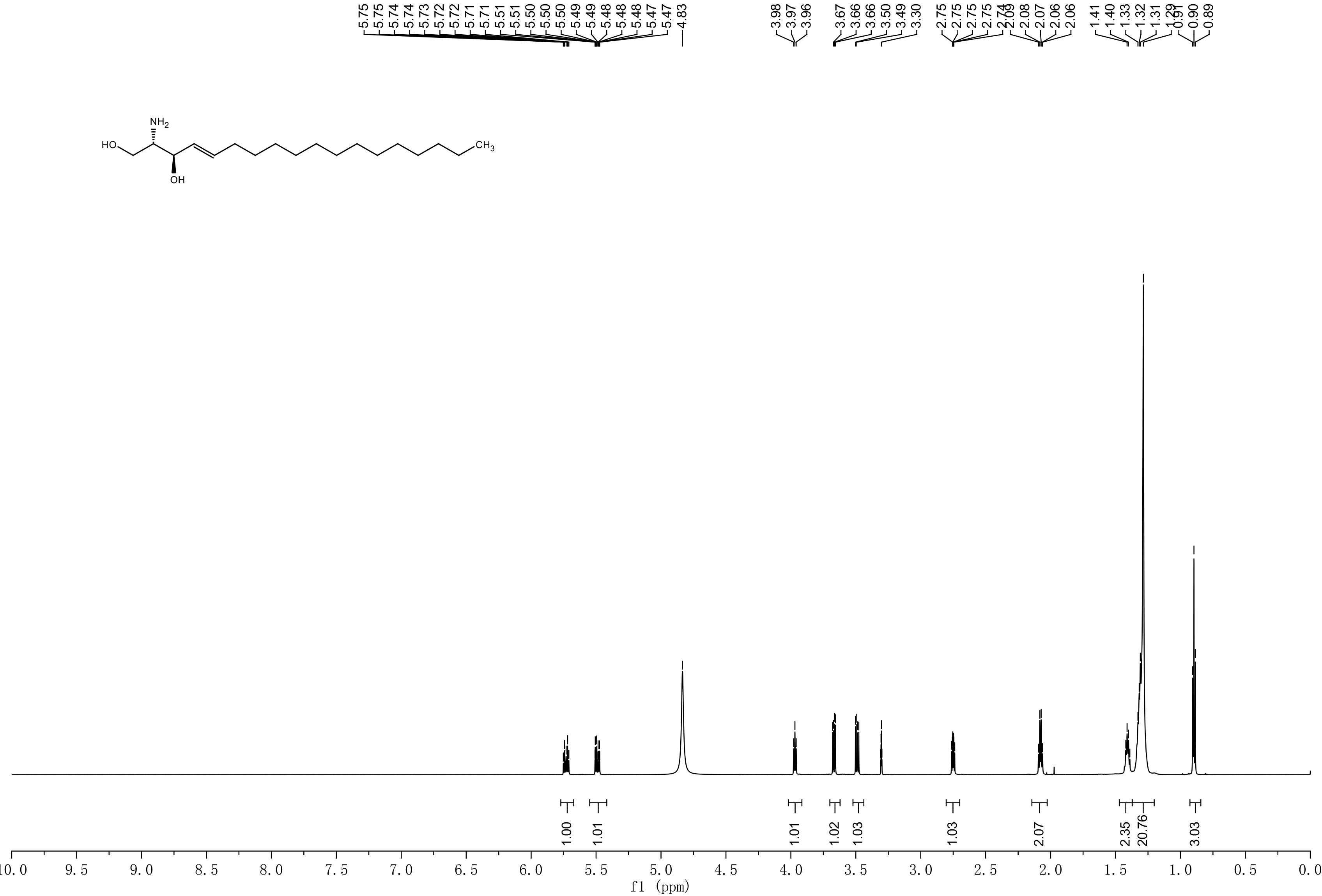
^

^
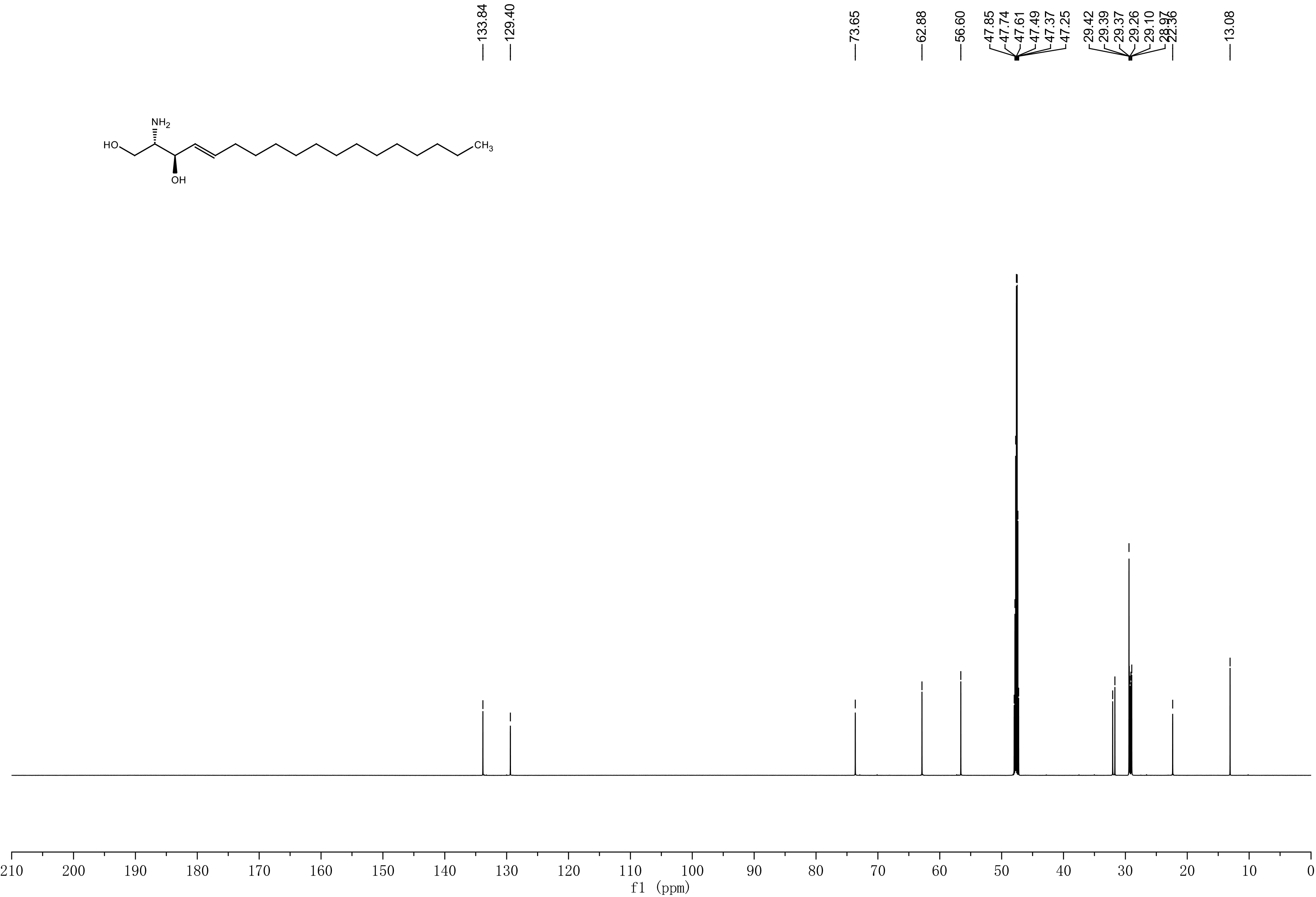
^

^1^H and ^13^C NMR spectra of D-sphingosine (d20:1) **(17)**

^
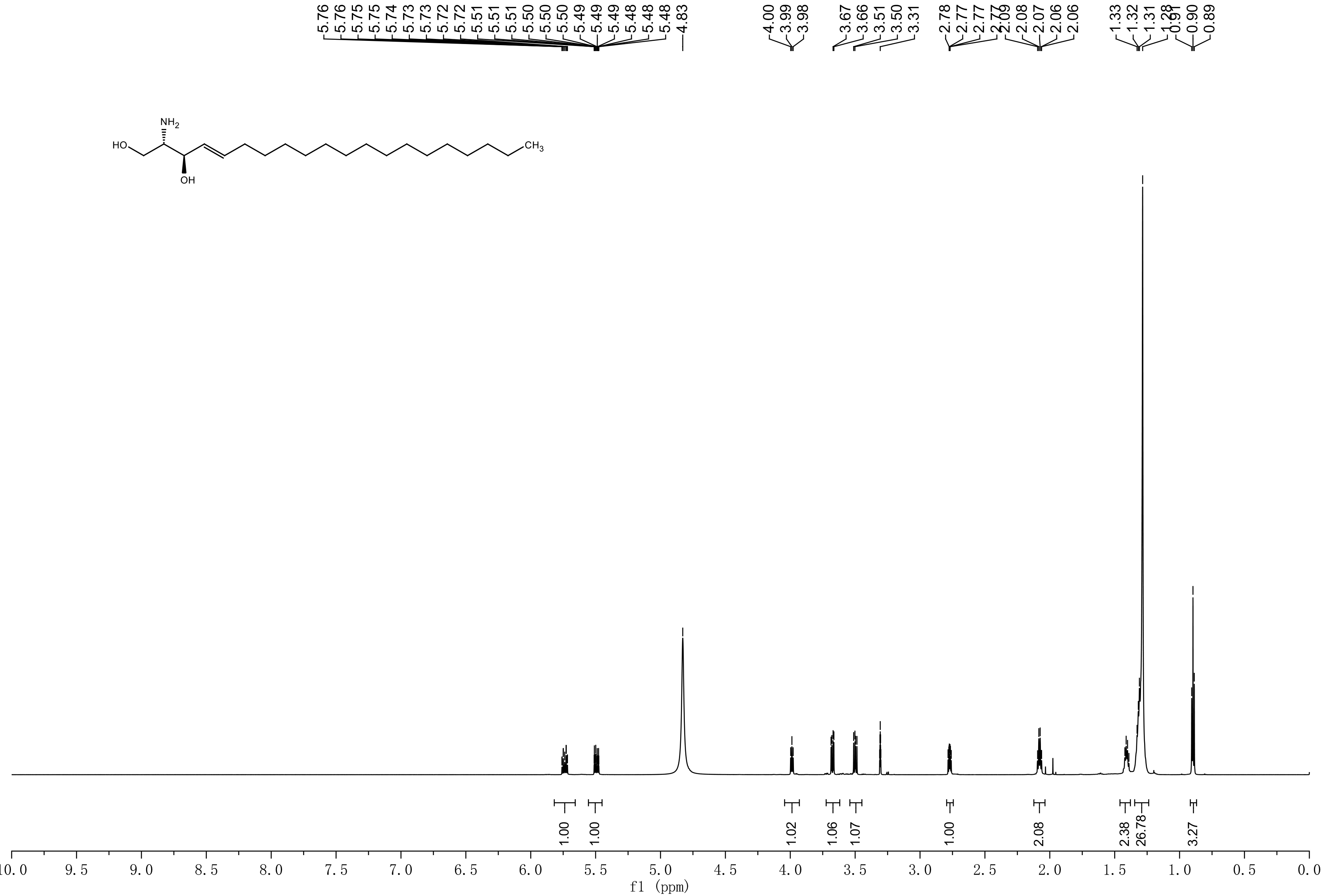
^

^
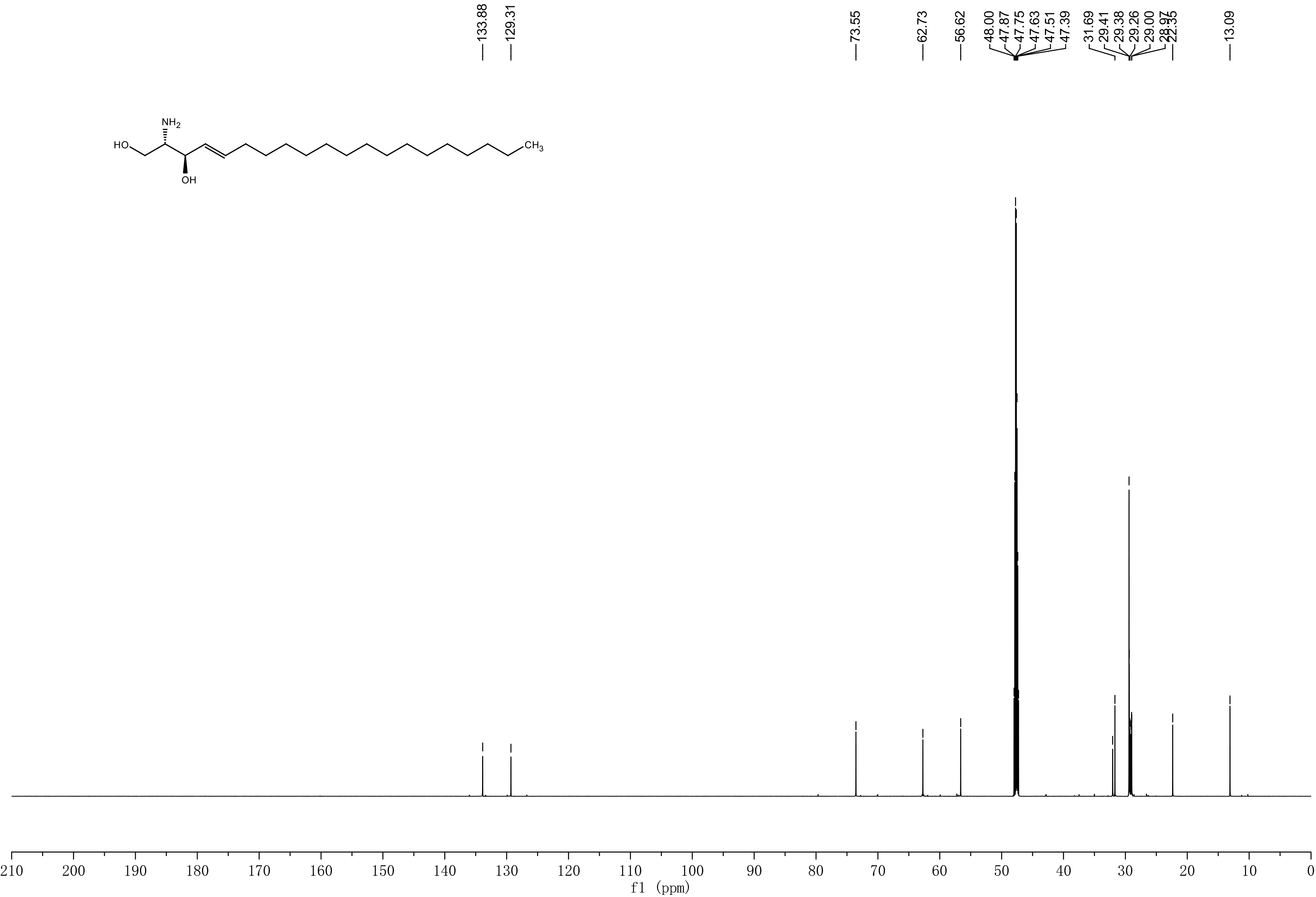
^

^1^H and ^13^C NMR spectra of GM3βSph **(6)**


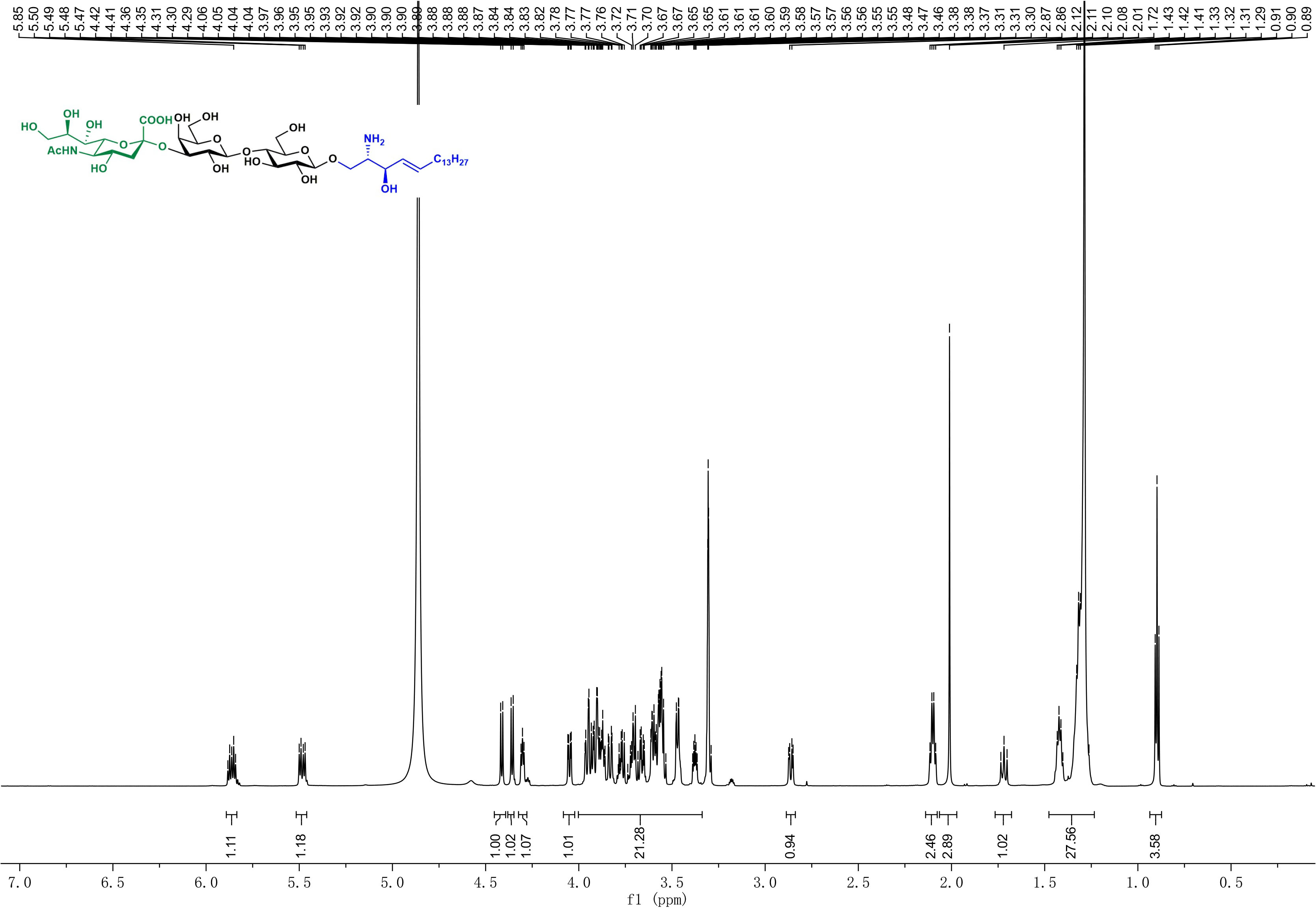


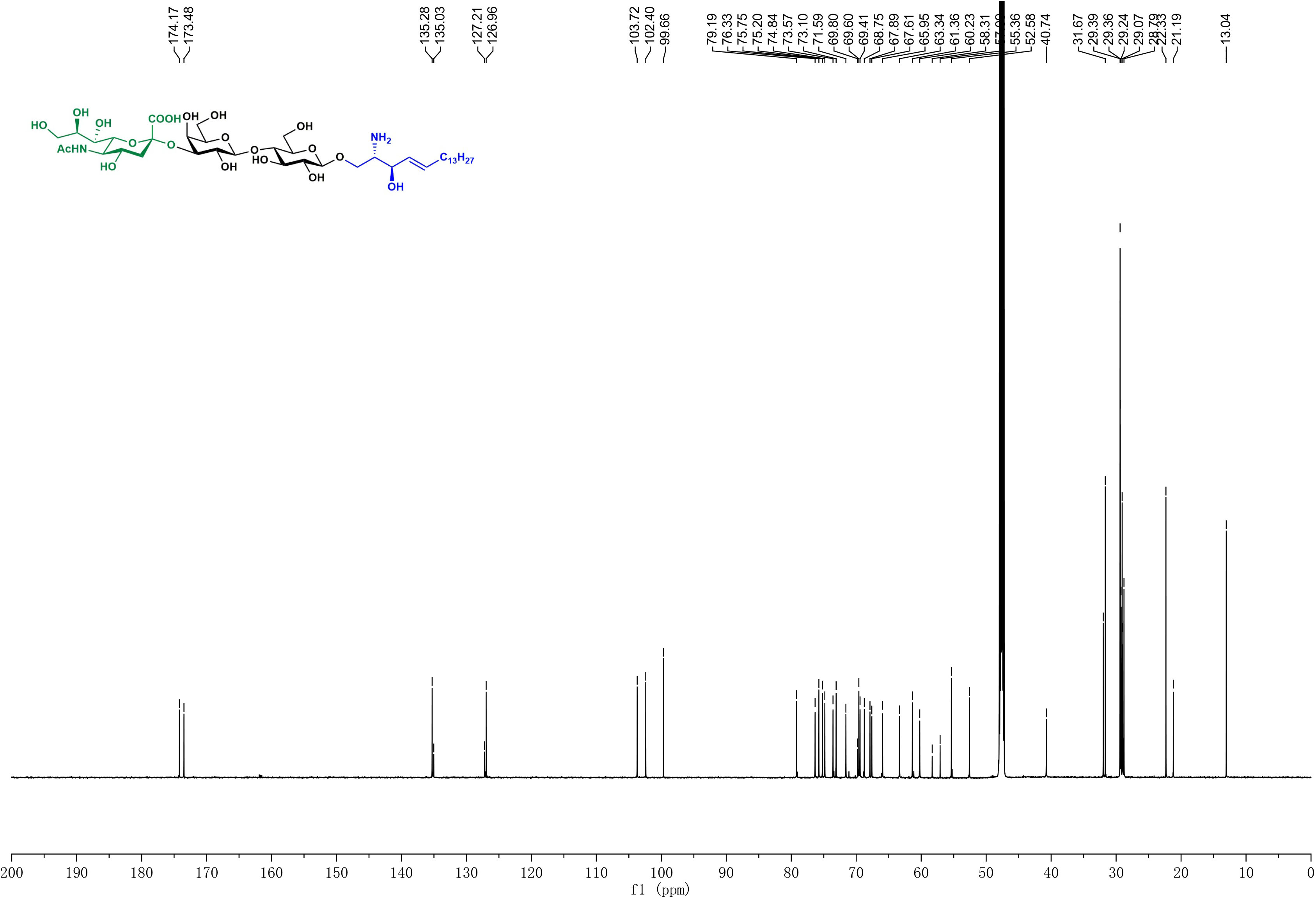


^1^H and ^13^C NMR spectra of GM2βSph **(7)**


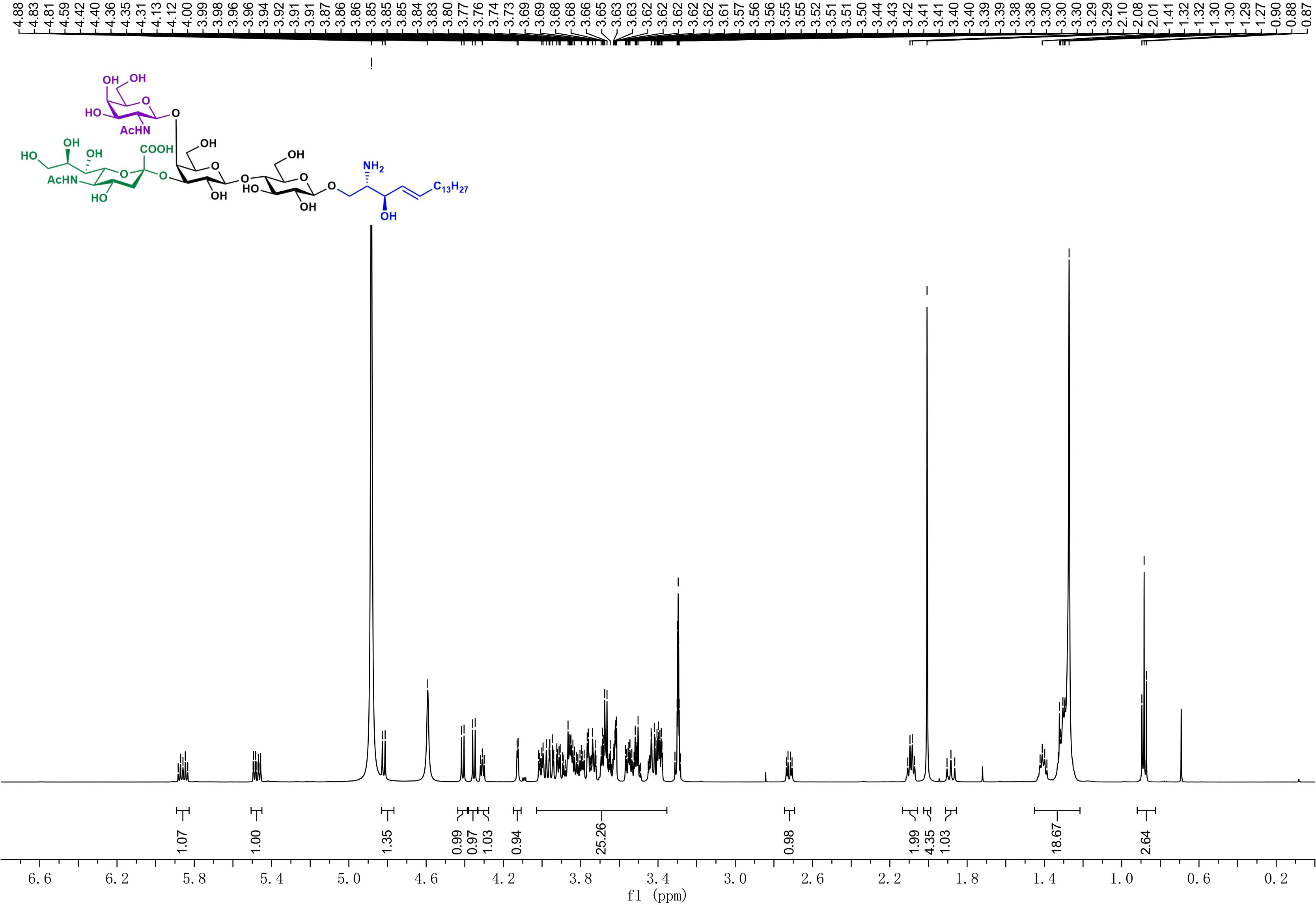


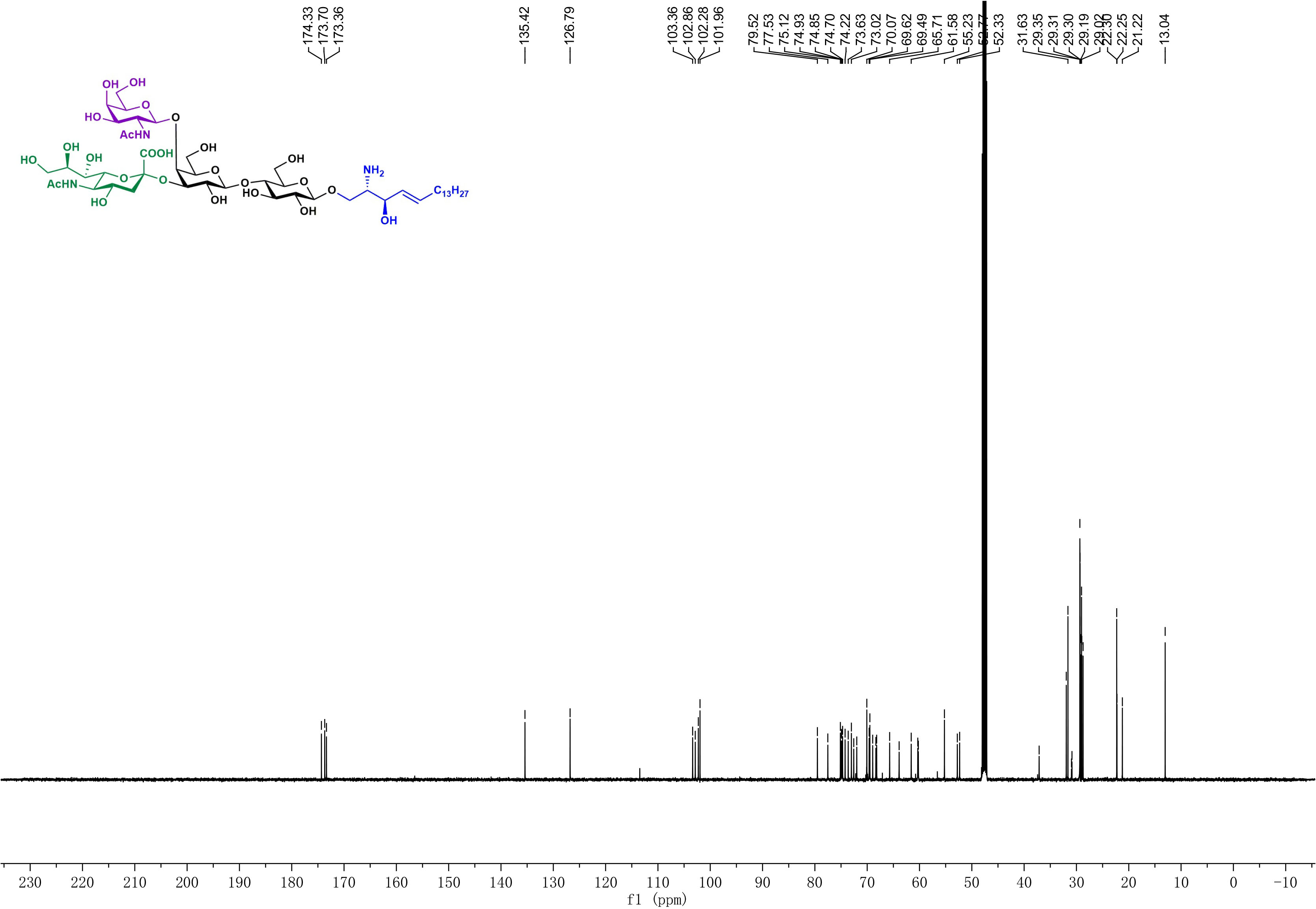


^1^H and ^13^C NMR spectra of GM1βSph **(8)**


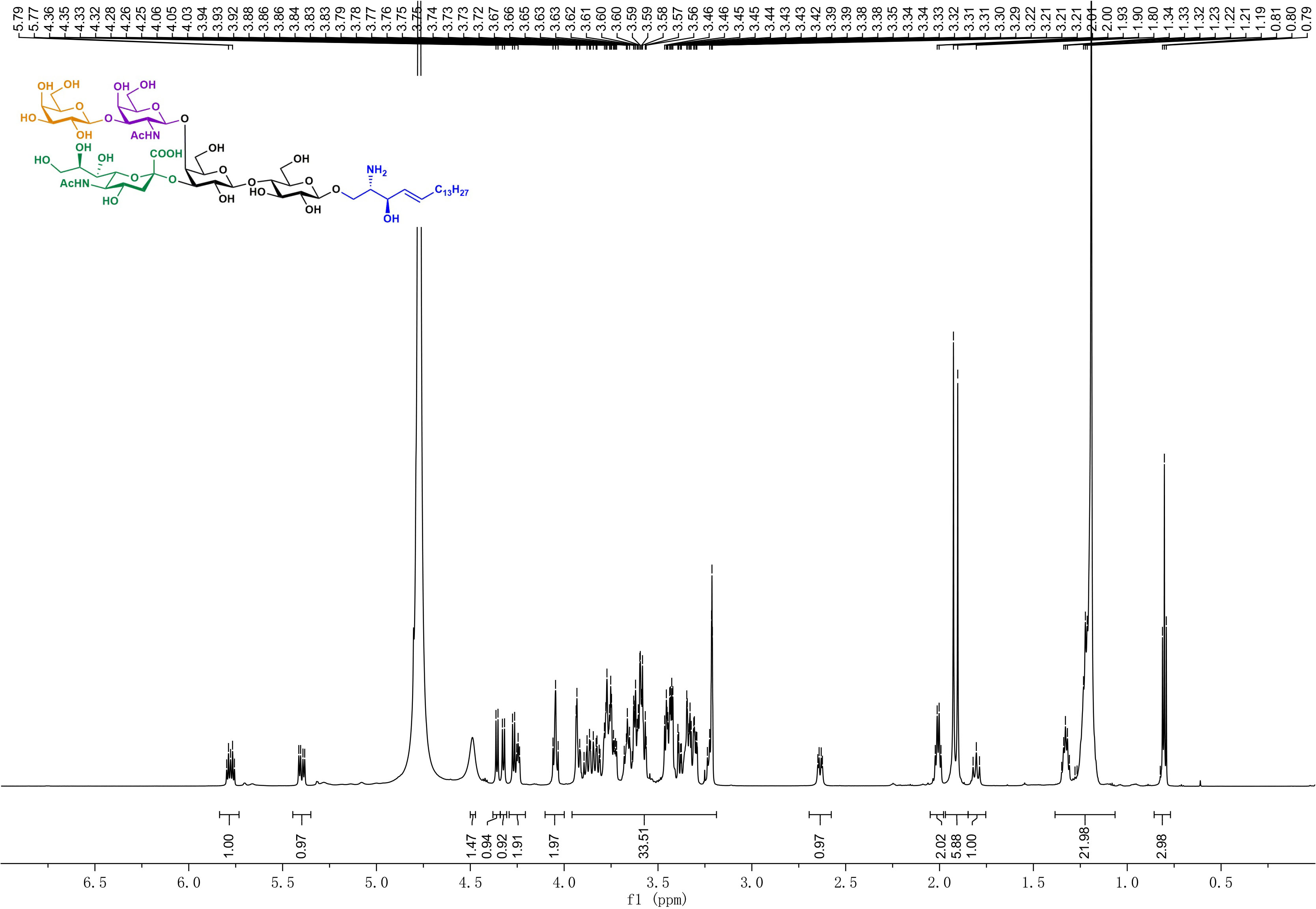


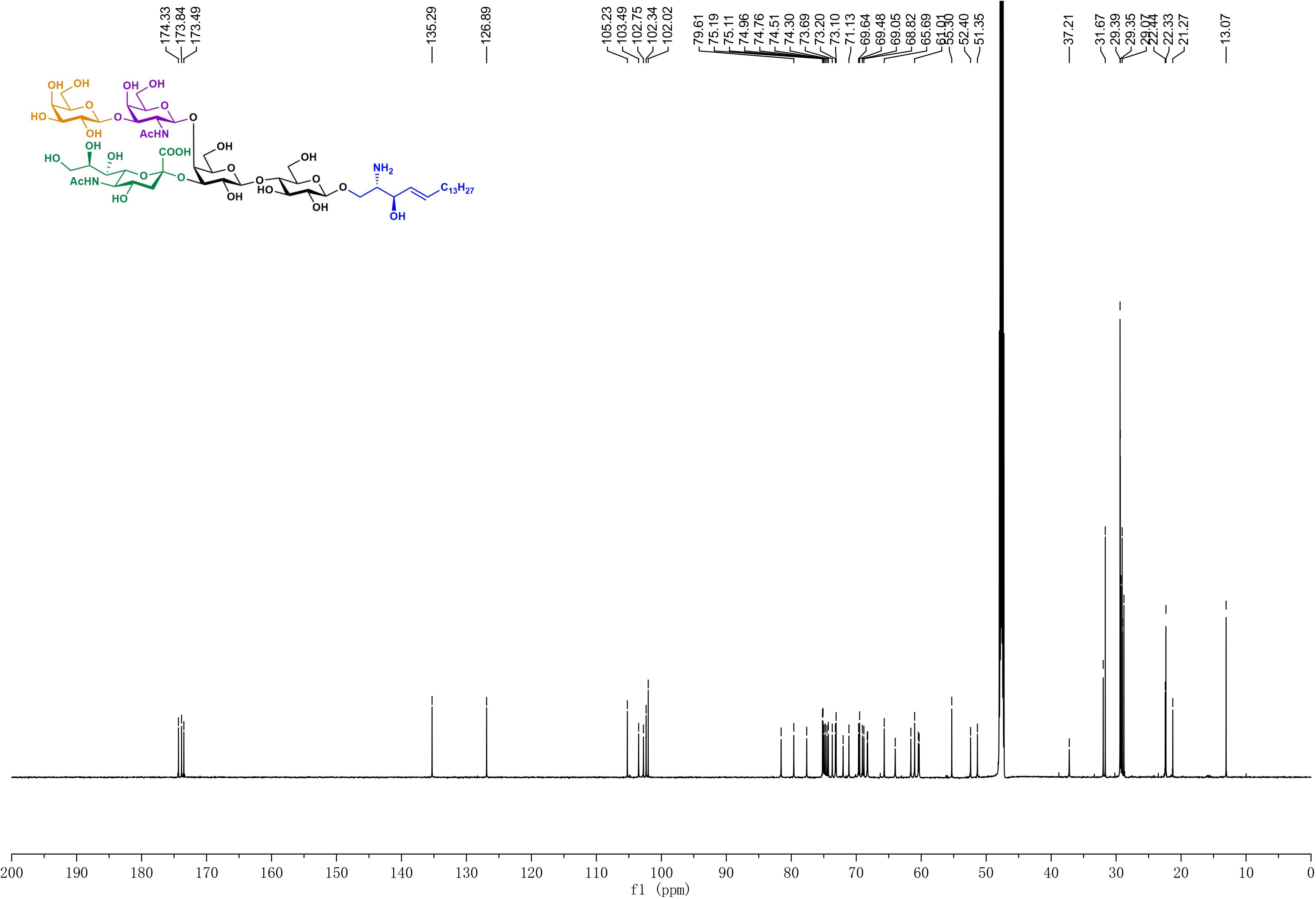


^1^H and ^13^C NMR spectra of GD3βSph **(9)**


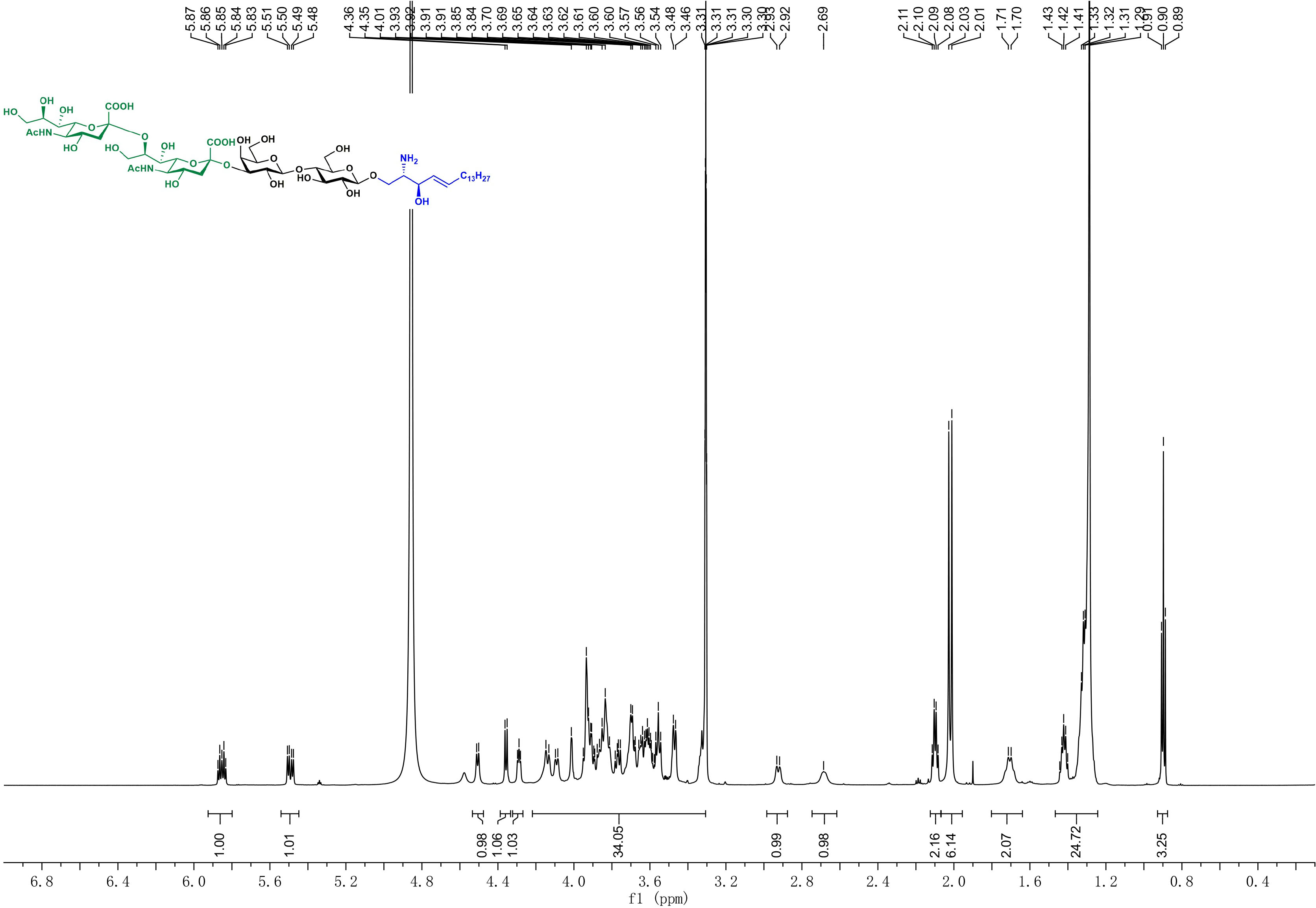


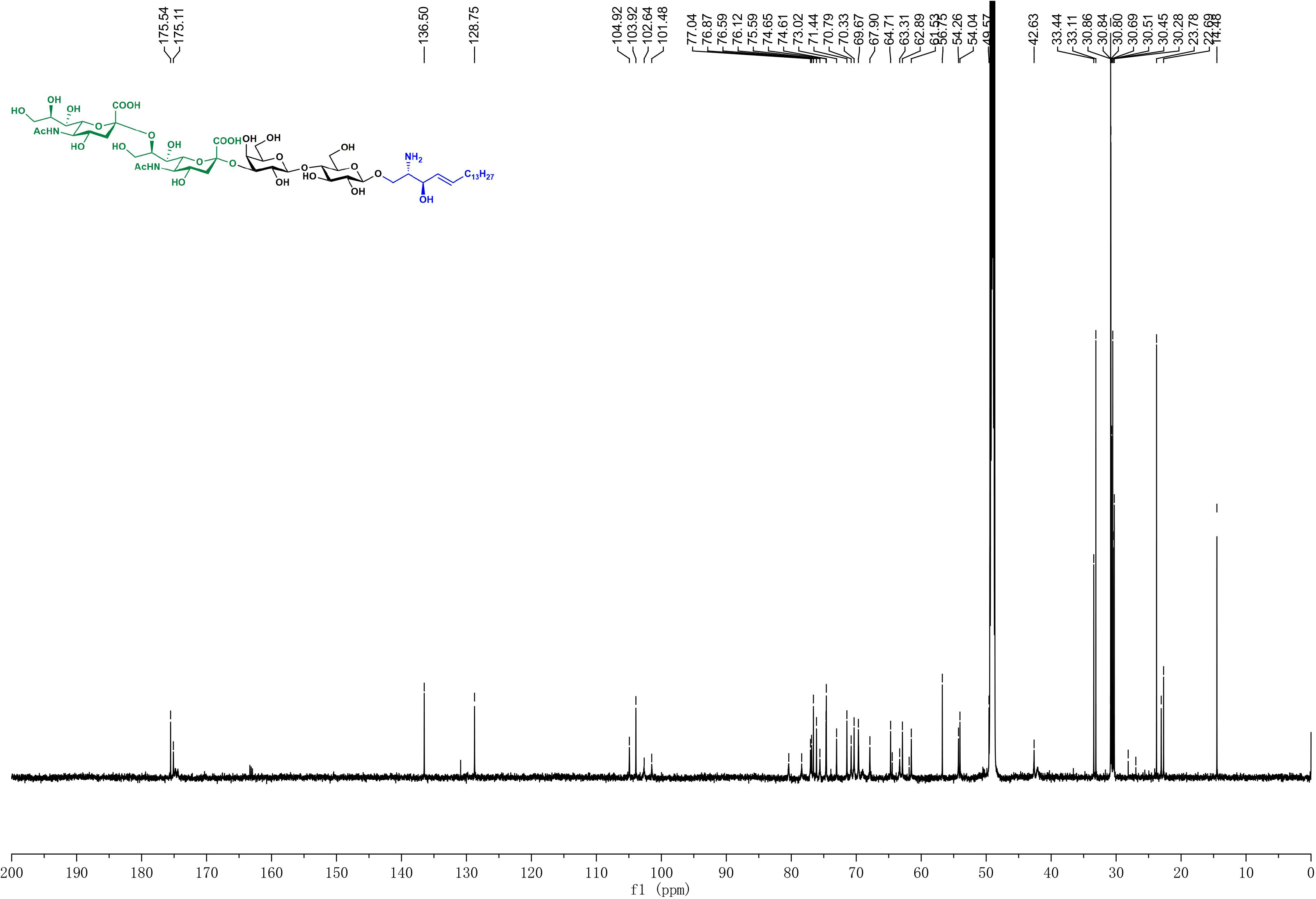


^1^H and ^13^C NMR spectra of GD2βSph **(10)**


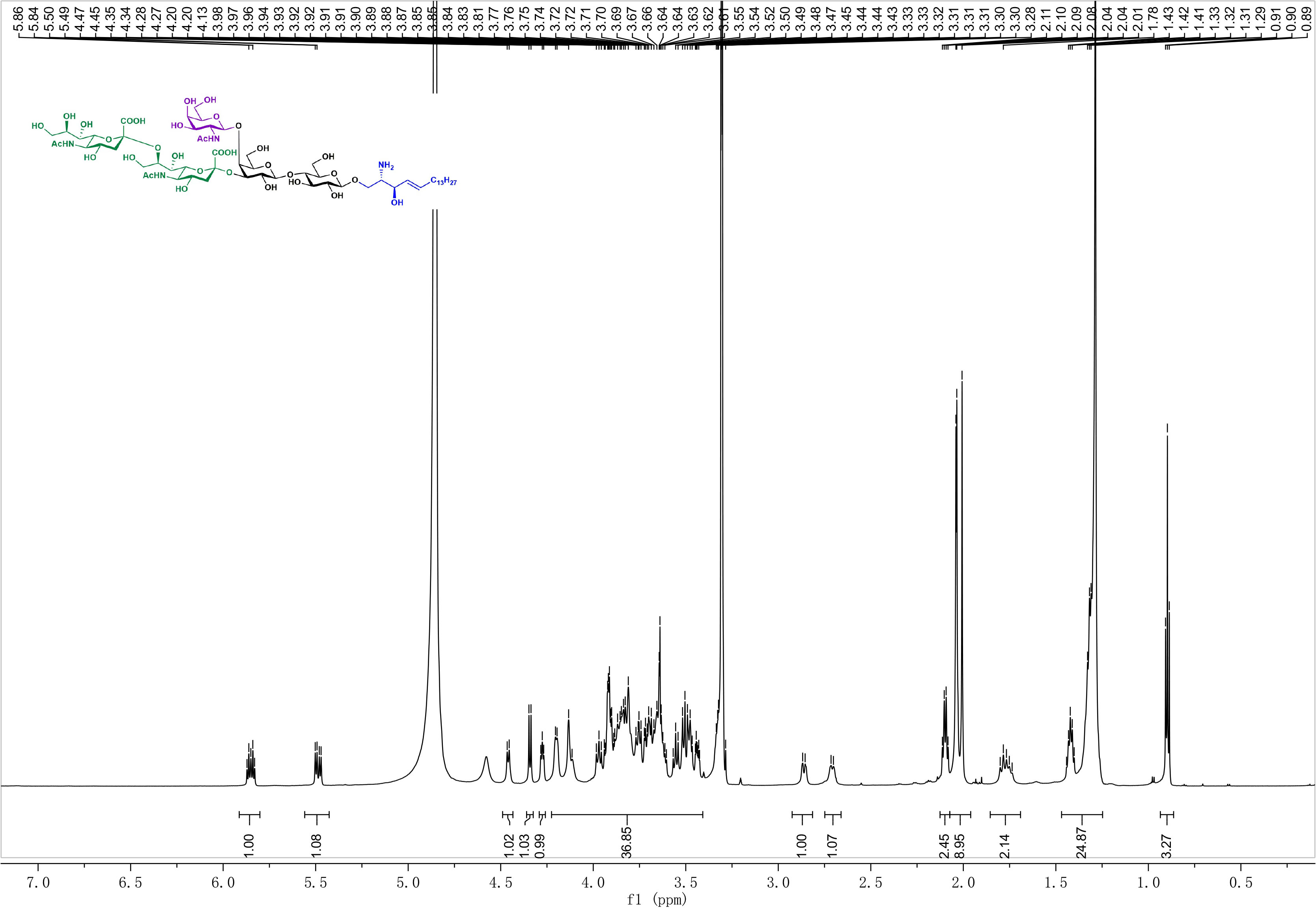


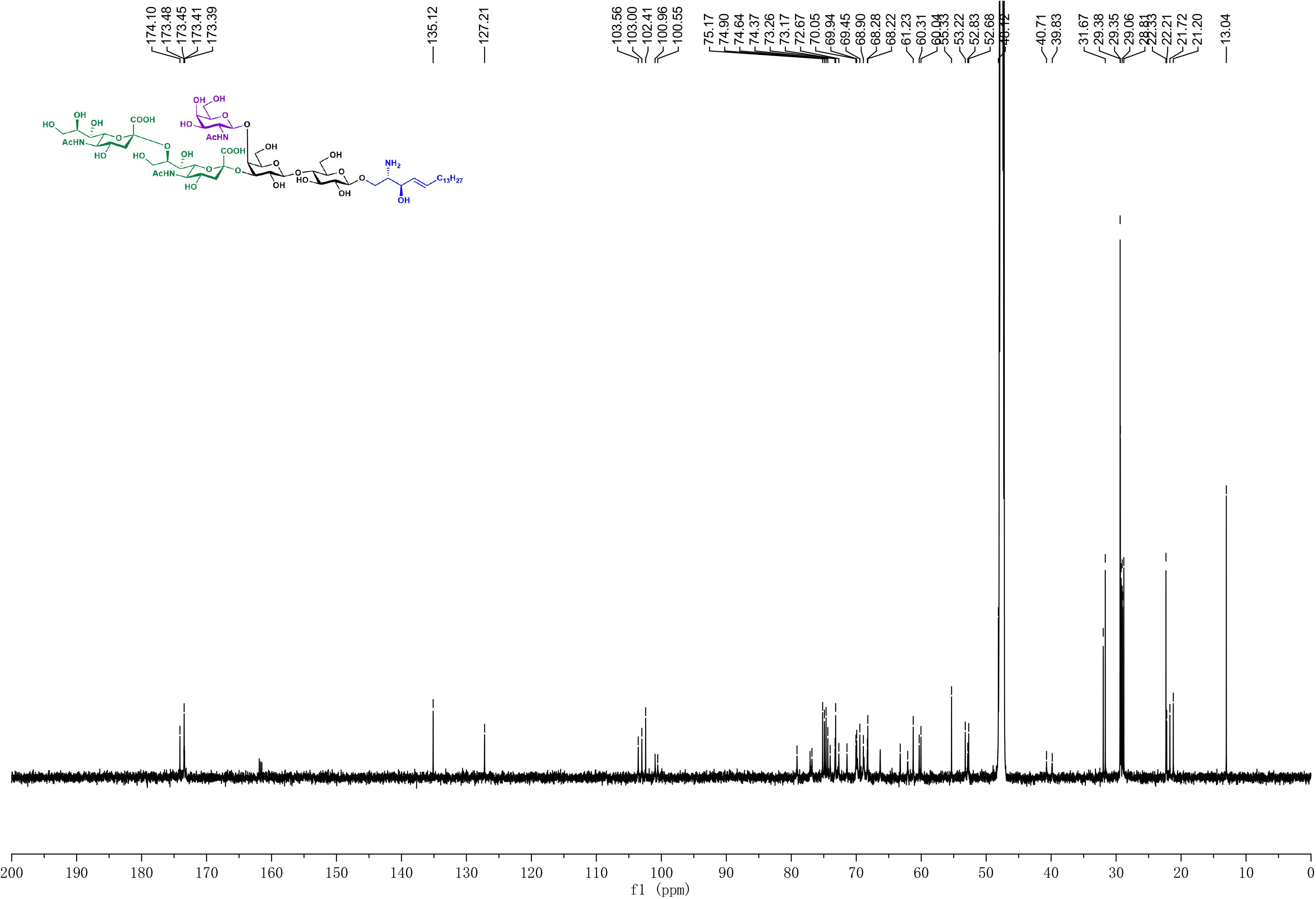


^1^H and ^13^C NMR spectra of GM3 **(1)**


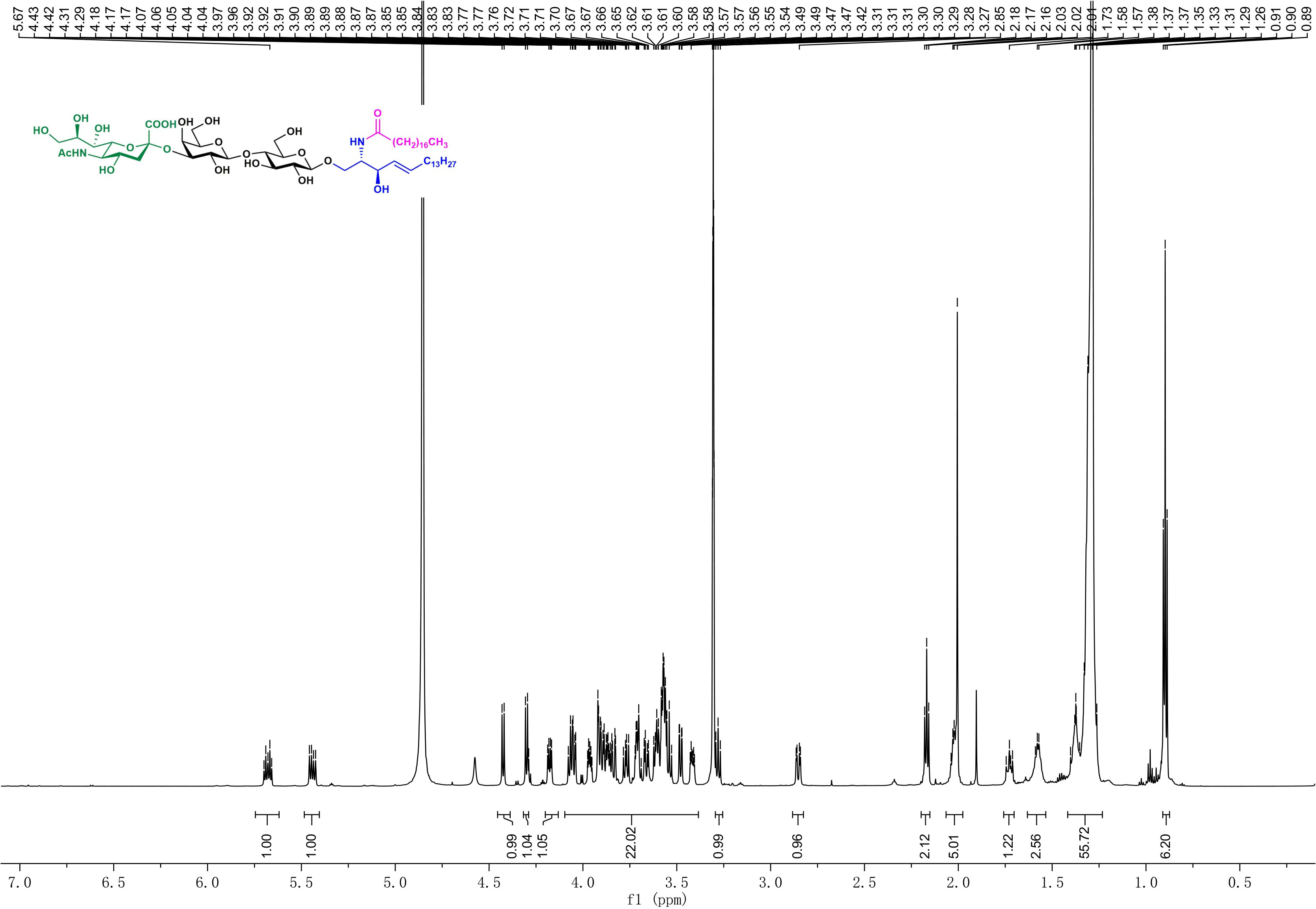


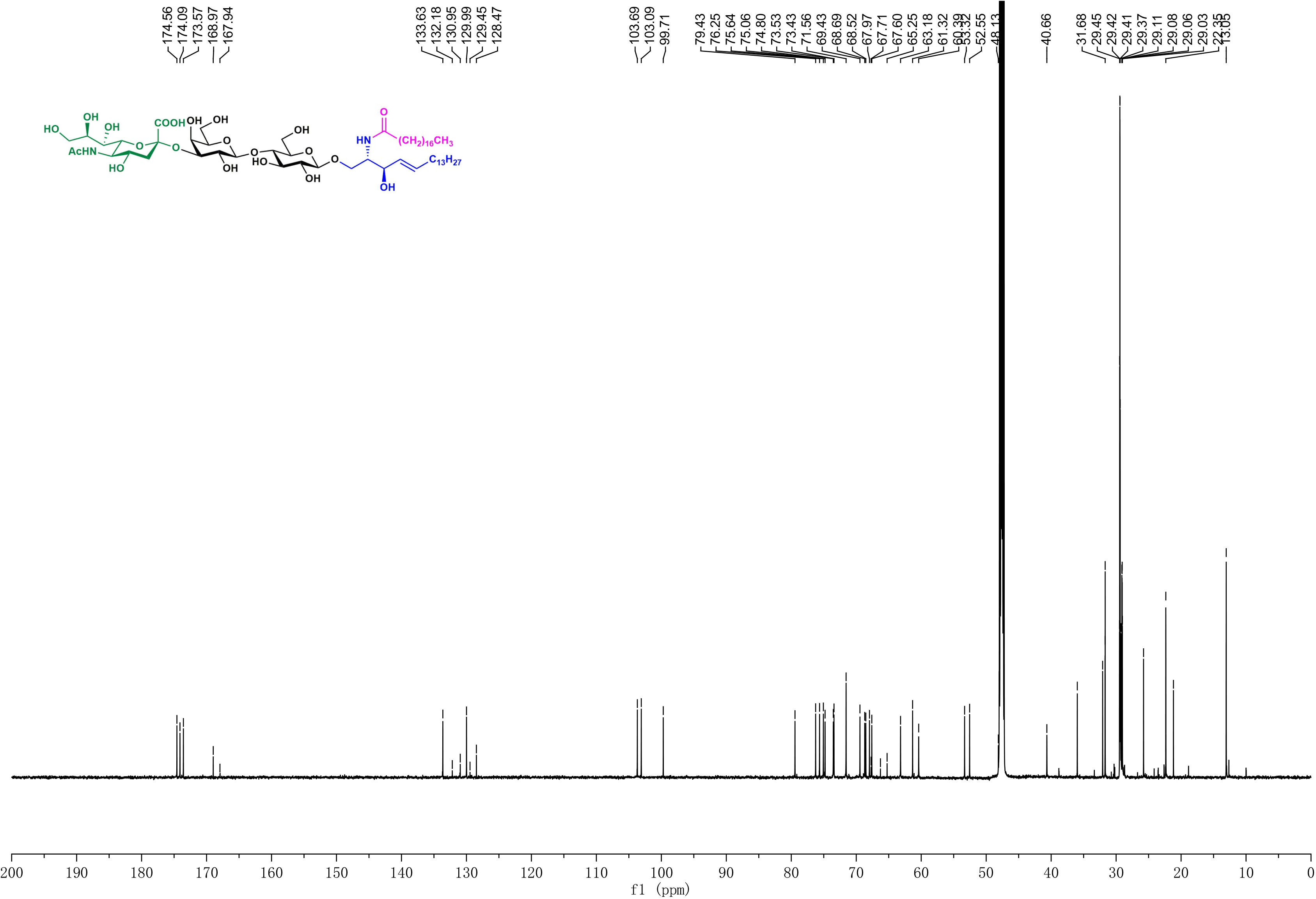


^1^H and ^13^C NMR spectra of GM2 **(2)**


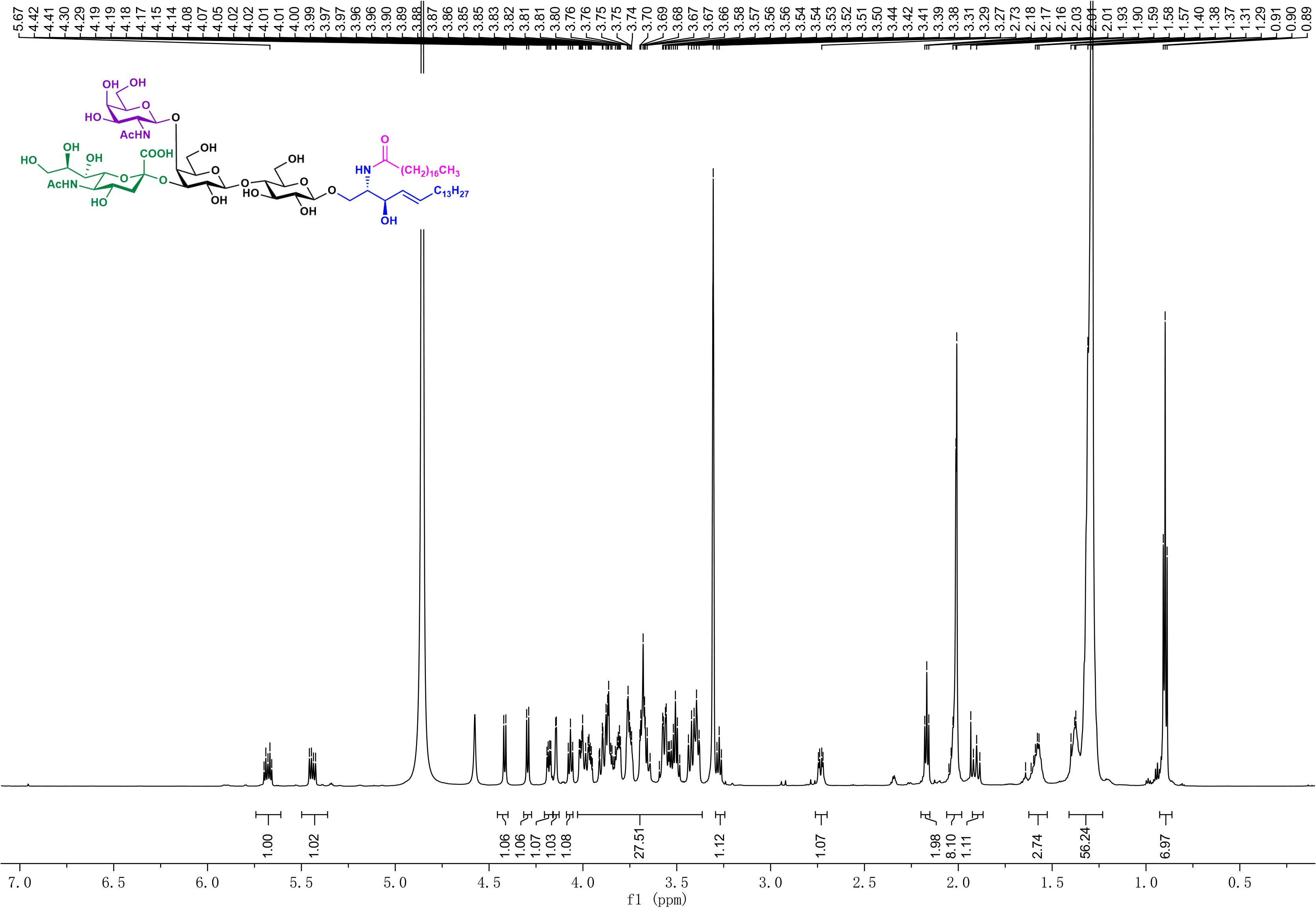


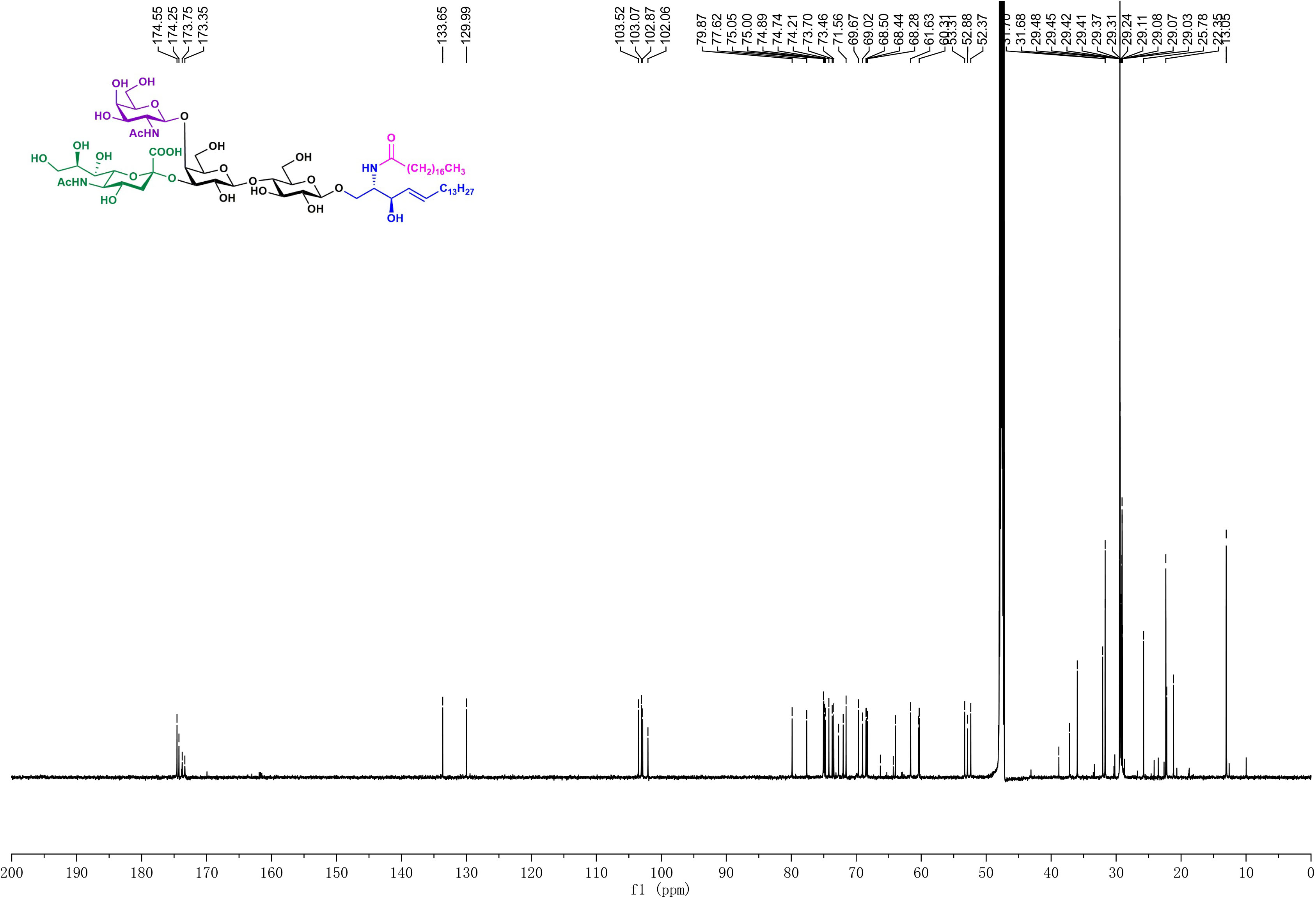


^1^H and ^13^C NMR spectra of GM1 **(3)**


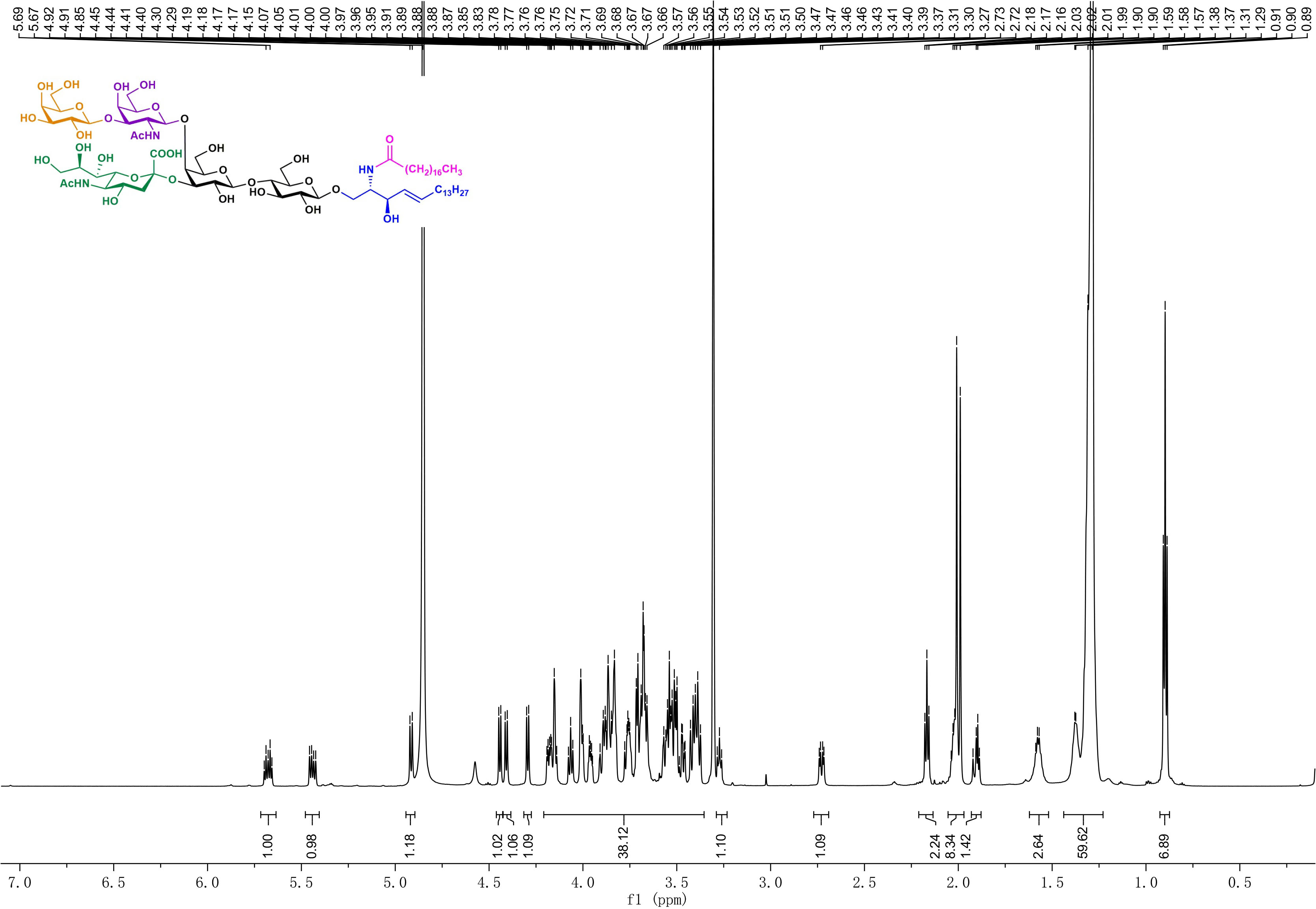


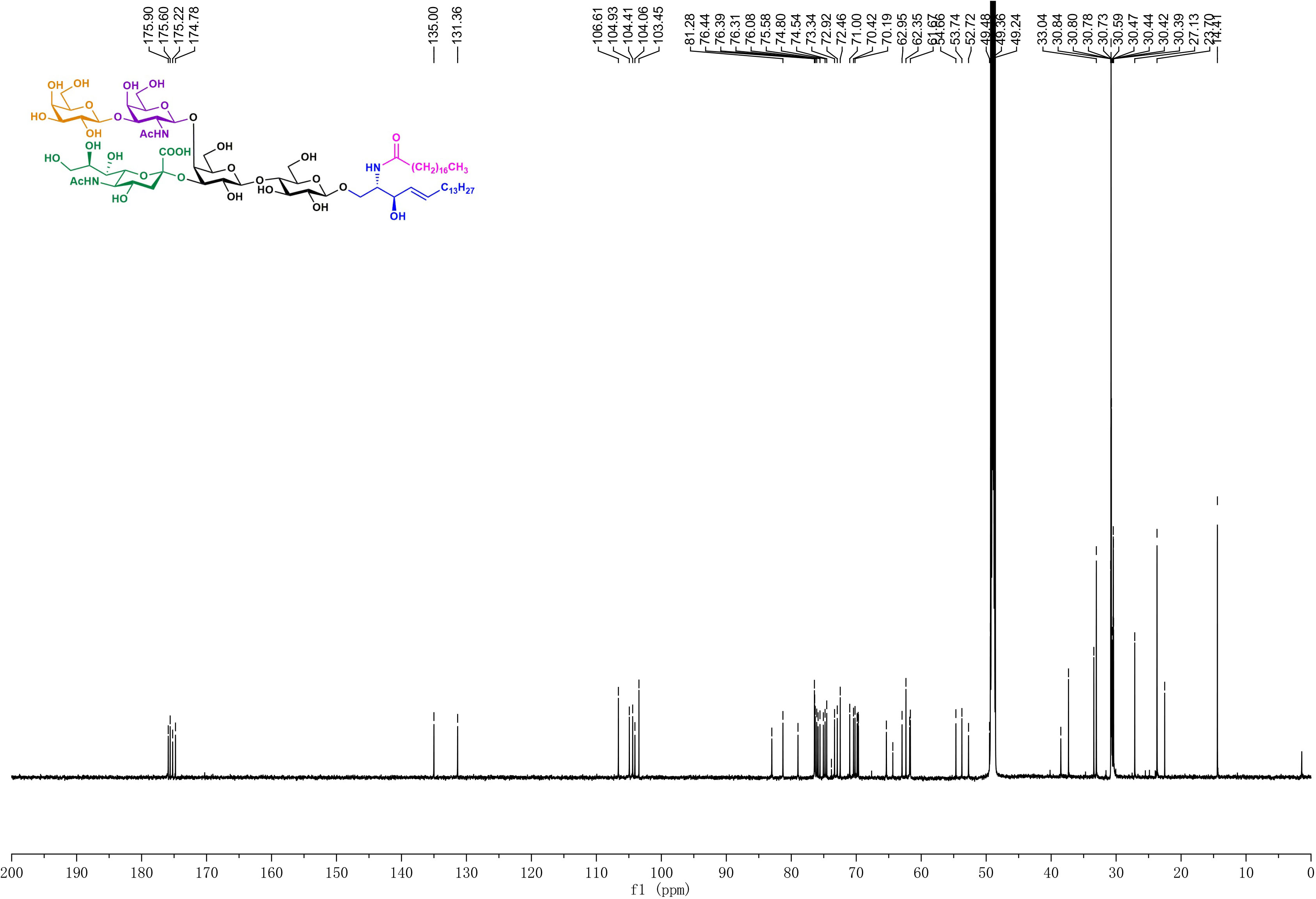


^1^H and ^13^C NMR spectra of GD3 **(4)**

^
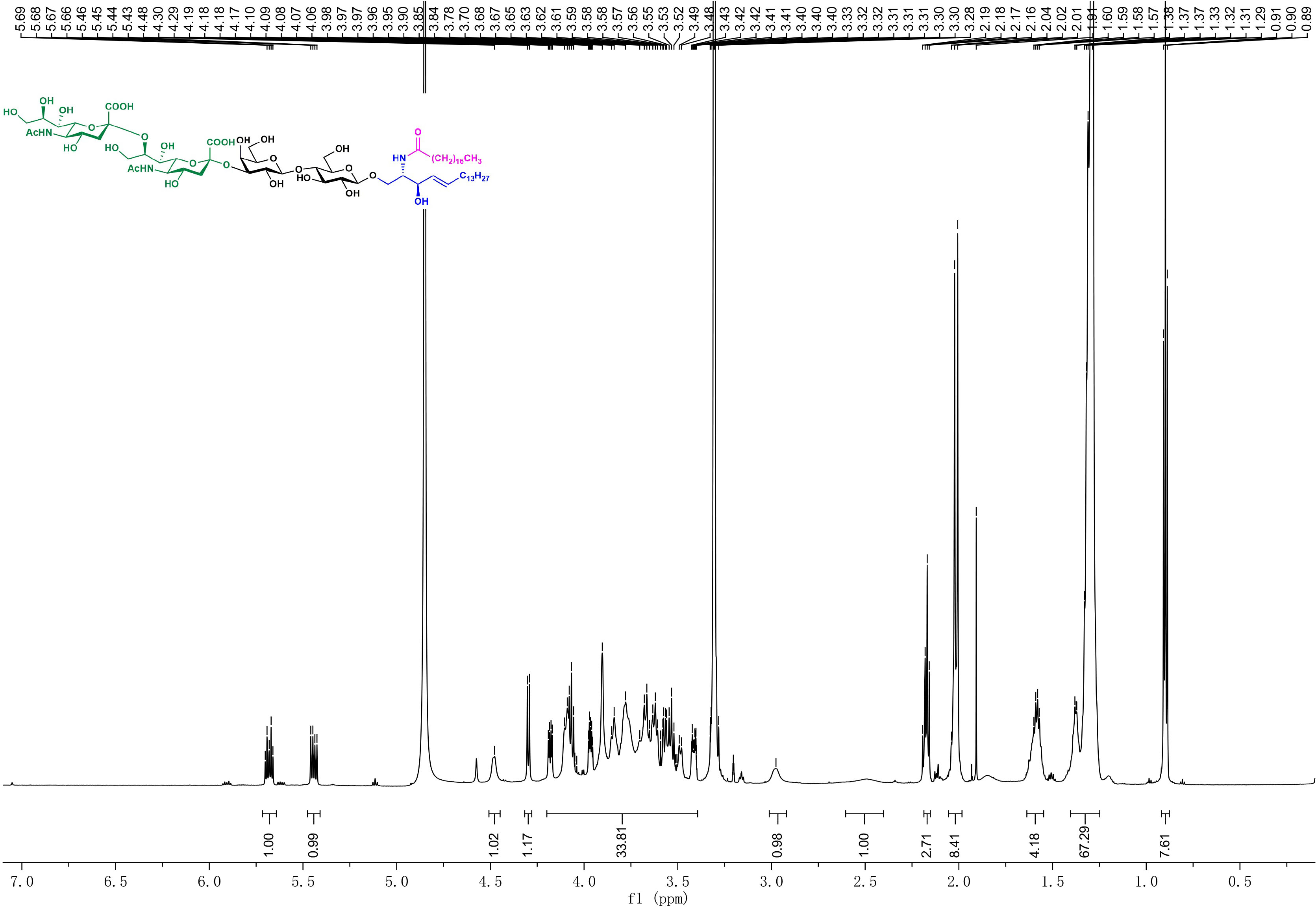
^

^
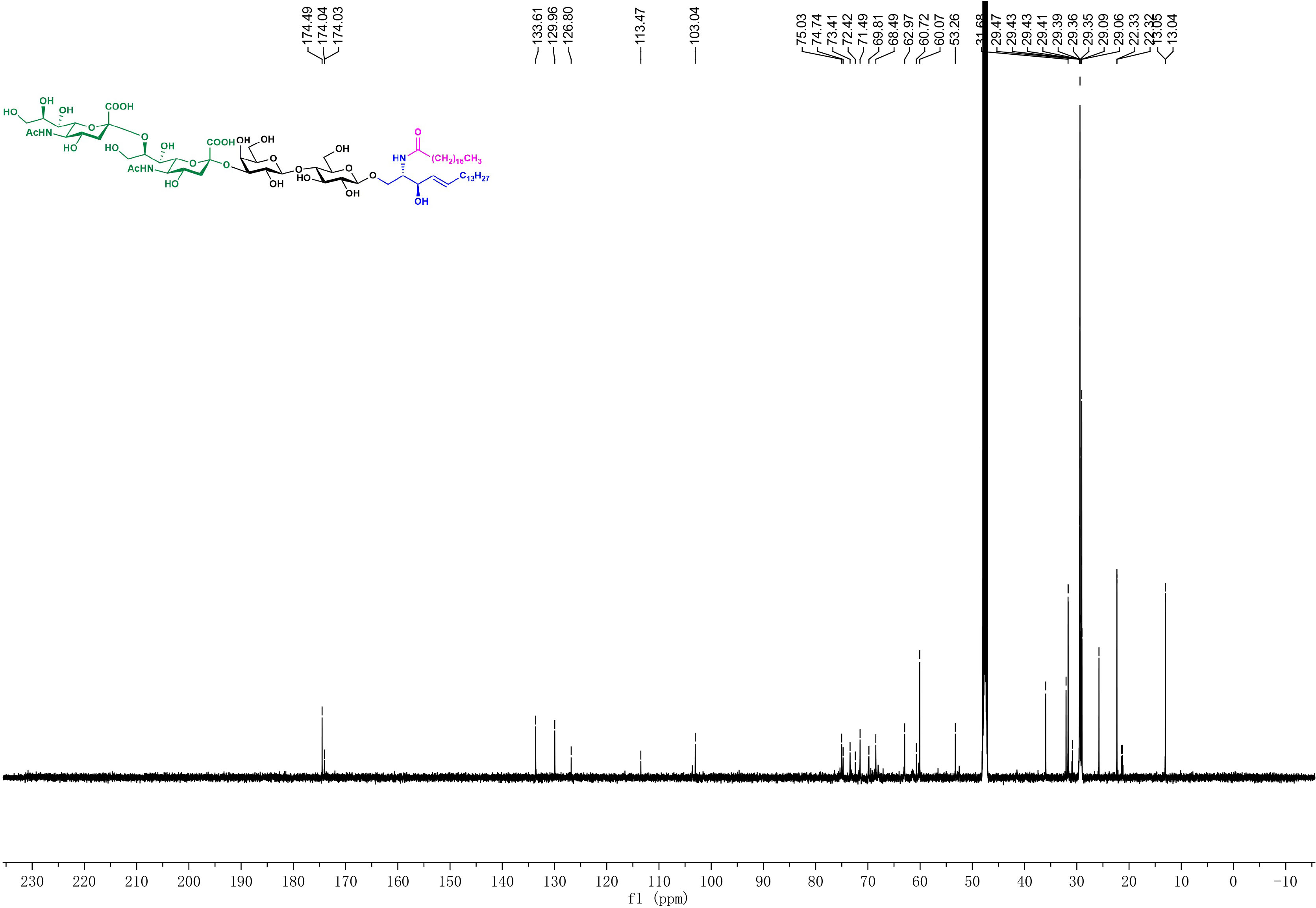
^

^1^H and ^13^C NMR spectra of GD2 **(5)**

^
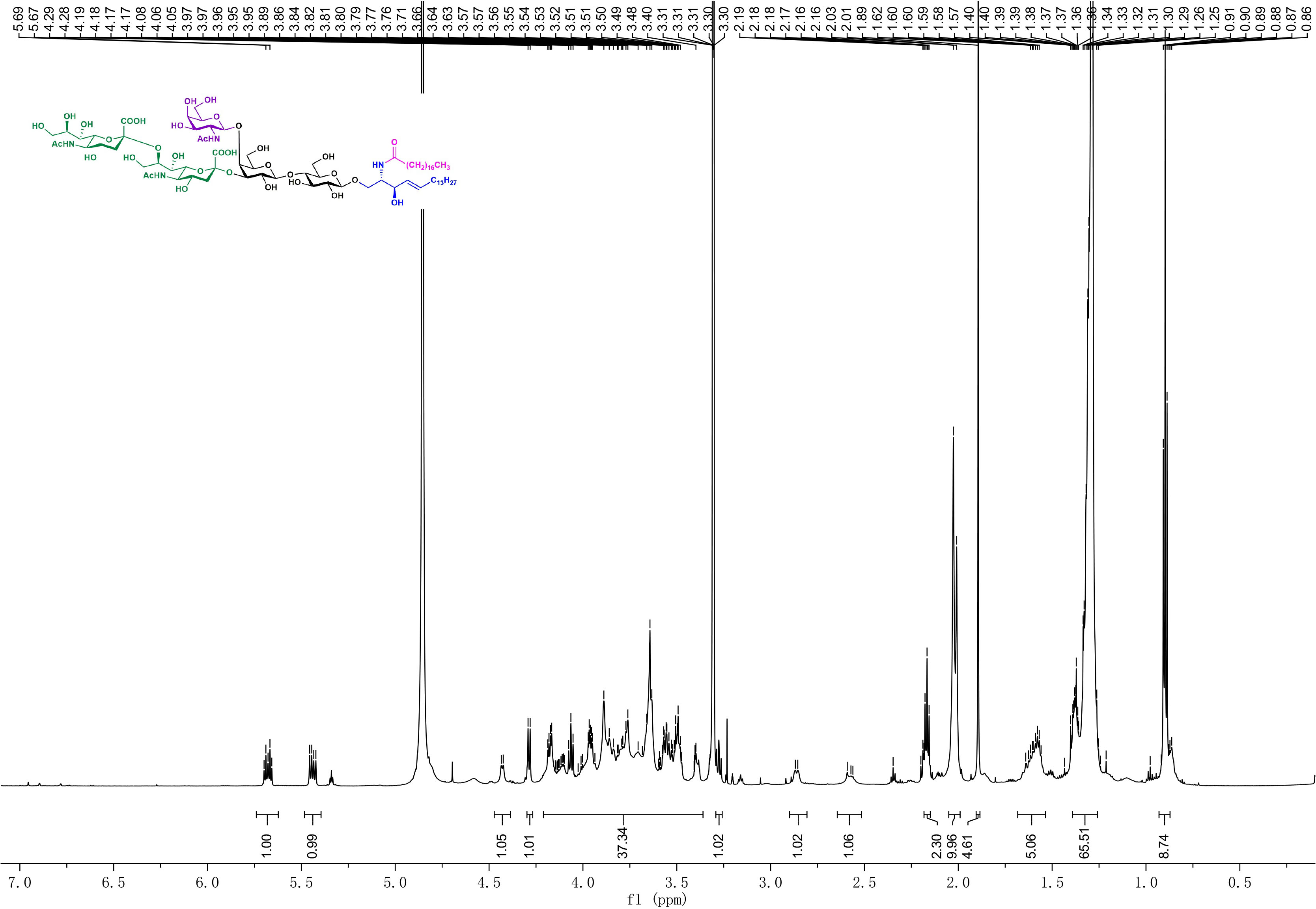
^


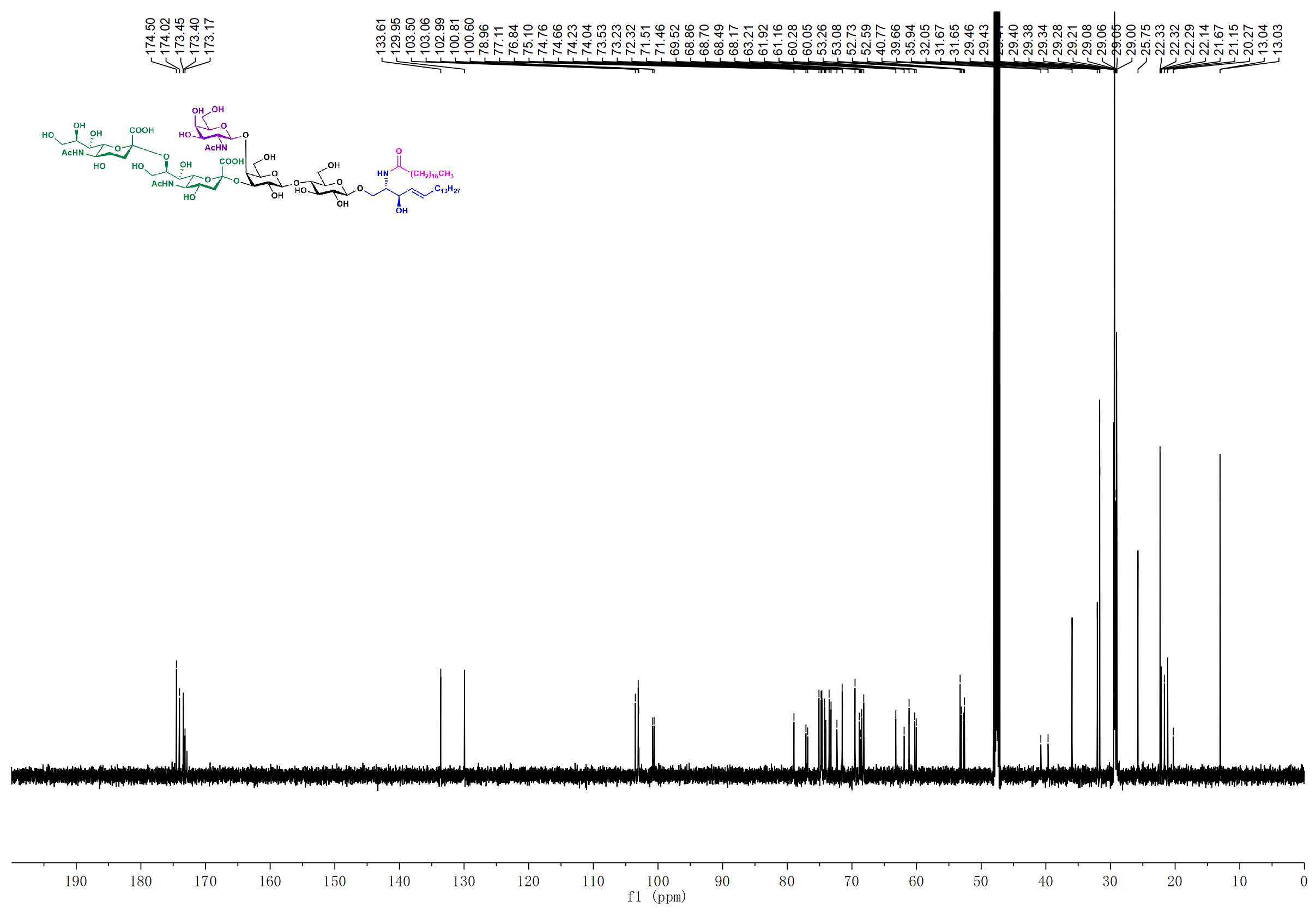


^1^H and ^13^C NMR spectra of GM1βSph (d18:1) **(8)**

^
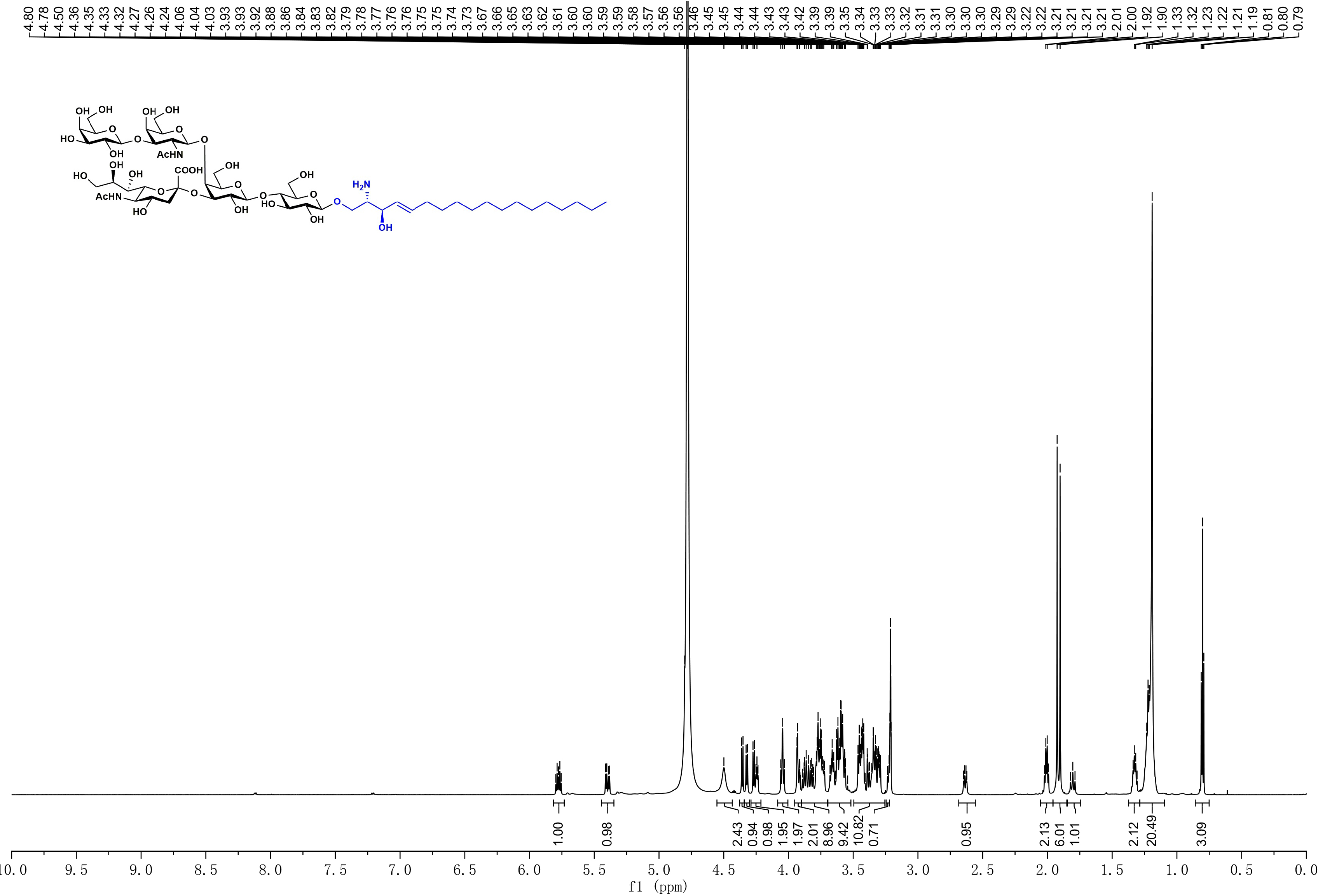
^

^
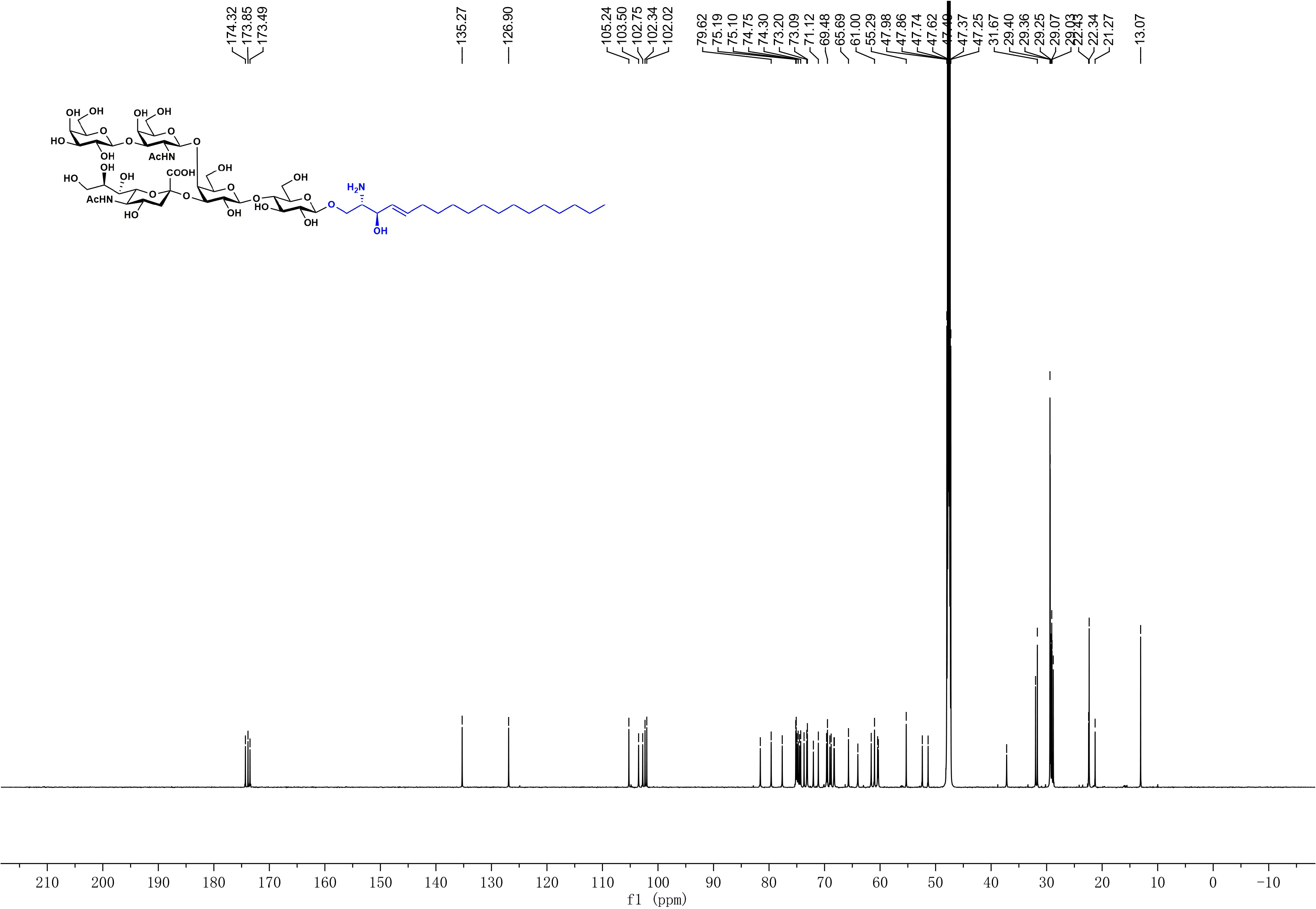
^

^1^H and ^13^C NMR spectra of GM1βSph (d20:1) **(18)**

^
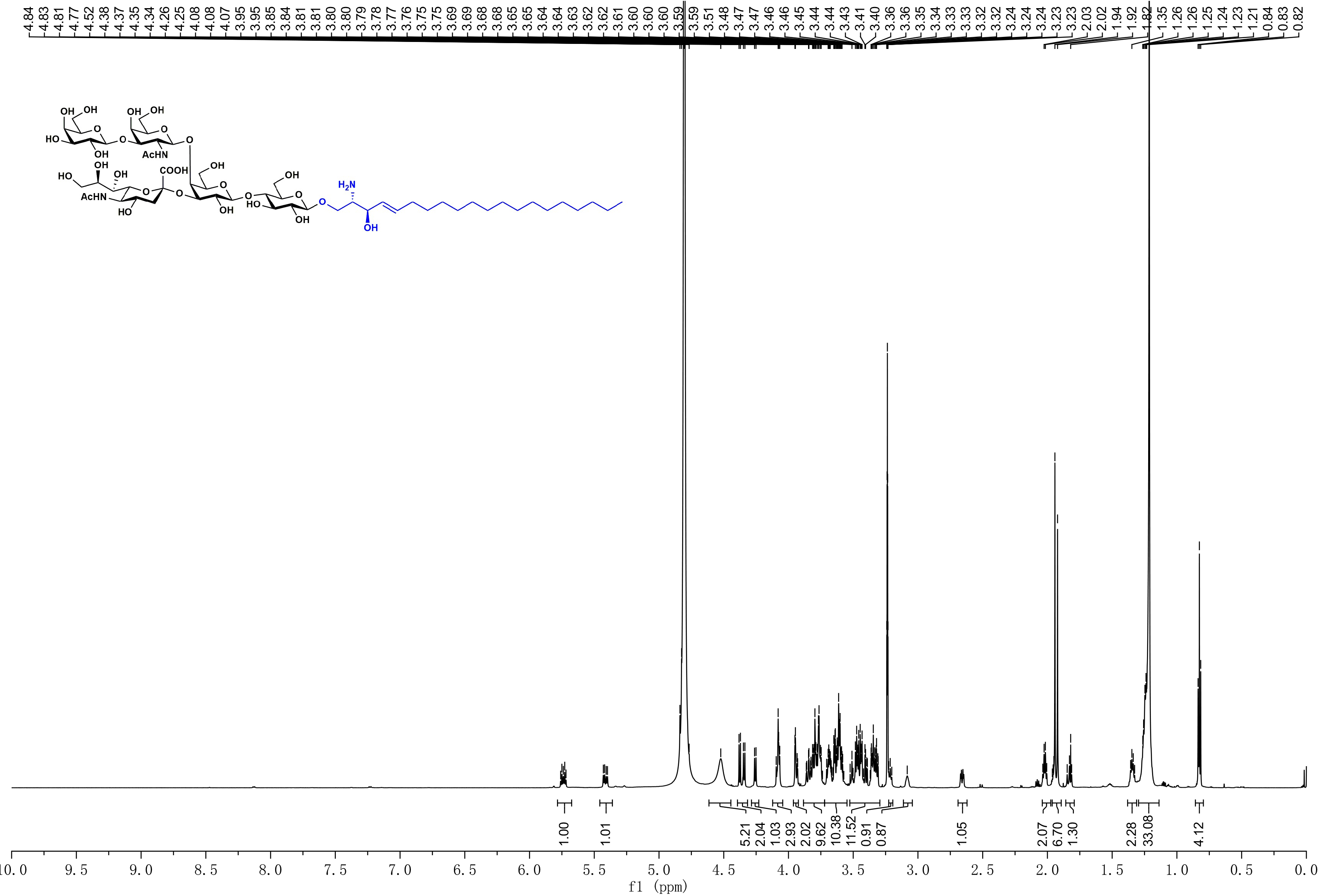
^

^
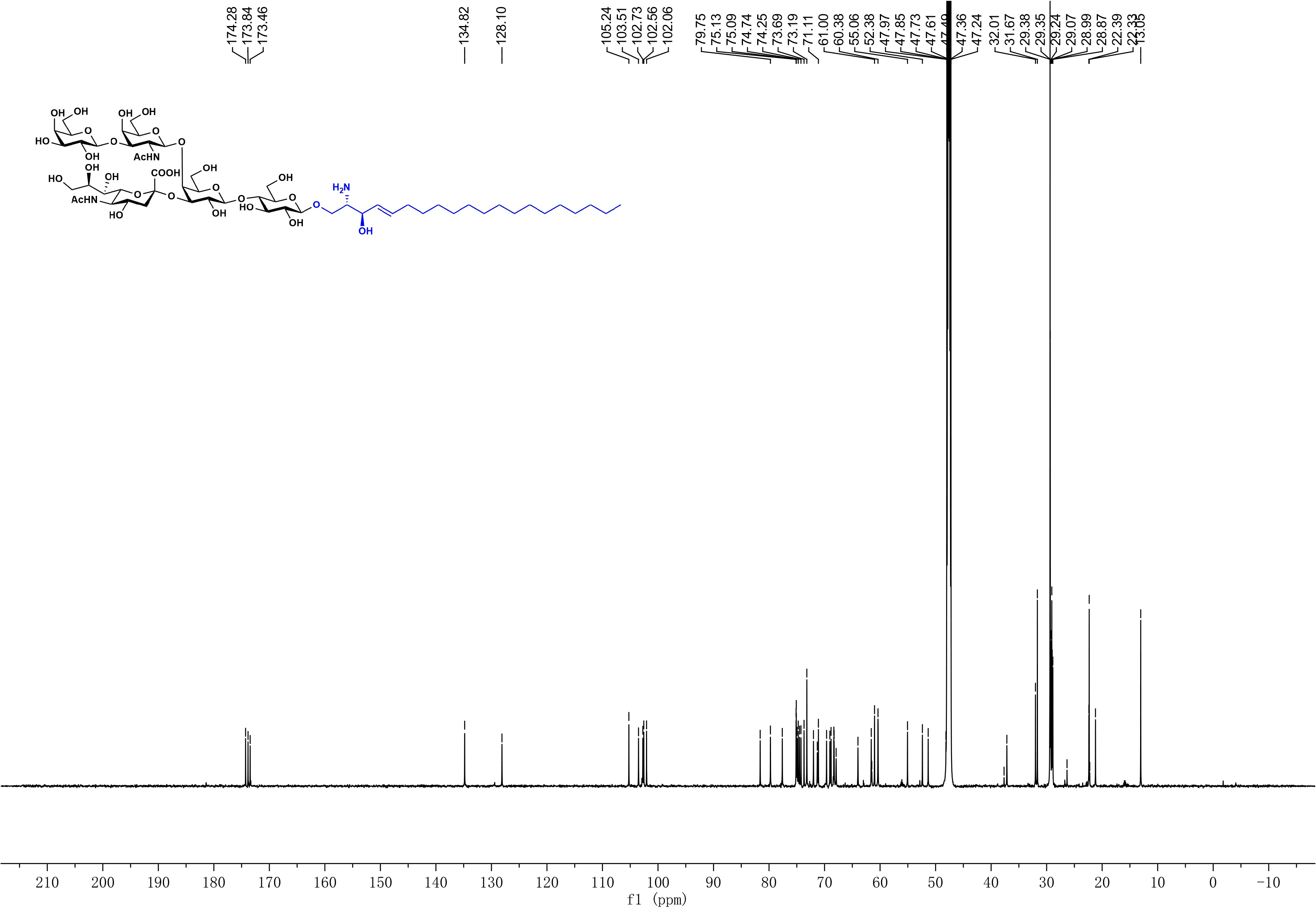
^

^1^H and ^13^C NMR spectra of GM1 (d18:1/C16:0) **(19)**


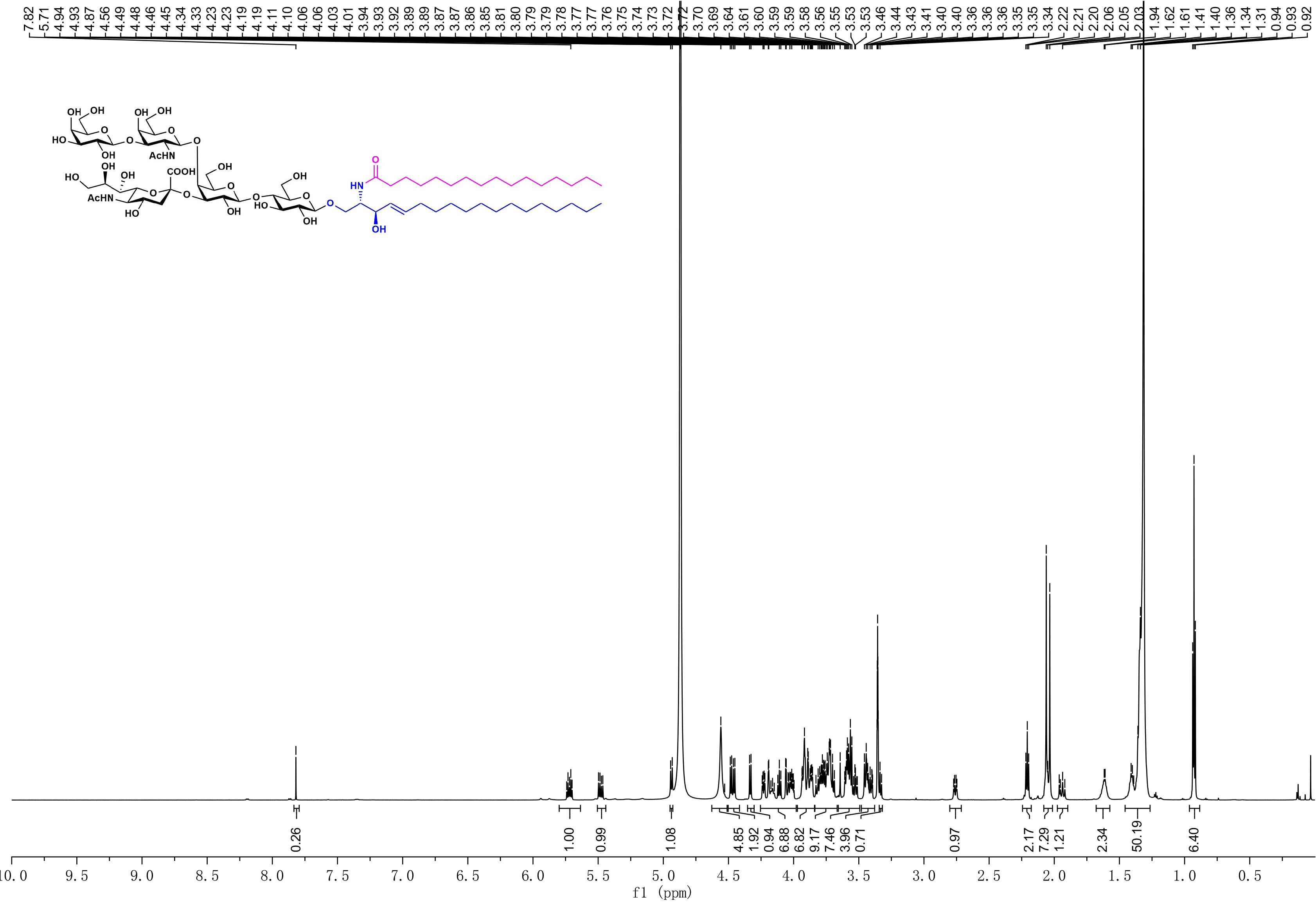


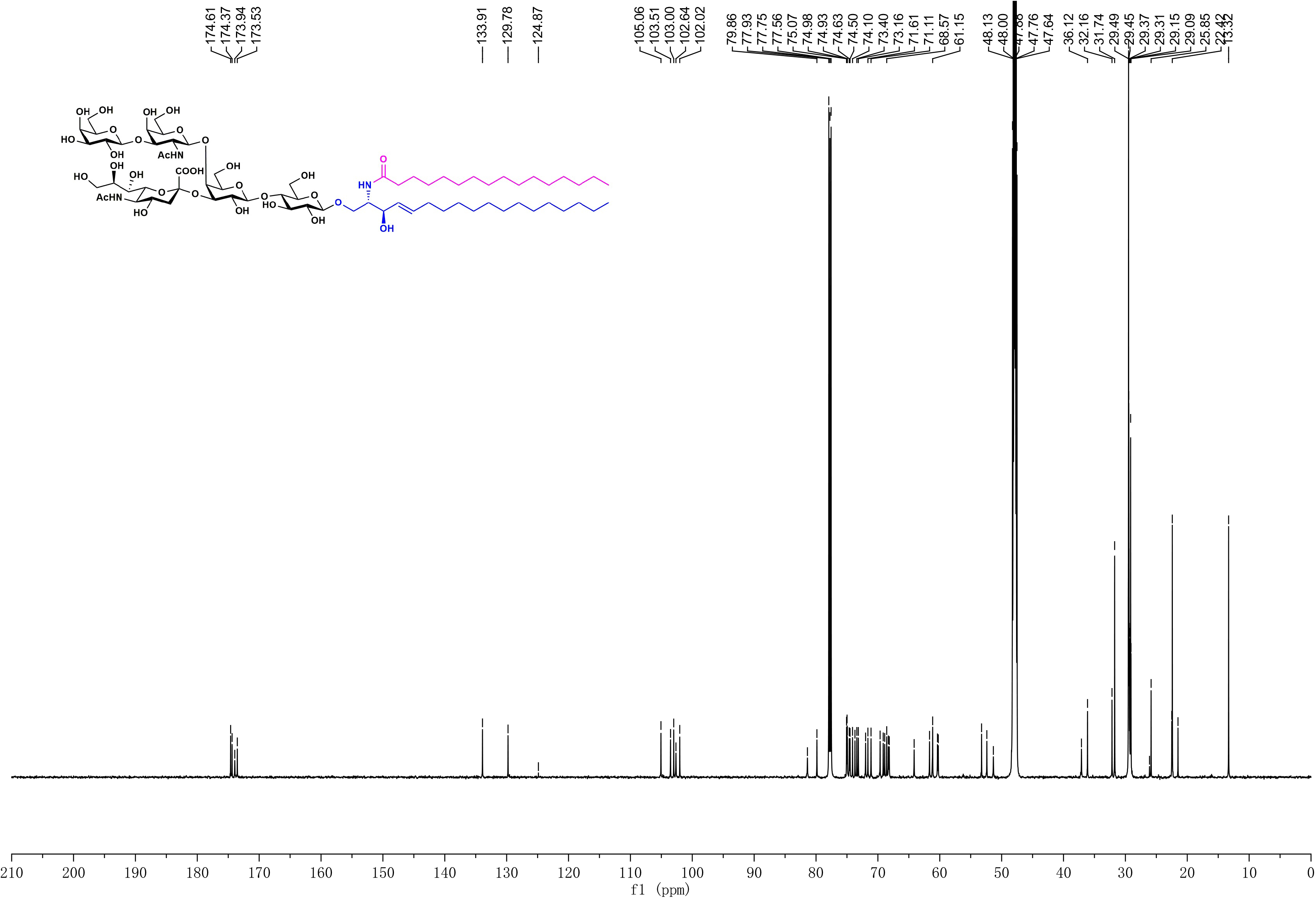


^1^H and ^13^C NMR spectra of GM1 (d18:1/C18:0) **(3)**


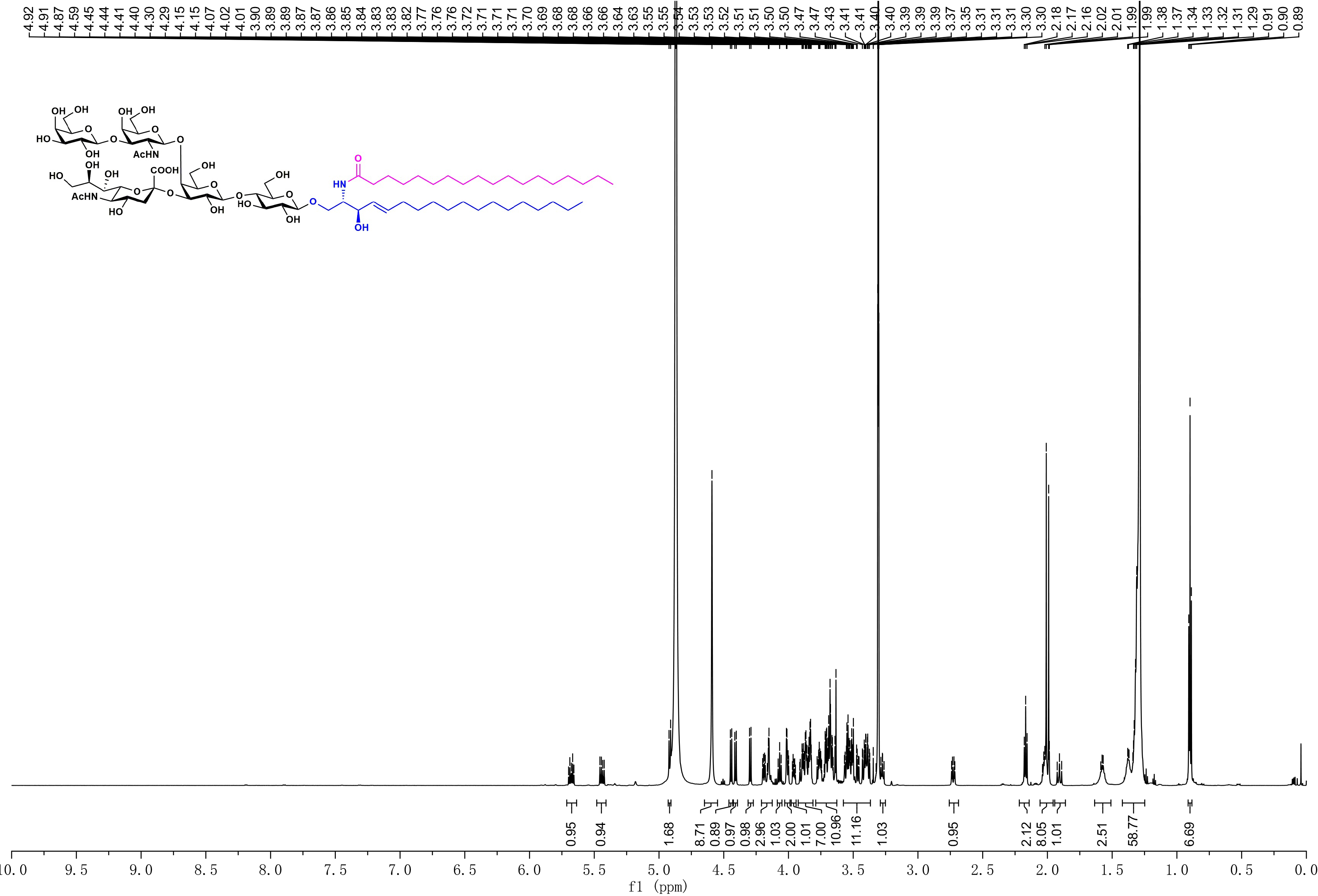


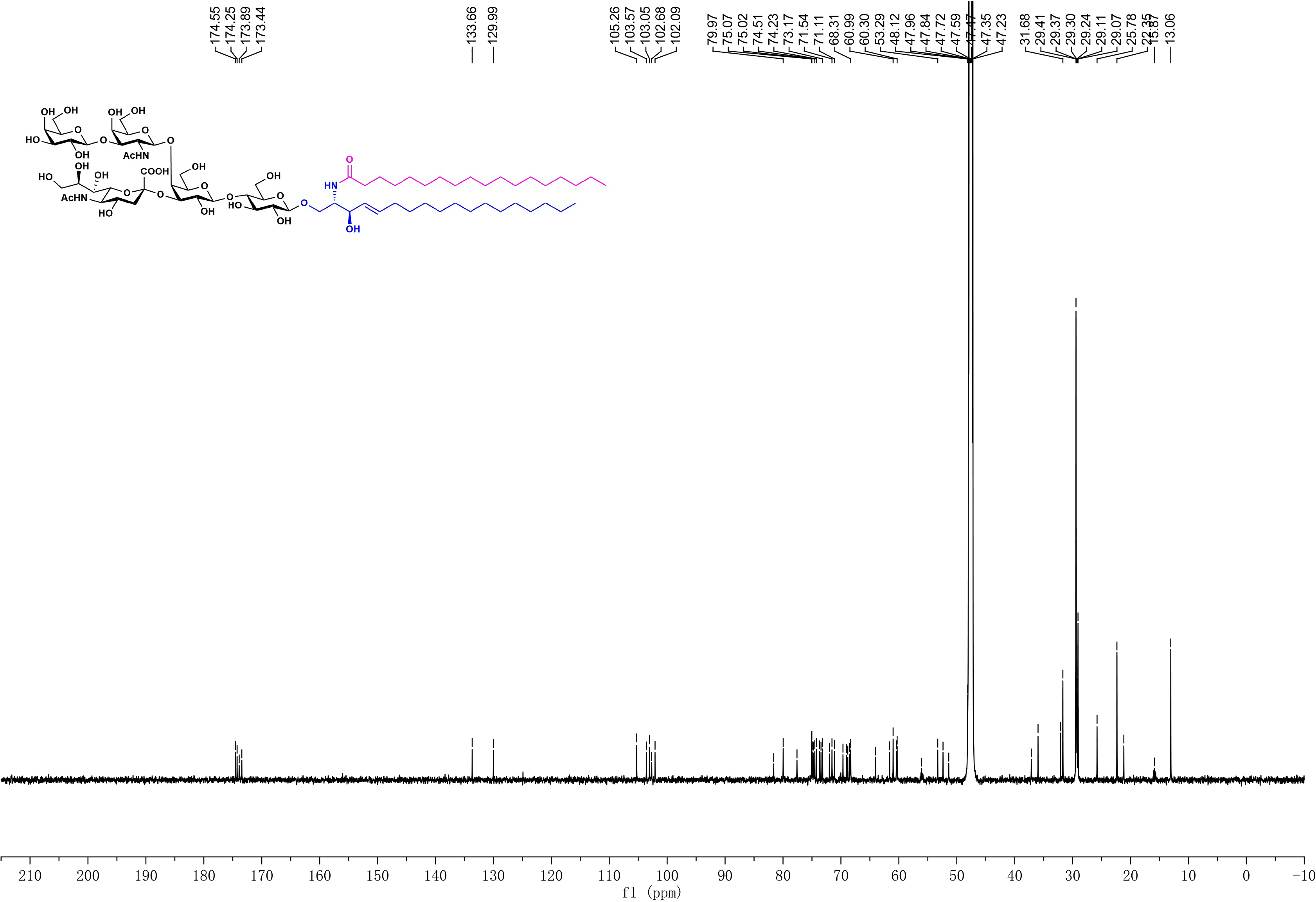


^1^H and ^13^C NMR spectra of GM1 (d18:1/C20:0) **(20)**


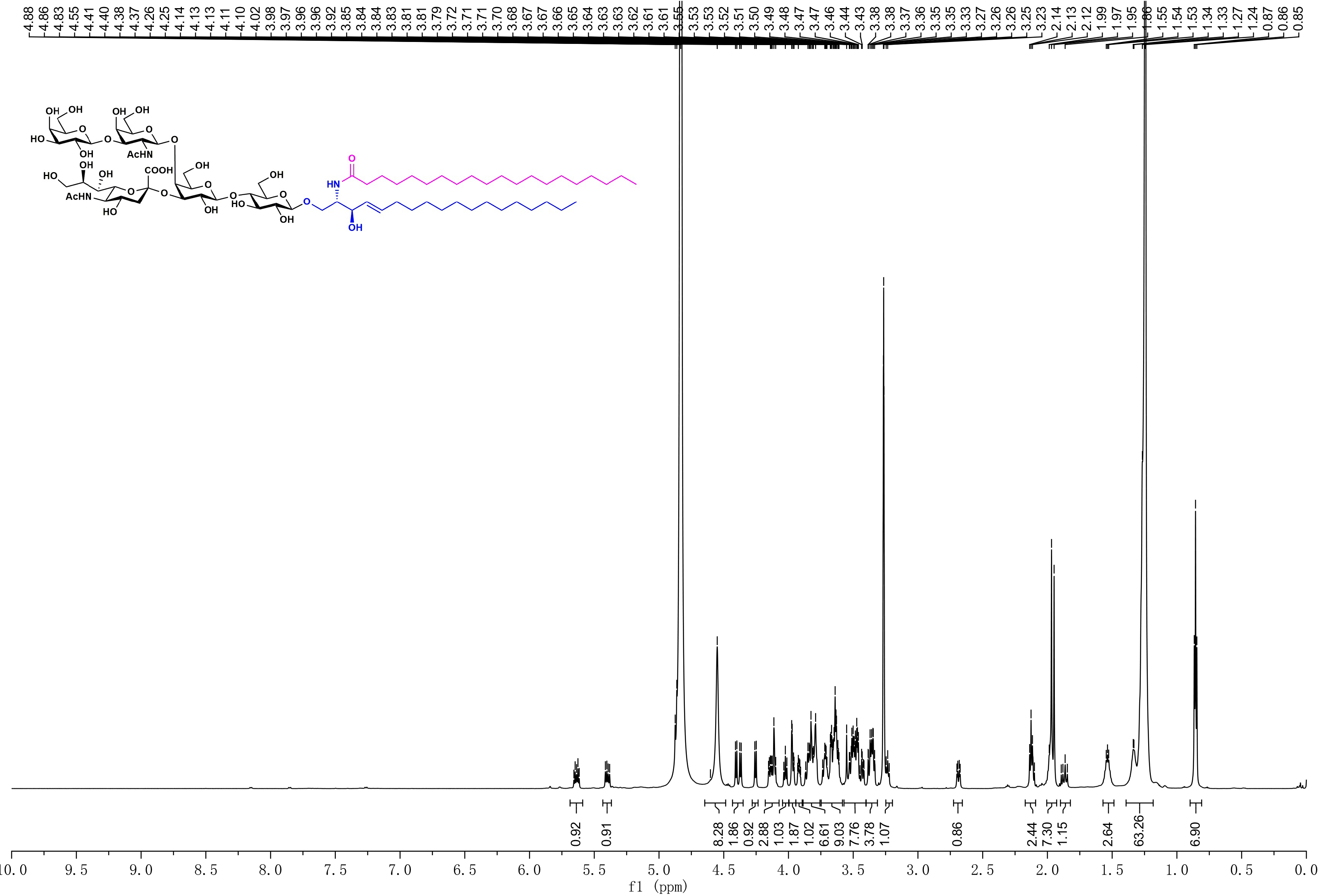


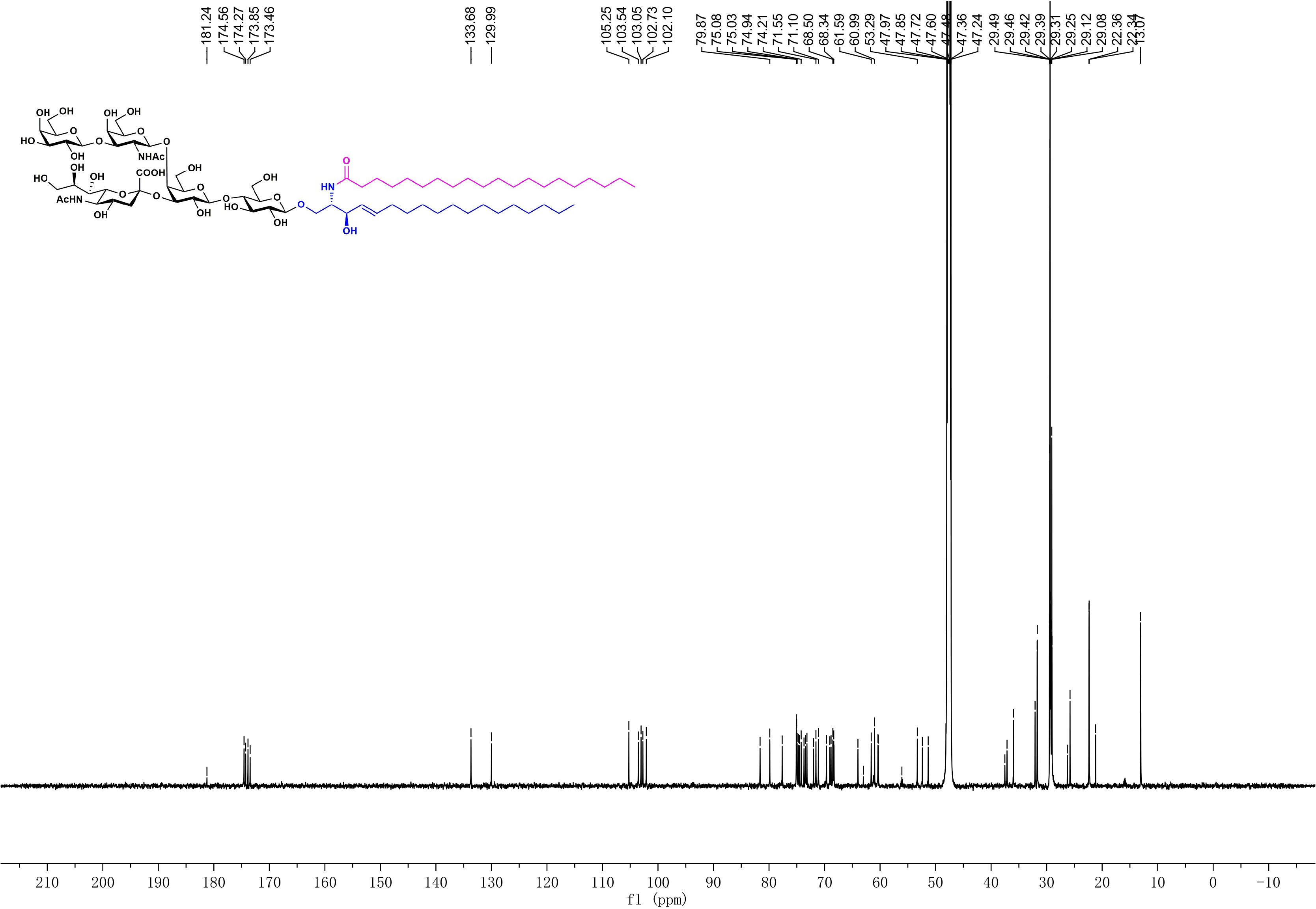


^1^H and ^13^C NMR spectra of GM1 (d18:1/C16:1) **(21)**


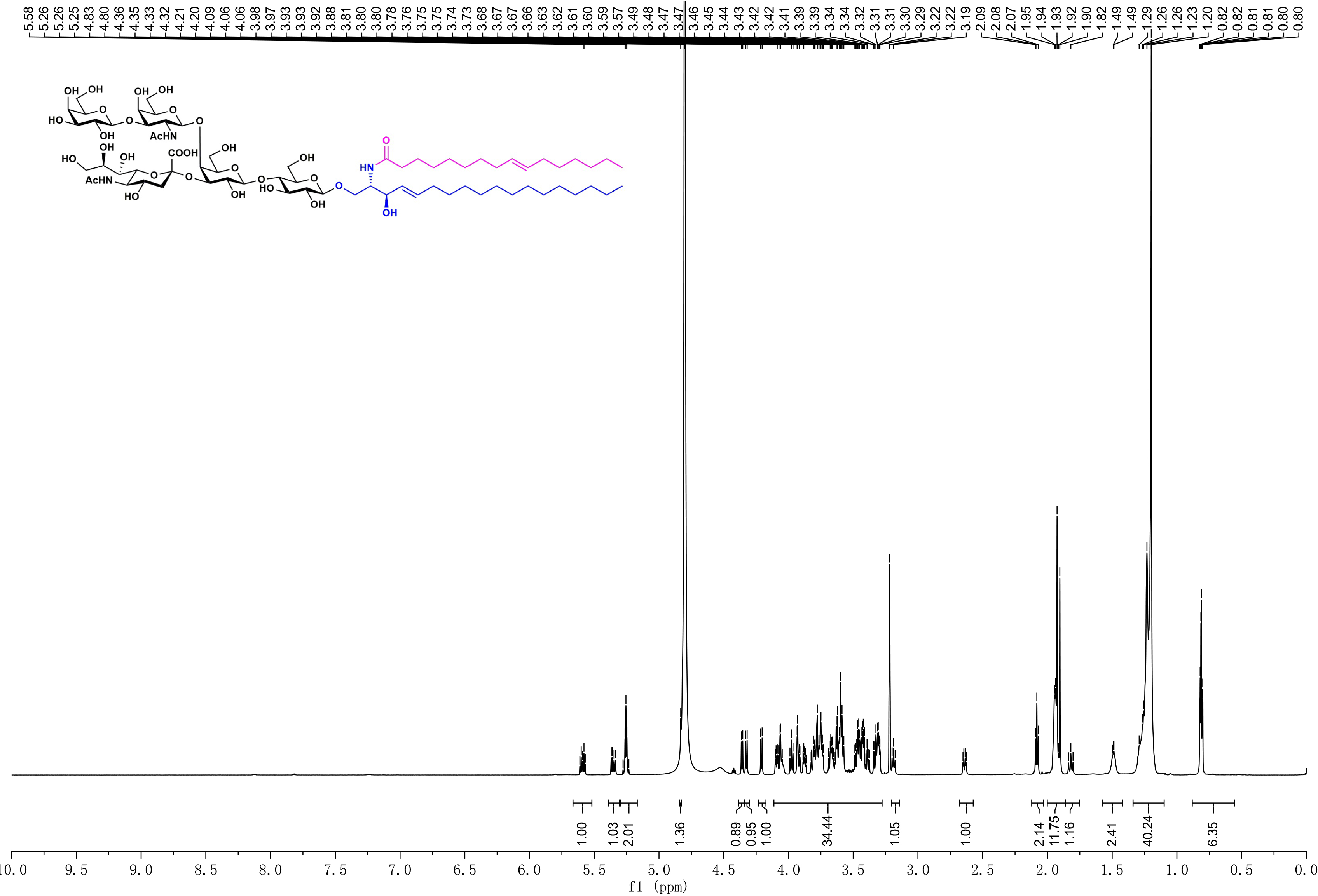


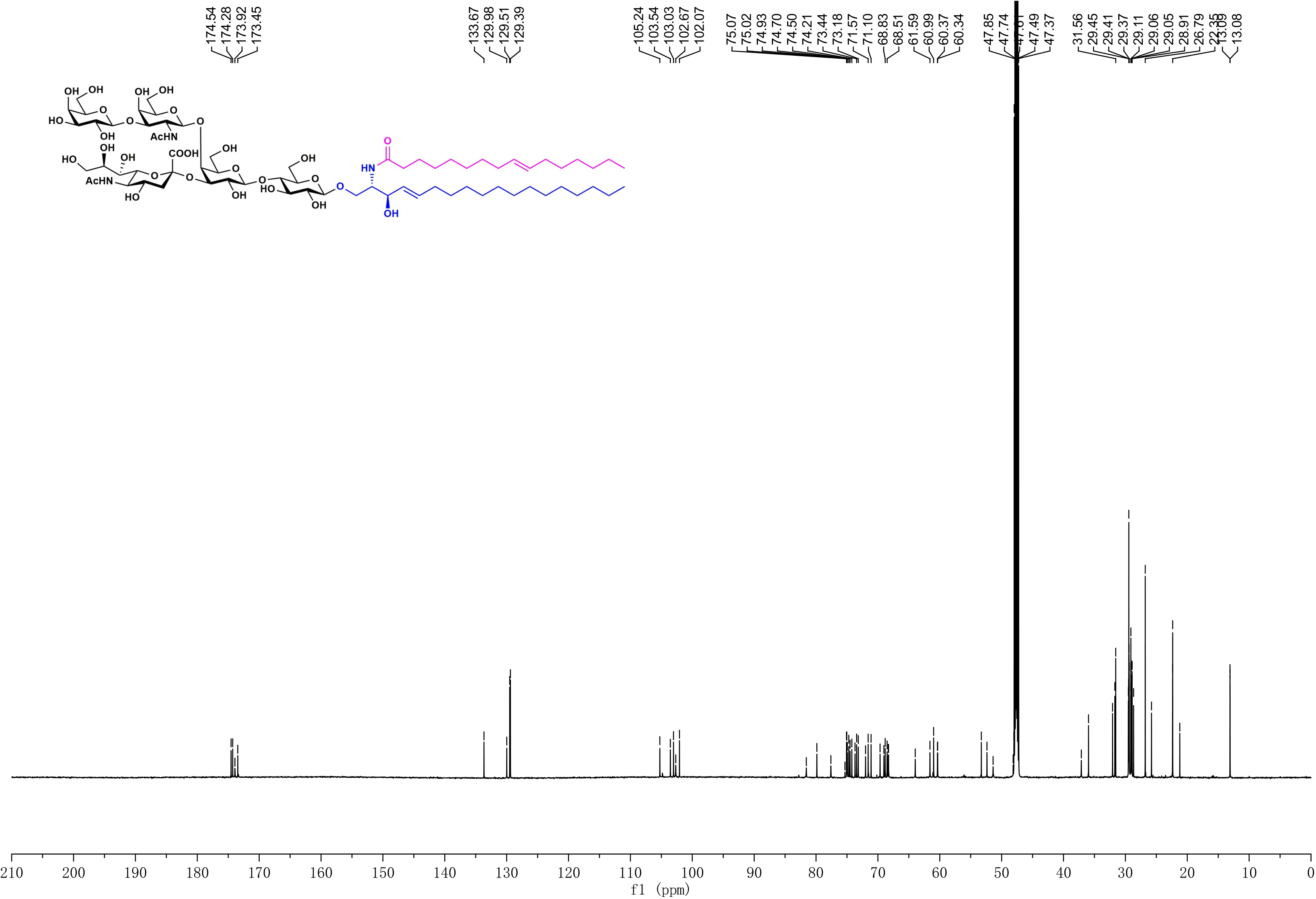


^1^H and ^13^C NMR spectra of GM1 (d18:1/C18:1) **(22)**


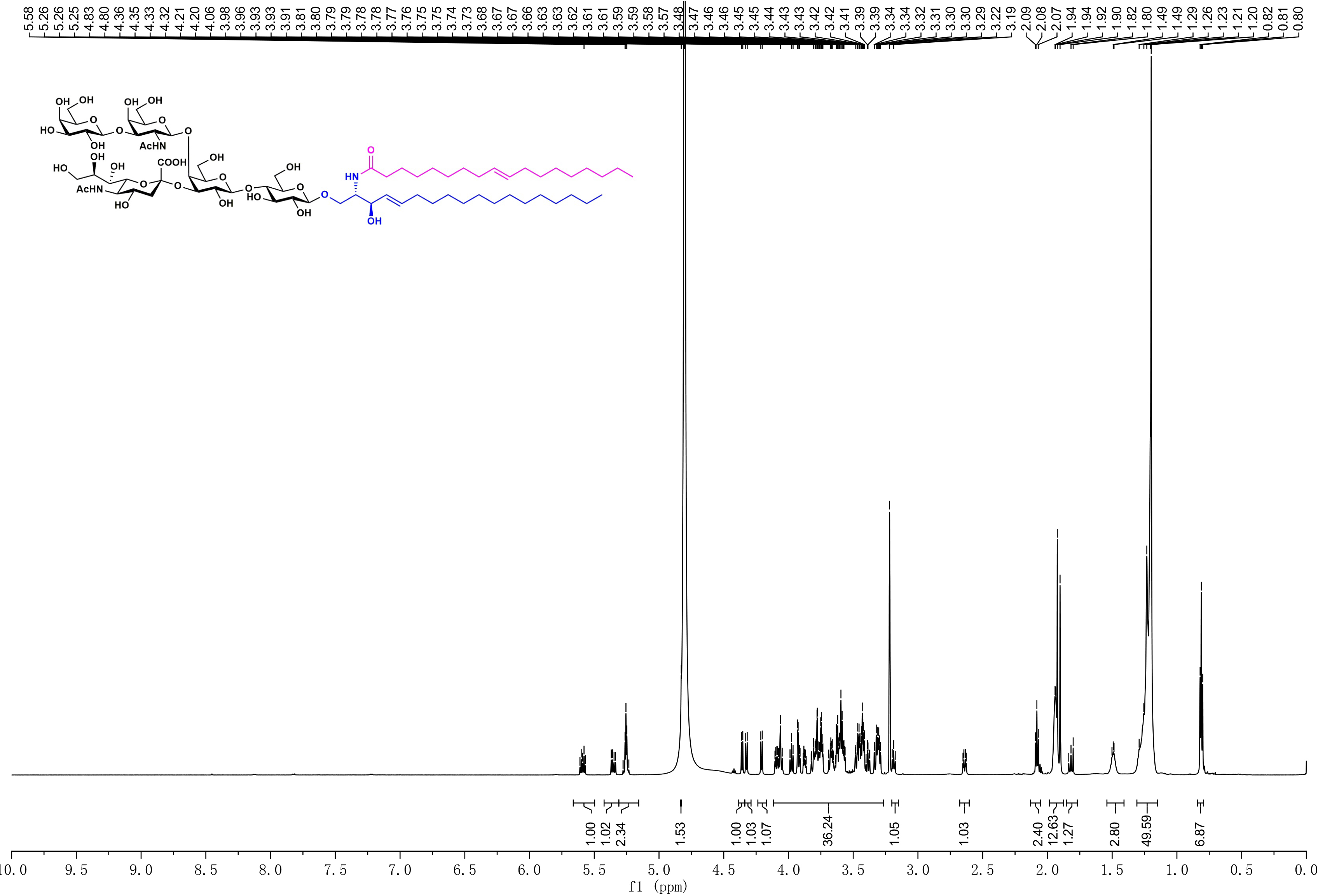


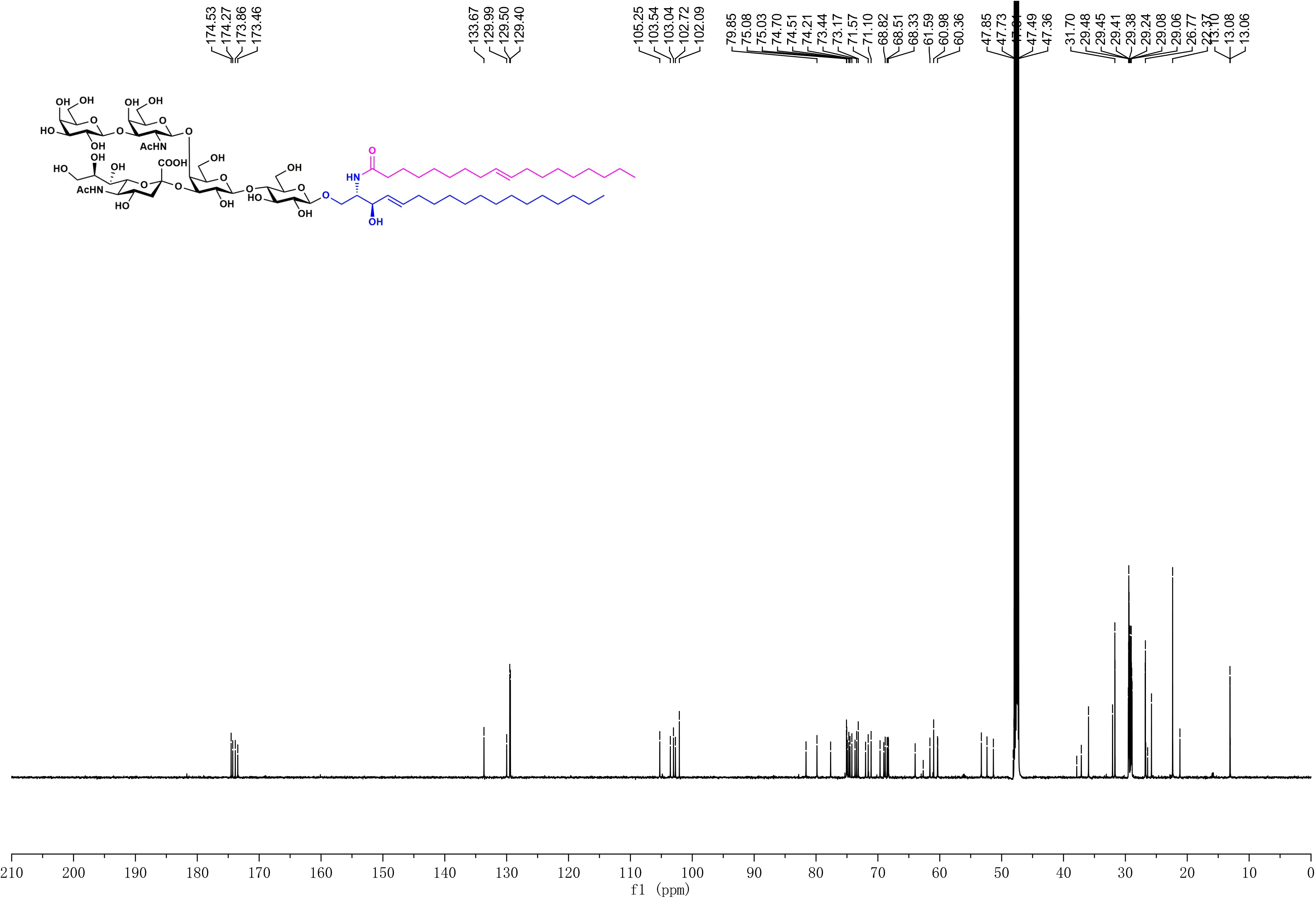


^1^H and ^13^C NMR spectra of GM1 (d20:1/C16:0) **(23)**


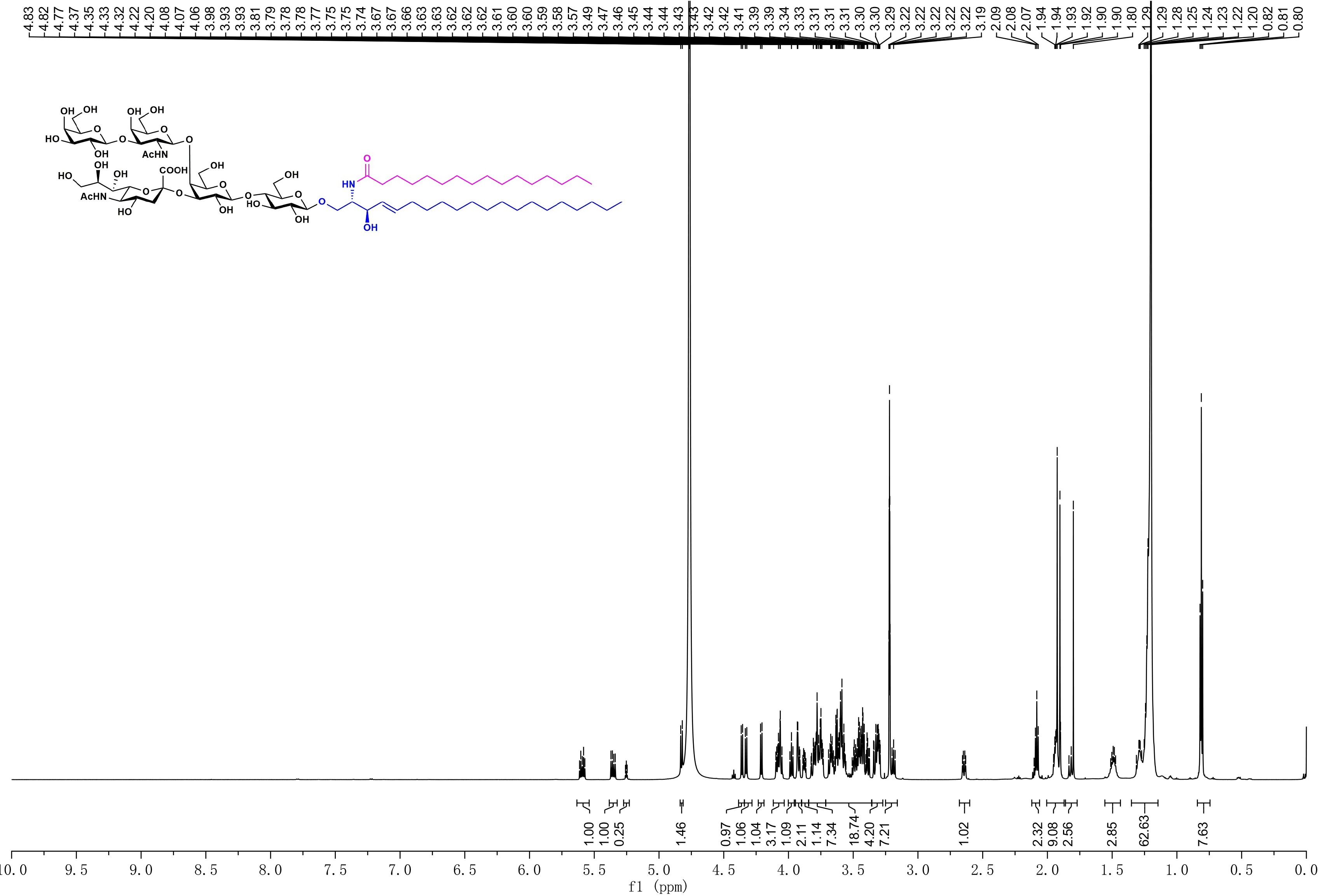


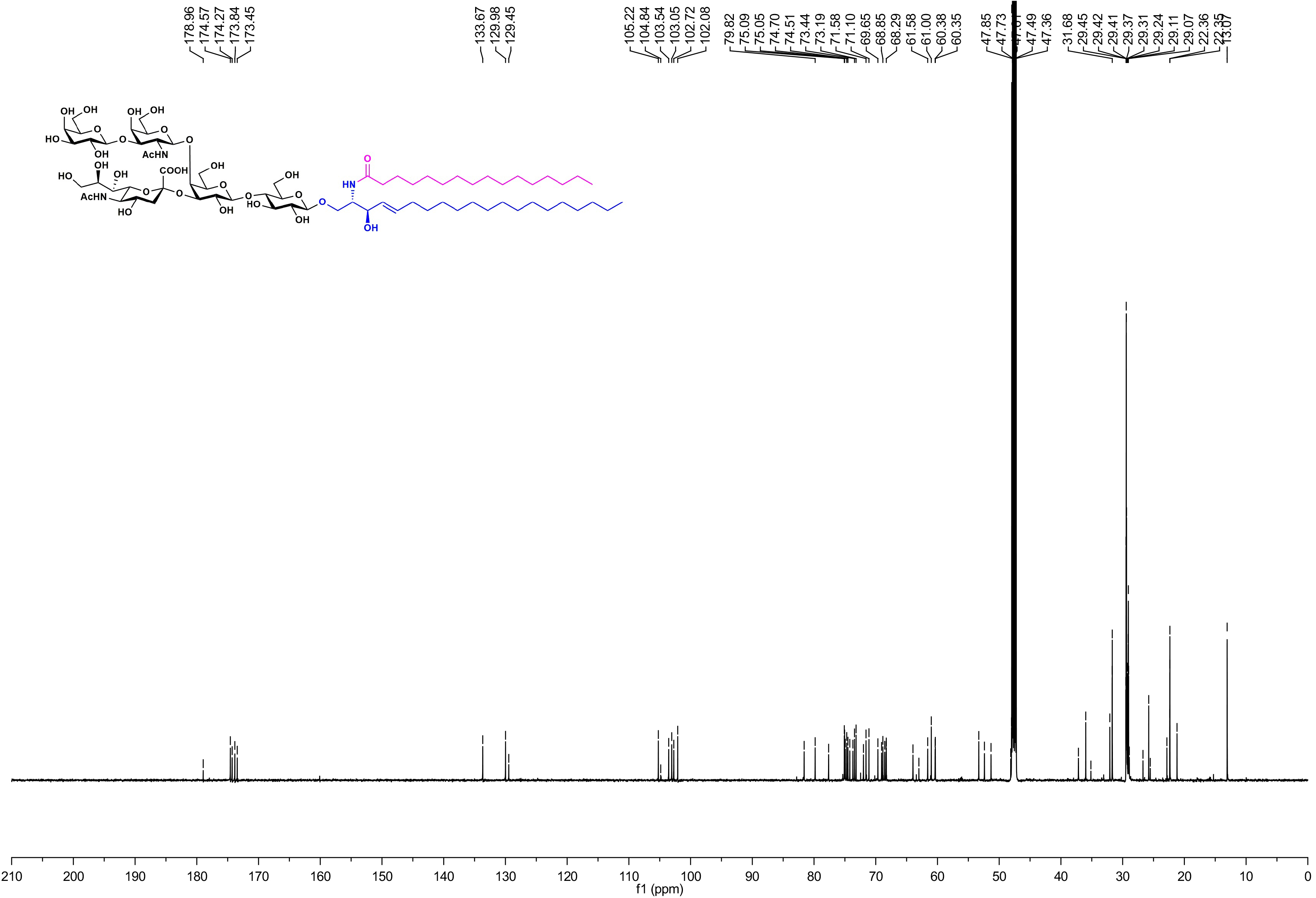


^1^H and ^13^C NMR spectra of GM1 (d20:1/C18:0) **(24)**


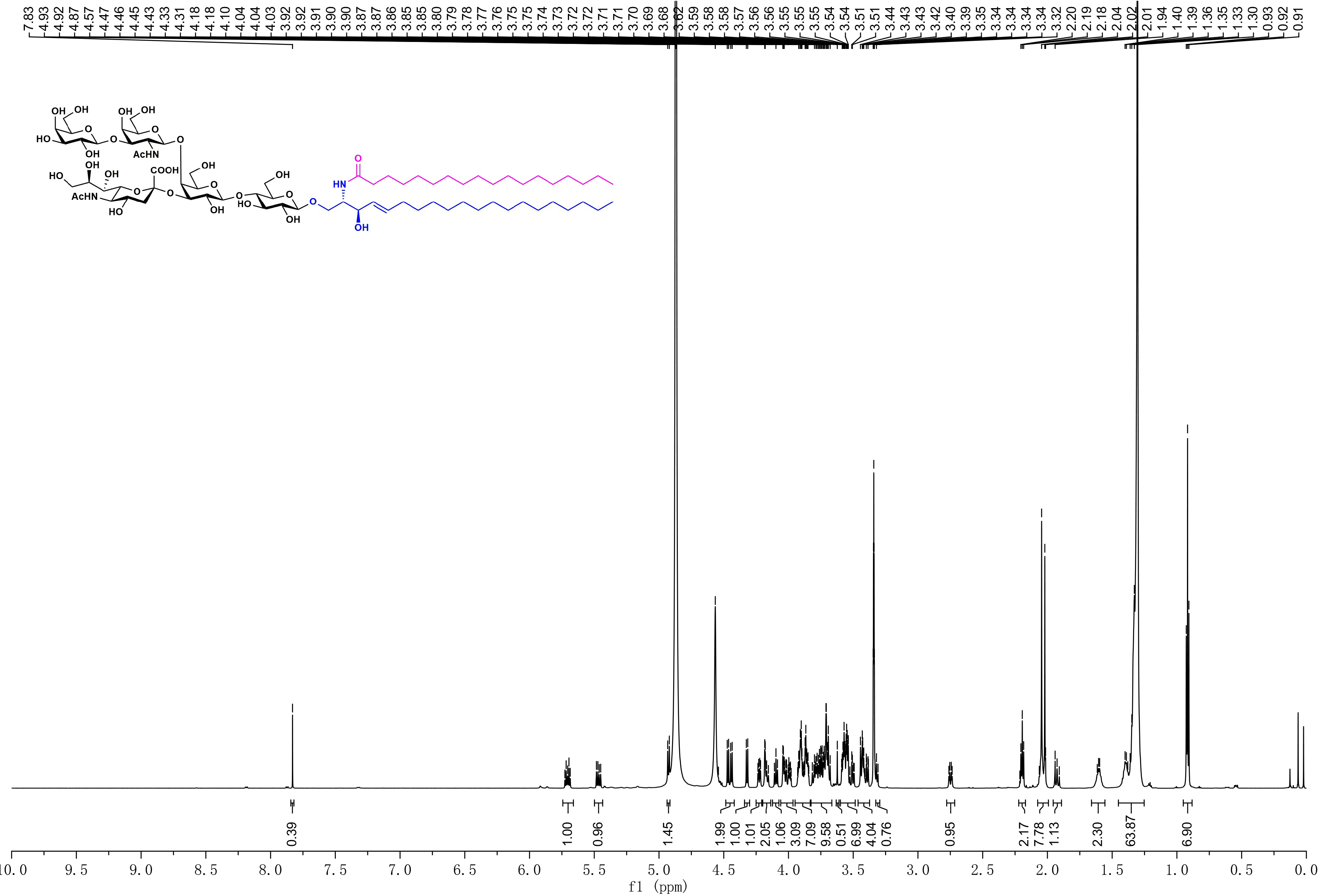


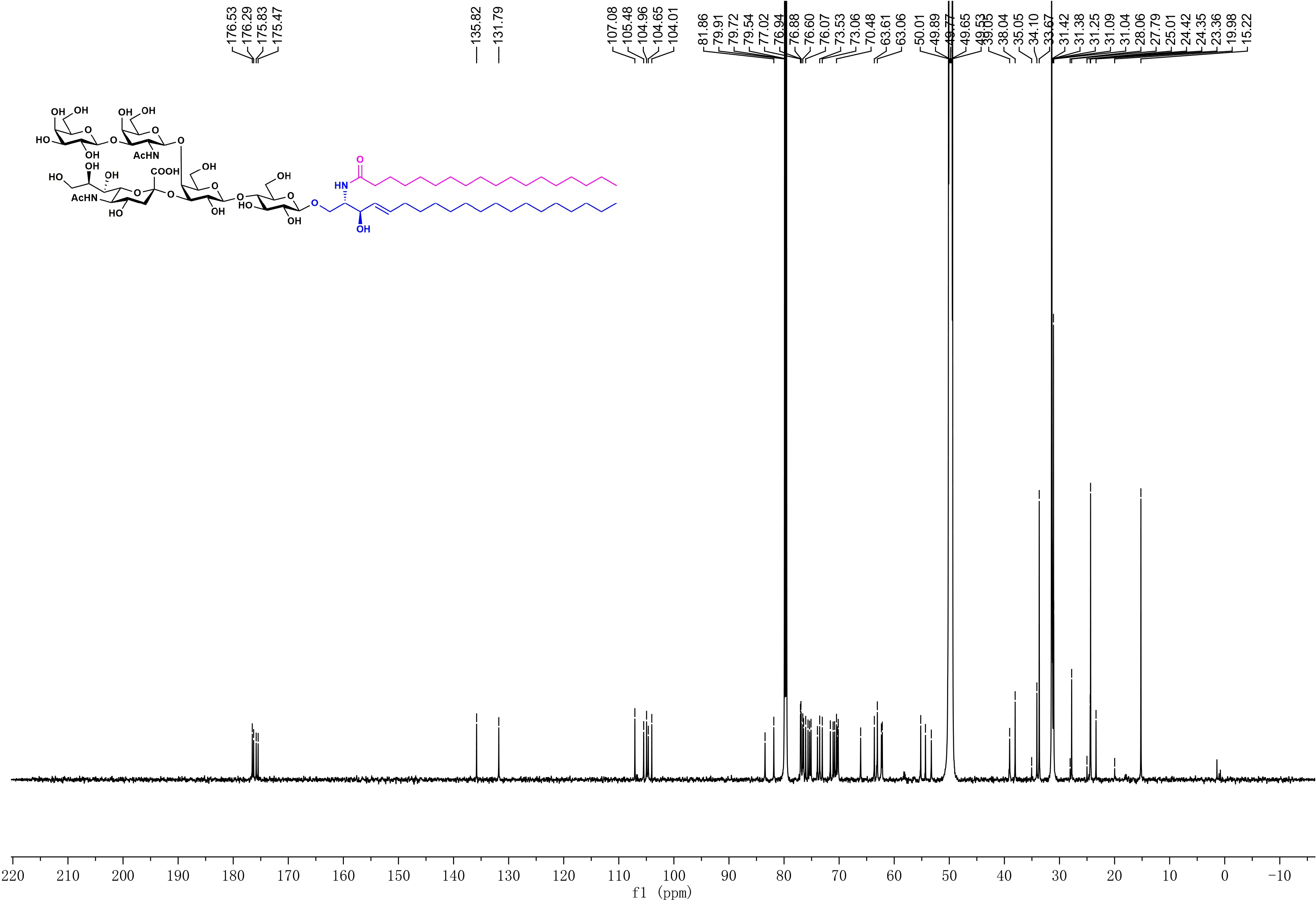


^1^H and ^13^C NMR spectra of GM1 (d20:1/C20:0) **(25)**


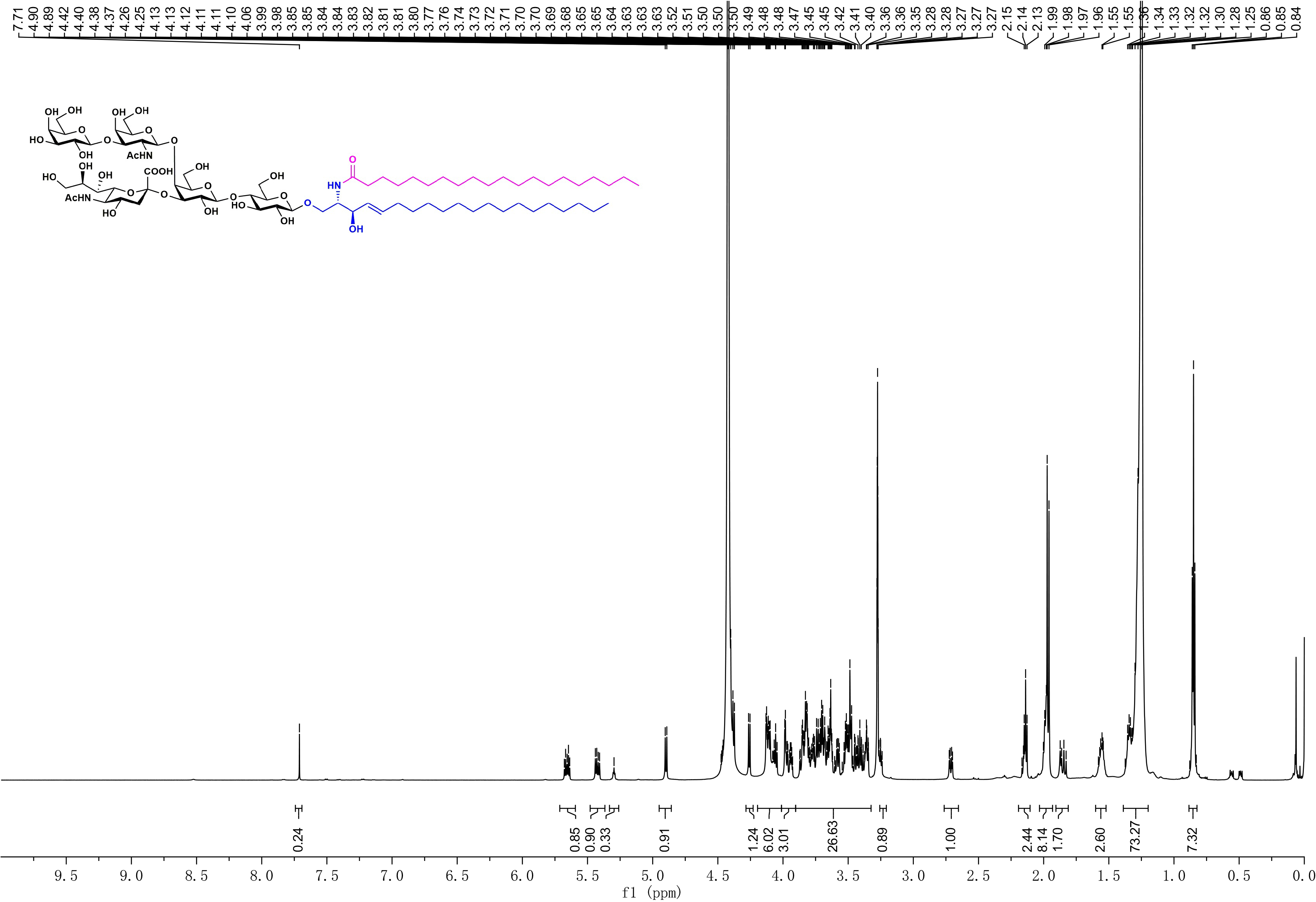


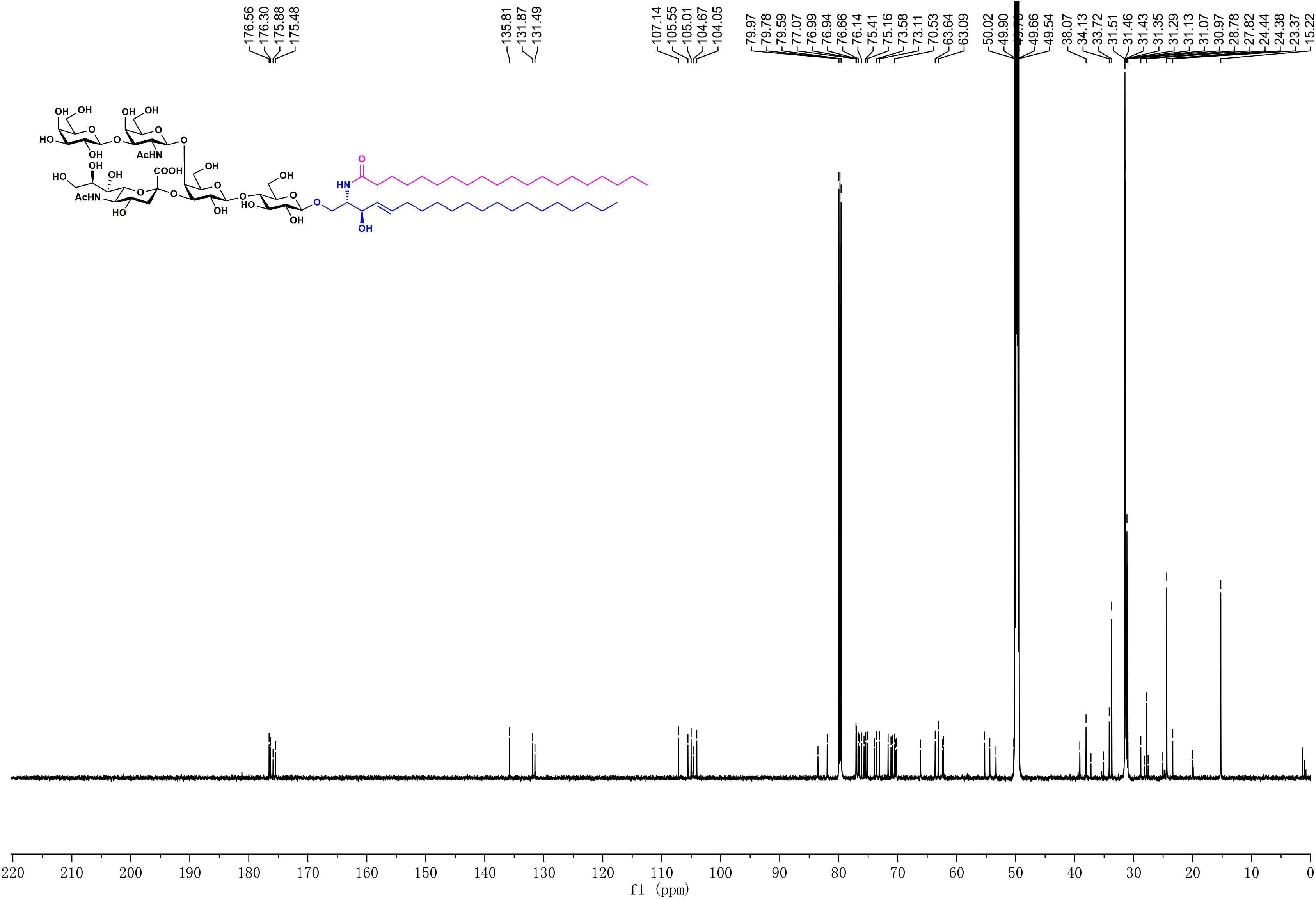


^1^H and ^13^C NMR spectra of GM1 (d20:1/C16:1) **(26)**


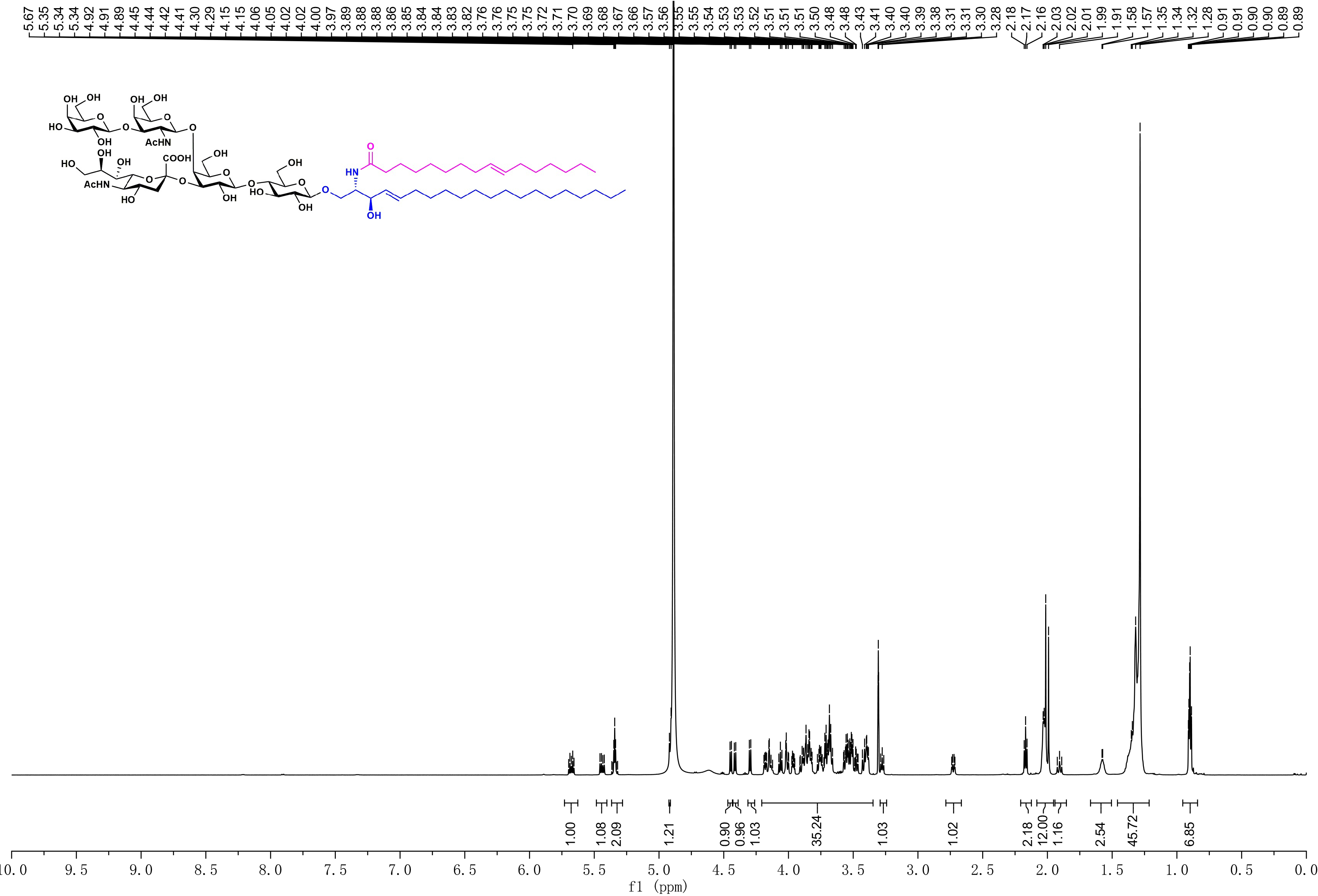


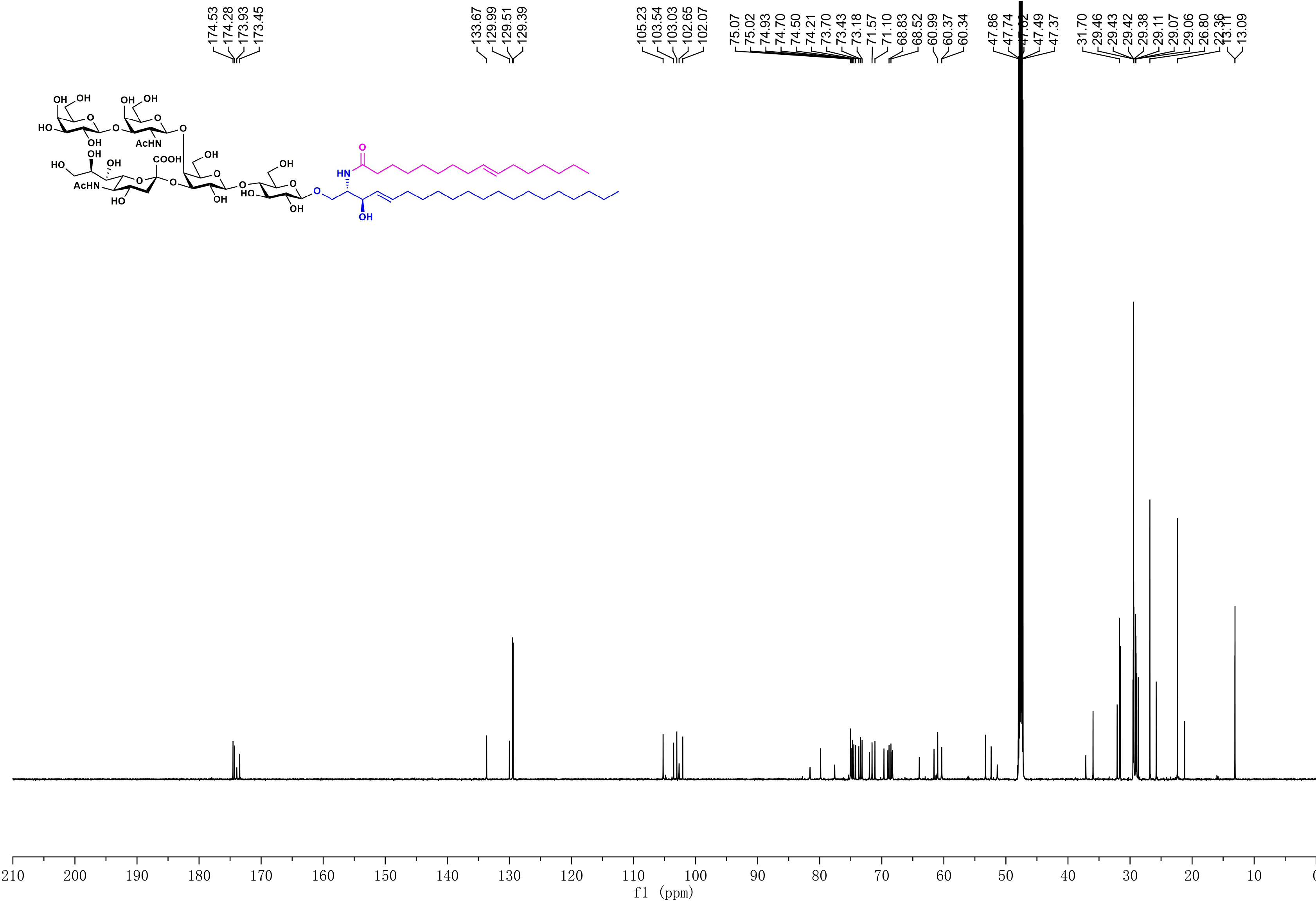


^1^H and ^13^C NMR spectra of GM1 (d20:1/C18:1) **(27)**


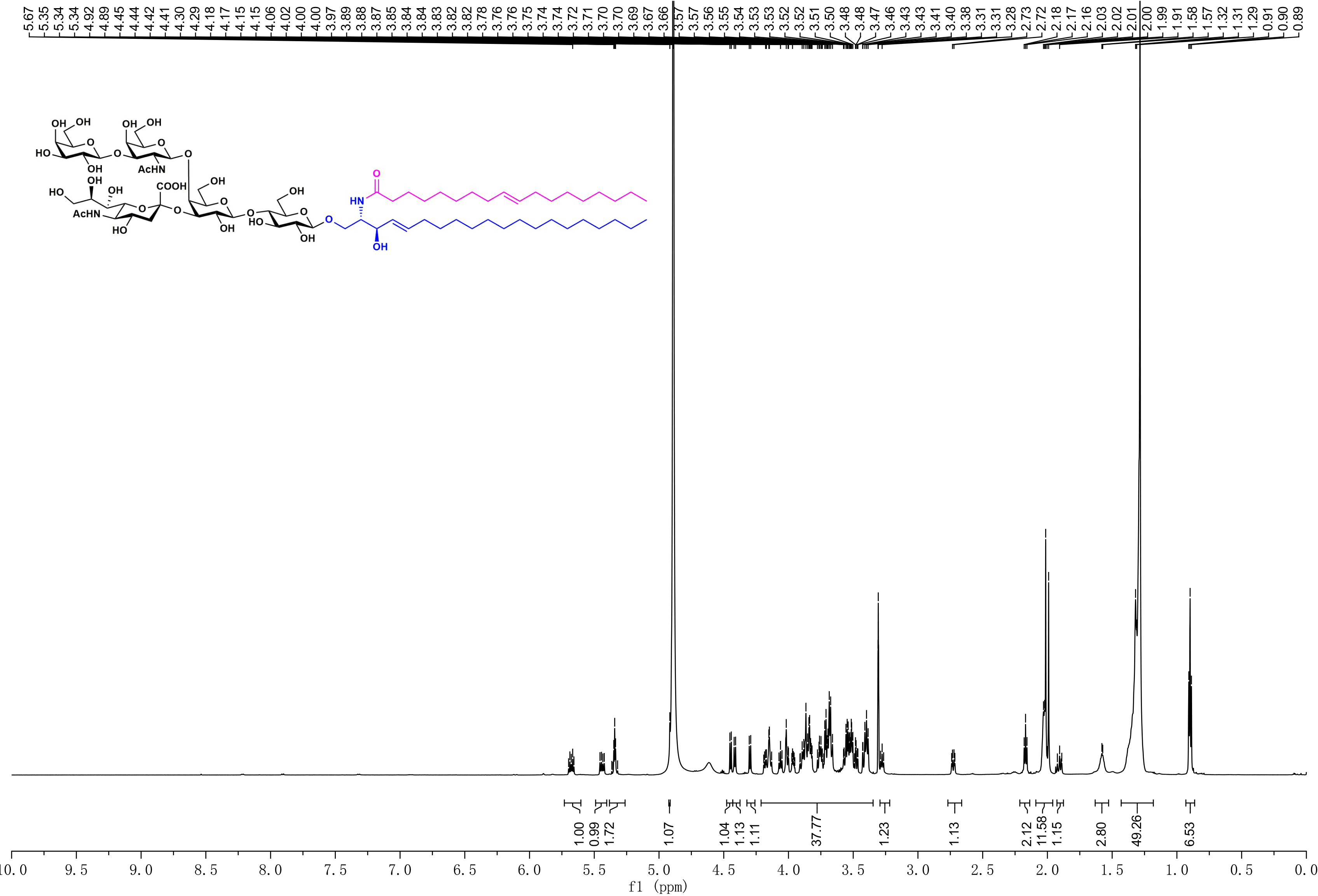


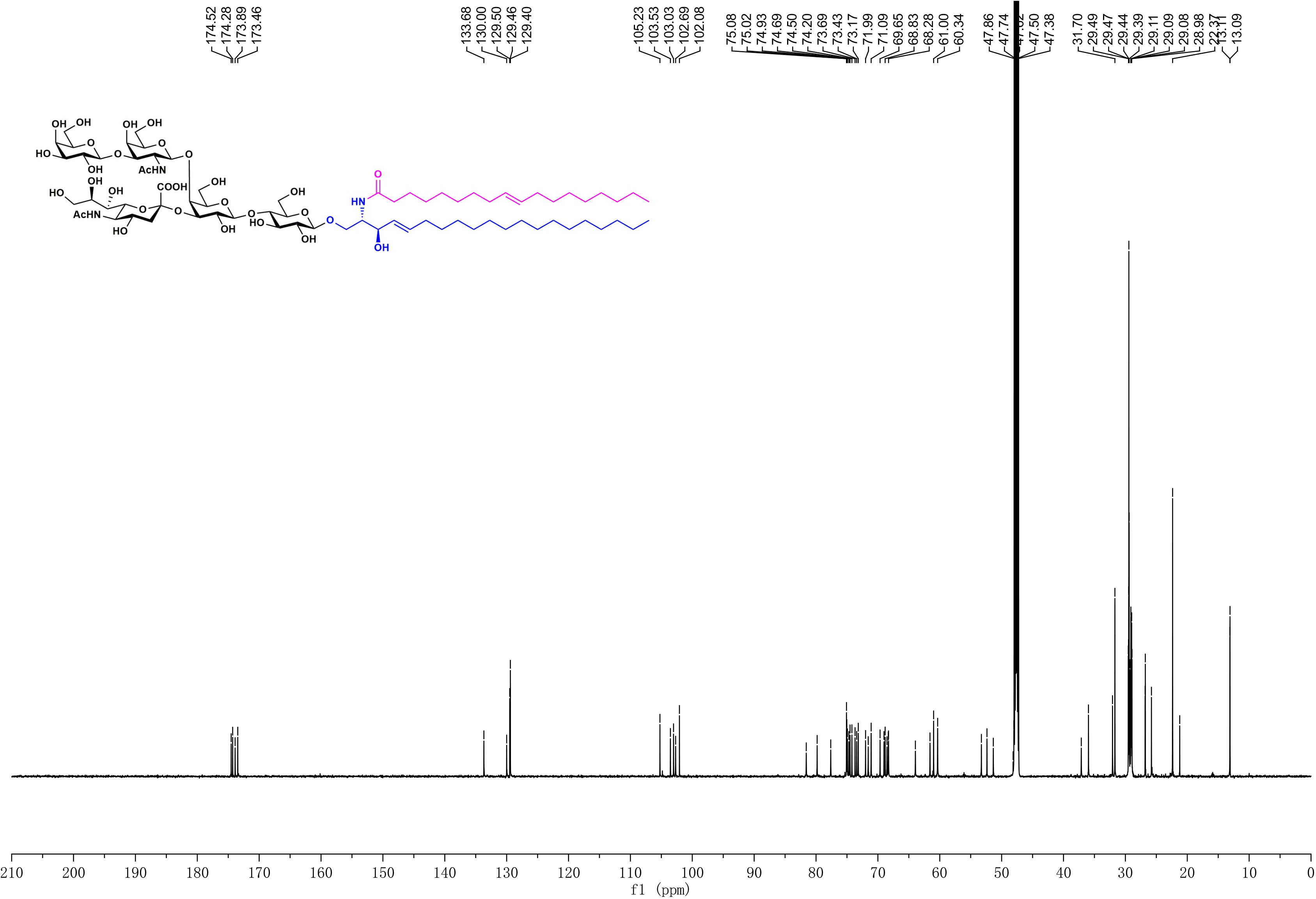

Supplement: Supplementary file 4 — Supplementary Data 1 [file 42004_2024_1102_MOESM4_ESM.docx]
