# Supplementary material for: A modular chemoenzymatic cascade strategy for the structure-customized assembly of ganglioside analogs
Source: Commun Chem. 2024 Jan 18;7:17. doi: 10.1038/s42004-024-01102-9 (PMC10796935; doi:10.1038/s42004-024-01102-9)
Supplement: Supplementary file 5 — Reporting Summary [file 42004_2024_1102_MOESM5_ESM.pdf]

nature portfolio

Corresponding author(s): Guang-Yu Yang

Last updated by author(s): Jan 4, 2024

Reporting Summary

Nature Portfolio wishes to improve the reproducibility of the work that we publish. This form provides structure for consistency and transparency in reporting. For further information on Nature Portfolio policies, see our Editorial Policies and the Editorial Policy Checklist.

Statistics

For all statistical analyses, confirm that the following items are present in the figure legend, table legend, main text, or Methods section.

n/a	Confirmed	
☐	☒	The exact sample size (n) for each experimental group/condition, given as a discrete number and unit of measurement
☐	☒	A statement on whether measurements were taken from distinct samples or whether the same sample was measured repeatedly
☐	☒	The statistical test(s) used AND whether they are one- or two-sided<br>*Only common tests should be described solely by name; describe more complex techniques in the Methods section.*
☐	☒	A description of all covariates tested
☐	☒	A description of any assumptions or corrections, such as tests of normality and adjustment for multiple comparisons
☐	☒	A full description of the statistical parameters including central tendency (e.g. means) or other basic estimates (e.g. regression coefficient) AND variation (e.g. standard deviation) or associated estimates of uncertainty (e.g. confidence intervals)
☐	☒	For null hypothesis testing, the test statistic (e.g. F, t, r) with confidence intervals, effect sizes, degrees of freedom and P value noted<br>*Give P values as exact values whenever suitable.*
☒	☐	For Bayesian analysis, information on the choice of priors and Markov chain Monte Carlo settings
☐	☒	For hierarchical and complex designs, identification of the appropriate level for tests and full reporting of outcomes
☐	☒	Estimates of effect sizes (e.g. Cohen's d, Pearson's r), indicating how they were calculated

Our web collection on statistics for biologists contains articles on many of the points above.

Software and code

Policy information about availability of computer code

Data collection	We stated that no software was used.
Data analysis	We stated that no software was used.

For manuscripts utilizing custom algorithms or software that are central to the research but not yet described in published literature, software must be made available to editors and reviewers. We strongly encourage code deposition in a community repository (e.g. GitHub). See the Nature Portfolio guidelines for submitting code & software for further information.

Data

Policy information about availability of data

All manuscripts must include a data availability statement. This statement should provide the following information, where applicable:

- Accession codes, unique identifiers, or web links for publicly available datasets
- A description of any restrictions on data availability
- For clinical datasets or third party data, please ensure that the statement adheres to our policy

All data needed to support the conclusion are present in the article, supplementary information and supplementary data. Supplementary information comprehensively provide the experimental procedures involved in various catalytic modules of the MOCECA strategy including chemical synthesis, streamlined multienzyme glycosylation, enzymatic transglycosylation, enzymatic acylation. The experimental procedures for component identification of GM1 commercialized drugs are also described. All data and spectra of HRMS, and HPLC for compounds in the article are listed in supplementary information. All NMR spectra for

compounds in the article are listed in supplementary data.

Research involving human participants, their data, or biological material

Policy information about studies with human participants or human data. See also policy information about sex, gender (identity/presentation), and sexual orientation and race, ethnicity and racism.

Reporting on sex and gender	The main content of this study is the customized preparation of ganglioside analogues using chemical enzymatic synthesis technology, without involving the collection of this information.
Reporting on race, ethnicity, or other socially relevant groupings	The main content of this study is the customized preparation of ganglioside analogues using chemical enzymatic synthesis technology, without involving the collection of this information.
Population characteristics	The main content of this study is the customized preparation of ganglioside analogues using chemical enzymatic synthesis technology, without involving the collection of this information.
Recruitment	The main content of this study is the customized preparation of ganglioside analogues using chemical enzymatic synthesis technology, without involving the collection of this information.
Ethics oversight	The main content of this study is the customized preparation of ganglioside analogues using chemical enzymatic synthesis technology, without involving the collection of this information.

Note that full information on the approval of the study protocol must also be provided in the manuscript.

Field-specific reporting

Please select the one below that is the best fit for your research. If you are not sure, read the appropriate sections before making your selection.

☒ Life sciences ☐ Behavioural & social sciences ☐ Ecological, evolutionary & environmental sciences

For a reference copy of the document with all sections, see nature.com/documents/nr-reporting-summary-flat.pdf

Life sciences study design

All studies must disclose on these points even when the disclosure is negative.

Sample size	In order to assess the neurite outgrowth activities of MOCECA-synthesized GM1 and GM1βSph analogues in Neuro2a cells, we have recorded the length of the longest neurite and the proportion of cells bearing a neurite for 30 cells in each group. Significant differences among groups were calculated using Newman-Keuls multiple comparison test following one-way ANOVA analysis.
Data exclusions	No data were excluded from the analyses.
Replication	Each result of of the neurobiological experiments has been repeated independently for three times to verify the reproducibility of the experimental findings in our research. All repeated attempts were successful.
Randomization	All samples used in the study were from the same Neuro2a cell line. Therefore, this is not relevant to our study.
Blinding	All samples used in the study were from the same Neuro2a cell line. Therefore, this is not relevant to our study.

Reporting for specific materials, systems and methods

We require information from authors about some types of materials, experimental systems and methods used in many studies. Here, indicate whether each material, system or method listed is relevant to your study. If you are not sure if a list item applies to your research, read the appropriate section before selecting a response.

Materials & experimental systems

n/a	Involved in the study
☒	☐ Antibodies
☒	☐ Eukaryotic cell lines
☒	☐ Palaeontology and archaeology
☐	☒ Animals and other organisms
☒	☐ Clinical data
☒	☐ Dual use research of concern
☒	☐ Plants

Methods

n/a	Involved in the study
☒	☐ ChIP-seq
☒	☐ Flow cytometry
☒	☐ MRI-based neuroimaging

Animals and other research organisms

Policy information about studies involving animals; ARRIVE guidelines recommended for reporting animal research, and Sex and Gender in Research

Laboratory animals	This study did not involve laboratory animals.
Wild animals	We stated that the study did not involve wild animals.
Reporting on sex	The biological samples used in this study were all from Neuro2a cell lines and did not involve sex-based analysis.
Field-collected samples	We stated that the study did not involve samples collected from the field.
Ethics oversight	The biological samples used in this study were all from Neuro2a cell lines. No ethical approval was required in this study.

Note that full information on the approval of the study protocol must also be provided in the manuscript.

Plants

Seed stocks	*Report on the source of all seed stocks or other plant material used. If applicable, state the seed stock centre and catalogue number. If plant specimens were collected from the field, describe the collection location, date and sampling procedures.*
Novel plant genotypes	*Describe the methods by which all novel plant genotypes were produced. This includes those generated by transgenic approaches, gene editing, chemical/radiation-based mutagenesis and hybridization. For transgenic lines, describe the transformation method, the number of independent lines analyzed and the generation upon which experiments were performed. For gene-edited lines, describe the editor used, the endogenous sequence targeted for editing, the targeting guide RNA sequence (if applicable) and how the editor was applied.*
Authentication	*Describe any authentication procedures for each seed stock used or novel genotype generated. Describe any experiments used to assess the effect of a mutation and, where applicable, how potential secondary effects (e.g. second site T-DNA insertions, mosiacism, off-target gene editing) were examined.*
